# Effect of Usual Medical Care Plus Chiropractic Care vs Usual Medical Care Alone on Pain and Disability Among US Service Members With Low Back Pain: A Comparative Effectiveness Clinical Trial

**DOI:** 10.1001/jamanetworkopen.2018.0105

**Published:** 2018-05-18

**Authors:** Christine M. Goertz, Cynthia R. Long, Robert D. Vining, Katherine A. Pohlman, Joan Walter, Ian Coulter

**Affiliations:** 1 Palmer College of Chiropractic, Palmer Center for Chiropractic Research, Davenport, Iowa; 2 Parker University Research Institute, Dallas, Texas; 3 Samueli Institute for Information Biology, Silver Spring, Maryland; 4 RAND Corporation, Santa Monica, California

## Abstract

**Importance:**

It is critically important to evaluate the effect of nonpharmacological treatments on low back pain and associated disability.

**Objective:**

To determine whether the addition of chiropractic care to usual medical care results in better pain relief and pain-related function when compared with usual medical care alone.

**Design, Setting, and Participants:**

A 3-site pragmatic comparative effectiveness clinical trial using adaptive allocation was conducted from September 28, 2012, to February 13, 2016, at 2 large military medical centers in major metropolitan areas and 1 smaller hospital at a military training site. Eligible participants were active-duty US service members aged 18 to 50 years with low back pain from a musculoskeletal source.

**Interventions:**

The intervention period was 6 weeks. Usual medical care included self-care, medications, physical therapy, and pain clinic referral. Chiropractic care included spinal manipulative therapy in the low back and adjacent regions and additional therapeutic procedures such as rehabilitative exercise, cryotherapy, superficial heat, and other manual therapies.

**Main Outcomes and Measures:**

Coprimary outcomes were low back pain intensity (Numerical Rating Scale; scores ranging from 0 [no low back pain] to 10 [worst possible low back pain]) and disability (Roland Morris Disability Questionnaire; scores ranging from 0-24, with higher scores indicating greater disability) at 6 weeks. Secondary outcomes included perceived improvement, satisfaction (Numerical Rating Scale; scores ranging from 0 [not at all satisfied] to 10 [extremely satisfied]), and medication use. The coprimary outcomes were modeled with linear mixed-effects regression over baseline and weeks 2, 4, 6, and 12.

**Results:**

Of the 806 screened patients who were recruited through either clinician referrals or self-referrals, 750 were enrolled (250 at each site). The mean (SD) participant age was 30.9 (8.7) years, 175 participants (23.3%) were female, and 243 participants (32.4%) were nonwhite. Statistically significant site × time × group interactions were found in all models. Adjusted mean differences in scores at week 6 were statistically significant in favor of usual medical care plus chiropractic care compared with usual medical care alone overall for low back pain intensity (mean difference, −1.1; 95% CI, −1.4 to −0.7), disability (mean difference, −2.2; 95% CI, −3.1 to −1.2), and satisfaction (mean difference, 2.5; 95% CI, 2.1 to 2.8) as well as at each site. Adjusted odd ratios at week 6 were also statistically significant in favor of usual medical care plus chiropractic care overall for perceived improvement (odds ratio = 0.18; 95% CI, 0.13-0.25) and self-reported pain medication use (odds ratio = 0.73; 95% CI, 0.54-0.97). No serious related adverse events were reported.

**Conclusions and Relevance:**

Chiropractic care, when added to usual medical care, resulted in moderate short-term improvements in low back pain intensity and disability in active-duty military personnel. This trial provides additional support for the inclusion of chiropractic care as a component of multidisciplinary health care for low back pain, as currently recommended in existing guidelines. However, study limitations illustrate that further research is needed to understand longer-term outcomes as well as how patient heterogeneity and intervention variations affect patient responses to chiropractic care.

**Trial Registration:**

ClinicalTrials.gov Identifier: NCT01692275

## Key Points

**Question:**

What is the effect of adding chiropractic care to usual medical care for patients with low back pain?

**Findings:**

In this comparative effectiveness clinical trial among active-duty US military personnel, patients who received usual medical care plus chiropractic care reported a statistically significant moderate improvement in low back pain intensity and disability at 6 weeks compared with those who received usual care alone.

**Meaning:**

This trial supports the inclusion of chiropractic care as a component of multidisciplinary health care for low back pain, as currently recommended in existing guidelines.

## Teaser

This clinical trial compares the effects of chiropractic care added to usual medical care vs usual medical care alone on pain relief and pain-related function among active-duty US military personnel.

## Introduction

Musculoskeletal disorders are the second leading cause of disability worldwide, led by low back pain (LBP), with an estimated LBP prevalence among US adults of 20%.^1,2,3^ The direct costs of back pain in the United States in 2010 were $34 billion,^4^ with additional indirect costs including lost workplace productivity estimated at $200 billion.^5^ In the US military, LBP is one of the most common reasons members seek medical care^6^ and one of the most likely conditions to interrupt combat duty.^6,7^ Common medical therapies for LBP, including nonsteroidal anti-inflammatory drugs, opioids, spinal fusions, and epidural steroid injections, demonstrate limited effectiveness^8,9,10^; furthermore, many of these treatments have unacceptably high risk profiles.^8,11,12,13,14^

The US opioid crisis^15,16^ creates an urgent need to evaluate cost-effective and low-risk nonpharmacological treatments. One option is chiropractic care. Doctors of chiropractic provide conservative care focused on diagnosis, treatment, comanagement, or referral for musculoskeletal conditions, including LBP.^17^ The primary therapeutic procedure used by doctors of chiropractic is spinal manipulative therapy.^18^

The use of chiropractic care is common, with annual rates among US adults estimated between 8% and 14%.^19,20^ Current guidelines recommend the use of spinal manipulative therapy and/or chiropractic care for LBP.^21,22^ Although a previous pilot study of chiropractic care for active-duty US military patients with acute LBP showed promise,^23^ and chiropractic care is available at 66 military health treatment facilities worldwide,^24^ significant gaps in knowledge remain in military populations. These populations tend to be younger and more diverse in terms of race and ethnicity than those included in previous trials on spinal manipulation.^25^ This multisite, pragmatic clinical trial begins to address these gaps by investigating whether adding chiropractic care to usual medical care (UMC) improves outcomes for patients with LBP at military treatment facilities.

## Methods

### Study Design, Setting, and Participants

A detailed study protocol was previously published,^26^ and the trial protocol is available in Supplement 1. This pragmatic, prospective, multisite, parallel-group comparative effectiveness clinical trial with adaptive allocation was conducted at 2 large military medical centers in major metropolitan areas (Walter Reed National Military Medical Center [hereafter referred to as “Walter Reed”], Bethesda, Maryland; and Naval Medical Center San Diego [hereafter referred to as “San Diego”], San Diego, California) and at 1 smaller hospital at a military training site (Naval Hospital Pensacola [hereafter referred to as “Pensacola”], Pensacola, Florida). Active-duty US military participants aged 18 to 50 years reporting LBP were eligible. Low back pain of any duration from a nonmusculoskeletal source, the presence of a contraindication to spinal manipulative therapy, recent spinal fracture, recent spinal surgery, and a diagnosis of posttraumatic stress disorder were exclusionary. Participants with radiculopathy were eligible if a further diagnostic evaluation or surgical referral was not necessary. Participants either were referred to the study by physicians who diagnose or manage LBP or were self-referred through posted advertisements. Physicians or Independent Duty Corpsmen conducted a screening examination to assess eligibility. The trial was approved by each site and participating institution’s institutional review board and was overseen by an independent data and safety monitoring committee. All participants provided written informed consent and were not compensated for participation. The study followed the Consolidated Standards of Reporting Trials (CONSORT) reporting guideline.

### Allocation

Participants were allocated in equal proportions to UMC with chiropractic care or to UMC alone for 6 weeks, stratified by site. The data coordinating center programmed an adaptive computer-generated minimization algorithm to balance group assignment on sex, age, LBP duration, and worst pain intensity in the past 24 hours at baseline. Study personnel accessed the web application to make group assignments, and future allocations were concealed.

### Study Interventions

#### Usual Medical Care

In this pragmatic trial, UMC in both groups included any care recommended or prescribed by nonchiropractic military clinicians to treat LBP. Options included self-management advice, pharmacologic pain management, physical therapy, or pain clinic referral. Participants allocated to UMC alone were asked to avoid receiving chiropractic care for the active care period unless directed by their clinician. In both groups, frequency of treatment visits and procedures were determined individually based on the participant’s diagnosis or condition, response to care, and scheduling availability.

#### UMC With Chiropractic Care

Participants allocated to UMC with chiropractic care had UMC in addition to as many as 12 chiropractic visits during the active care period. The primary chiropractic procedure was spinal manipulative therapy in the low back and adjacent regions.^18^ Treatment decisions regarding manipulation type, location, and direction were based on patient diagnoses. Other factors included patient preference, prior response to care, paraspinal muscle hypertonicity, spinal joint hypomobility, and imaging findings. Additional therapeutic procedures may have included rehabilitative exercise, interferential current therapy, ultrasound therapy, cryotherapy, superficial heat, and other manual therapies.

### Blinding

It was not possible to blind treating clinicians or participants to treatment assignment. However, all key study personnel and data analysts were blinded.

### Outcomes

Sociodemographic and clinical information was obtained at baseline. Participants were classified by race and ethnicity based on self-report. These data were collected to determine the generalizability of study findings, which is important for studies of chiropractic care as previous trials have included primarily white participants not of Latino descent. Usual medical care data on physical therapy or specialty referrals and prescription medications for spine-related pain as well as *Current Procedural Terminology* codes describing treatments delivered by the doctor of chiropractic were abstracted from the electronic health record.

Primary outcomes (eTable 1 in Supplement 2) were measured at baseline and 2, 4, 6, and 12 weeks after baseline via online self-report questions through an electronic data capture system.^26^ Primary and secondary end points were at 6 and 12 weeks, respectively.

#### Coprimary Outcomes

Average LBP intensity during the prior week was assessed by the Numerical Rating Scale (NRS; scores ranging from 0 [no LBP] to 10 [worst possible LBP]).^27,28,29^ Functional disability related to LBP was assessed by the Roland Morris Disability Questionnaire (RMDQ; scores ranging from 0-24, with higher scores indicating greater disability).^30^ Primary analyses compared adjusted means between groups at the primary and secondary end points. A secondary responder analysis compared the percentage of patients with at least 30% improvement from baseline at each end point.

#### Secondary Outcomes

Worst LBP intensity during the past 24 hours was assessed using the NRS. Bothersomeness of LBP symptoms in the past week was measured on a scale of 1 (not at all bothersome) to 5 (extremely bothersome).^31^ Pain medication use was collected by asking participants how often they took pain-relieving medication (both prescription and over-the-counter) during the past week (0, 1-2, 3-4, 5-6, or 7 days). Global LBP improvement was assessed by asking participants to rate their perceived LBP improvement since baseline on a 7-point scale (0 indicated completely gone; 6, much worse).^32,33^ Satisfaction with care was assessed with an NRS, with scores ranging from 0 (not at all satisfied) to 10 (extremely satisfied).

### Adverse Events

Adverse events were documented by participants via online self-report questions answered at 2, 4, and 6 weeks, and from project managers who directly queried participants.

### Sample Size

We anticipated that outcomes might vary by site owing to differing patient populations. Therefore, we calculated a sample size of 106 patients per group per site to provide adequate power to detect clinically important between-group differences.^34^ A Bonferroni-adjusted significance level of α = .025 accounted for the coprimary outcome variables, with standard deviations estimated from our pilot study. This provided 92% power to detect a between-group difference of at least 1.2 points on the NRS and 80% power to detect a difference of at least 2.4 points on the RMDQ^35^ at each site. We increased the sample size to 125 patients per group at each site to account for an estimated loss to follow-up of 15% at the week 6 end point.

### Statistical Analysis

The data analysis plan was prespecified and is shown in Supplement 1.^26^ Analyses followed an intention-to-treat approach in which all participants’ data were analyzed according to their original treatment allocation. We used SAS/STAT (release 9.4; SAS Institute Inc) for data analyses. All observed data were used in the analyses. Regression models included terms for time, site, group, and site × group, time × group, and site × time × group interactions, adjusted for sex, age, pain duration, and worst pain during the past 24 hours. For all analyses, if the site × time × group interaction was significant at the α = .05 level, results from the adjusted final models were reported overall and by site.

The coprimary outcome variables were modeled with linear mixed-effects regression over baseline and weeks 2, 4, 6, and 12. Bonferroni-corrected *P* ≤ .025 was used to determine whether between-group differences were statistically significant at weeks 6 and 12. The responder analyses of the coprimary outcome variables were modeled with a modified Poisson regression fit through generalized estimating equations.

Two approaches to sensitivity analyses were used to examine possible effects of missing data on results of coprimary outcome variables. The first assumed that data were missing at random and the second was a tipping-point approach that assumed data were missing not at random. Multiple imputation with the Markov chain Monte Carlo method was used for both methods to impute missing values for the coprimary outcome variables at weeks 2, 4, 6, and 12.

Continuous secondary outcome variables were analyzed with the same linear mixed-effects regression models described earlier (except satisfaction, which was collected through week 6), but *P* ≤ .05 was used to determine whether between-group differences were significant. Pain medication use and perceived global improvement were analyzed over baseline and weeks 2, 4, and 6 with a proportional odds model for ordinal categorical data fit through generalized estimating equations.

## Results

### Participants and Treatment Visits

**Figure 1.**
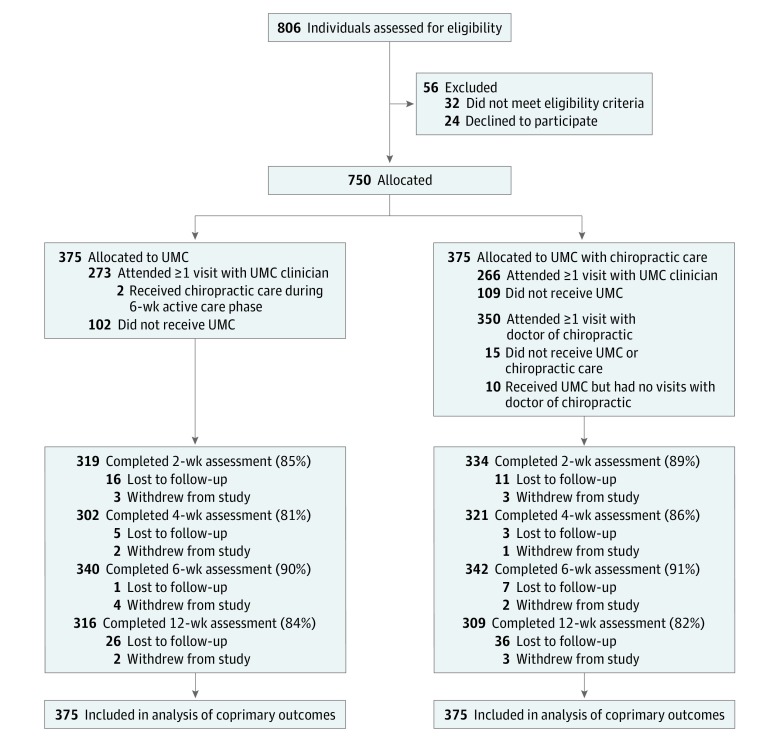
Trial Flow Diagram UMC indicates usual medical care.

A total of 806 patients were screened between September 28, 2012, and November 20, 2015, with 750 (250 at each of the 3 sites) allocated to receive UMC with chiropractic care (375 participants) or UMC alone (375 participants) (Figure 1). Data collection was completed on February 13, 2016, and data analysis was conducted from March 7, 2016, through April 30, 2016. Demographic characteristics, in particular age, race, and LBP chronicity, differed between sites (Table 1). Overall, the mean (SD) participant age was 30.9 (8.7) years, 175 participants (23.3%) were female, and 243 participants (32.4%) were nonwhite. Forty-three participants (5.7%) reported current use of narcotic analgesics and 398 (53.1%) took nonsteroidal anti-inflammatory drugs for back pain. Among all participants, 439 (58.5%) had never been treated with chiropractic care.

**Table 1.** Baseline Characteristics of 750 Participants

Characteristic	No. (%)							
	Walter Reed^a^		Pensacola^a^		San Diego^a^		Overall	
	UMC Alone (n = 125)	UMC + CC (n = 125)	UMC Alone (n = 125)	UMC + CC (n = 125)	UMC Alone (n = 125)	UMC + CC (n = 125)	UMC Alone (n = 375)	UMC + CC (n = 375)
Age, mean (SD), y	34.4 (8.4)	34.7 (8.6)	25.5 (7.9)	25.7 (7.5)	32.4 (7.4)	32.4 (7.5)	30.8 (8.8)	30.9 (8.7)
Male	86 (68.8)	85 (68.0)	106 (84.8)	107 (85.6)	95 (76.0)	96 (76.8)	287 (76.5)	288 (76.8)
Hispanic or Latino	16 (12.8)	9 (7.2)	29 (23.2)	12 (9.6)	21 (16.8)	31 (24.8)	66 (17.6)	52 (13.9)
Race								
Asian	6 (4.8)	3 (2.4)	3 (2.4)	1 (0.8)	11 (8.8)	6 (4.8)	20 (5.3)	10 (2.7)
Black or African American	41 (32.8)	42 (33.6)	17 (13.6)	15 (12.0)	14 (11.2)	20 (16.0)	72 (19.2)	77 (20.5)
White	62 (49.6)	62 (49.6)	102 (81.6)	106 (84.8)	88 (70.4)	87 (69.6)	252 (67.2)	255 (68.0)
Other or unspecified	16 (12.8)	18 (14.4)	2 (1.6)	7 (5.6)	11 (8.8)	17 (13.6)	31 (8.3)	33 (8.8)
LBP duration, mo								
<1	42 (33.6)	43 (34.4)	81 (64.8)	80 (64.0)	21 (16.8)	20 (16.0)	144 (38.4)	143 (38.1)
1-3	14 (11.2)	14 (11.2)	17 (13.6)	17 (13.6)	9 (7.2)	8 (6.4)	40 (10.7)	39 (10.4)
>3	69 (55.2)	68 (54.4)	27 (21.6)	28 (22.4)	95 (76.0)	97 (77.6)	191 (50.9)	193 (51.5)
BMI, mean (SD)	27.7 (3.9)	27.2 (3.7)	26.0 (3.3)	25.6 (3.5)	26.6 (3.5)	26.8 (3.6)	26.8 (3.6)	26.5 (3.7)
Current smoker	9 (7.2)	5 (4.0)	28 (22.4)	15 (12.0)	27 (21.6)	17 (13.6)	64 (17.1)	37 (9.9)
Used NSAIDs for LBP in past wk	82 (65.6)	75 (60.0)	59 (47.2)	58 (46.4)	54 (43.2)	70 (56.0)	195 (52.0)	203 (54.1)
Used narcotic analgesics for LBP in past wk	9 (7.2)	12 (9.6)	1 (0.8)	4 (3.2)	11 (8.8)	6 (4.8)	21 (5.6)	22 (5.9)
Never been to a doctor of chiropractic	70 (56.5)	64 (51.2)	92 (73.6)	84 (67.7)	68 (54.4)	61 (48.8)	230 (61.3)	209 (55.7)
NRS score for average LBP during past wk, mean (SD)^b^	4.3 (1.7)	4.4 (1.8)	4.7 (2.0)	4.4 (2.2)	4.8 (2.1)	5.0 (2.0)	4.6 (2.0)	4.6 (2.0)
RMDQ score for disability, mean (SD)^c^	10.3 (5.4)	9.7 (5.5)	11.0 (5.2)	10.5 (5.9)	8.9 (5.7)	9.3 (5.2)	10.1 (5.5)	9.8 (5.6)
Score of bothersomeness of LBP, mean (SD)^d^	3.6 (1.0)	3.5 (1.0)	3.8 (0.9)	3.7 (1.0)	3.5 (1.0)	3.5 (0.9)	3.7 (1.0)	3.6 (1.0)
NRS score for worst LBP in past 24 h, mean (SD)^b^	5.6 (2.4)	5.7 (2.0)	6.6 (1.9)	6.2 (2.1)	5.4 (2.4)	5.4 (2.1)	5.9 (2.3)	5.8 (2.1)
Expectation of UMC + CC, mean (SD)^e^	8.2 (1.8)	8.4 (1.6)	8.1 (1.9)	8.3 (1.9)	8.6 (1.8)	8.5 (1.9)	8.3 (1.9)	8.4 (1.8)
Expectation of UMC alone, mean (SD)^e^	5.3 (2.4)	4.9 (2.5)	5.3 (2.3)	5.4 (2.5)	4.5 (2.8)	5.2 (3.0)	5.0 (2.5)	5.2 (2.7)

Abbreviations: BMI, body mass index (calculated as weight in kilograms divided by height in meters squared); CC, chiropractic care; LBP, low back pain; NRS, Numerical Rating Scale; NSAIDs, nonsteroidal anti-inflammatory drugs; RMDQ, Roland Morris Disability Questionnaire; UMC, usual medical care.

^a^ Walter Reed indicates Walter Reed National Military Medical Center, Bethesda, Maryland; Pensacola indicates Naval Hospital Pensacola, Pensacola, Florida; and San Diego indicates Naval Medical Center San Diego, San Diego, California.

^b^ Possible scores range from 0 (no LBP) to 10 (worst possible LBP).

^c^ Possible scores range from 0 to 24, with higher scores indicating greater disability.

^d^ Possible scores range from 1 (not at all bothersome) to 5 (extremely bothersome).

^e^ Indicates participant’s expectation of helpfulness of treatment for LBP, measured on a scale of 0 (not helpful at all) to 10 (extremely helpful).

There were 102 participants in the UMC group who did not visit a UMC clinician: 6 at Walter Reed, 2 at Pensacola, and 94 at San Diego. Of the 273 patients who had at least 1 visit to a UMC clinician, the mean (SD) number of visits was 2.6 (2.3) at Walter Reed, 2.3 (2.3) at Pensacola, and 2.7 (2.5) at San Diego. There were 109 participants in the group receiving UMC with chiropractic care who did not visit a UMC clinician: 11 at Walter Reed and 98 at San Diego. Of the 266 patients who had at least 1 visit to a UMC clinician, the mean (SD) number of visits was 2.6 (3.1) at Walter Reed, 1.6 (1.6) at Pensacola, and 3.5 (3.0) at San Diego. Of the 350 participants who had at least 1 chiropractic visit, the mean (SD) number of visits was 4.7 (2.5) at Walter Reed, 5.4 (2.6) at Pensacola, and 2.3 (1.4) at San Diego.

### Primary Outcomes

**Figure 2.**
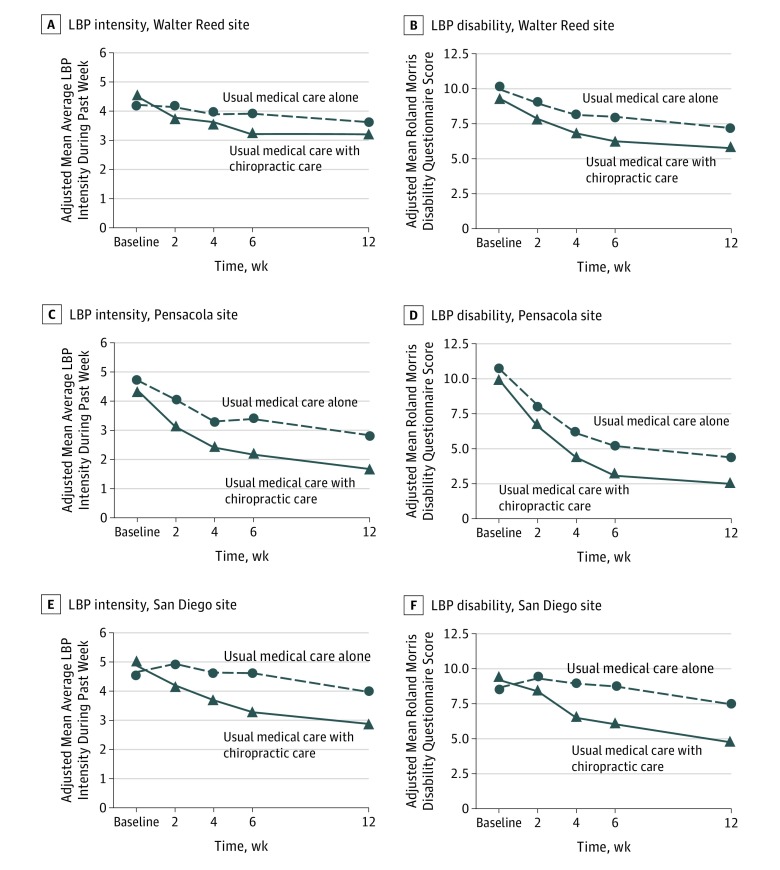
Adjusted Mean Low Back Pain (LBP) Intensity and Disability Over Time by Site Estimated from mixed-effects models using all observed data, an unstructured covariance, and terms in the model for time (as a categorical variable), site, and site × group, time × group, and site × time × group interactions, adjusted for sex, age, pain duration, and worst pain during the past 24 hours. Low back pain intensity during the prior week was assessed by the Numerical Rating Scale (scores ranging from 0 [no LBP] to 10 [worst possible LBP]); LBP-related functional disability was assessed by the Roland Morris Disability Questionnaire (scores ranging from 0-24, with higher scores indicating greater disability). Walter Reed indicates Walter Reed National Military Medical Center, Bethesda, Maryland; Pensacola indicates Naval Hospital Pensacola, Pensacola, Florida; and San Diego indicates Naval Medical Center San Diego, San Diego, California.

We found significant site × time × group interactions in all models. Adjusted mean differences between groups overall were consistently in favor of UMC with chiropractic care compared with UMC alone for the coprimary outcome variables of LBP intensity (mean difference, −1.1; 95% CI, −1.4 to −0.7) and disability (mean difference, −2.2; 95% CI, −3.1 to −1.2) at week 6 (Table 2) as well as at all 3 sites (Figure 2). Findings at week 12 were similar, but with a slightly smaller magnitude of difference (Table 2). Results of the sensitivity analysis showed effects in the same direction with similar magnitudes and statistical significance under both missing-at-random and missing-not-at-random approaches for plausible shift parameters of the coprimary outcome variables at all sites. The possible exception to this was week 6 RMDQ scores at Pensacola, for which a tipping point of 2.33 (mean difference, −1.44; 95% CI, −2.91 to 0.03) was found; however, we considered this to be an unlikely mean difference between participants with and without missing data. Relative risks (RRs) in the responder analysis were statistically significantly in favor of greater benefit for the group receiving UMC with chiropractic care compared with the group receiving UMC alone overall at week 6 (LBP intensity: RR = 1.43; 95% CI, 1.23 to 1.68; disability: RR = 1.35; 95% CI, 1.16 to 1.56) and week 12 (LBP intensity: RR = 1.43; 95% CI, 1.23 to 1.68; disability: RR = 1.26; 95% CI, 1.11 to 1.43) and at San Diego at weeks 6 and 12 but not at Walter Reed at weeks 6 or 12 or at Pensacola at week 12 (Table 3).

**Table 2.** Adjusted Within-Group Means and Between-Group Differences of Means in LBP and Disability^a^

Outcomes	Walter Reed^b^			Pensacola^b^			San Diego^b^			Overall		
	Within-Group Mean (95% CI)		UMC + CC vs UMC Alone, Between-Group Difference of Means (95% CI)	Within-Group Mean (95% CI)		UMC + CC vs UMC Alone, Between-Group Difference of Means (95% CI)	Within-Group Mean (95% CI)		UMC + CC vs UMC Alone, Between-Group Difference of Means (95% CI)	Within-Group Mean (95% CI)		UMC + CC vs UMC Alone, Between-Group Difference of Means (95% CI)
	UMC Alone	UMC + CC		UMC Alone	UMC + CC		UMC Alone	UMC + CC		UMC Alone	UMC + CC	
**Primary Outcomes**												
NRS score for average LBP during past wk^c^												
6 wk	3.9 (3.5 to 4.3)	3.2 (2.8 to 3.6)	−0.7 (−1.3 to −0.1)	3.4 (2.9 to 3.9)	2.2 (1.7 to 2.6)	−1.2 (−1.8 to −0.6)	4.6 (4.2 to 5.1)	3.3 (2.9 to 3.7)	−1.3 (−1.9 to −0.8)	4.0 (3.7 to 4.2)	2.9 (2.6 to 3.2)	−1.1 (−1.4 to −0.7)
12 wk	3.6 (3.2 to 4.1)	3.2 (2.8 to 3.7)	−0.4 (−1.0 to 0.2)	2.8 (2.3 to 3.3)	1.7 (1.2 to 2.2)	−1.1 (−1.8 to −0.5)	4.0 (3.5 to 4.5)	2.9 (2.5 to 3.4)	−1.1 (−1.7 to −0.5)	3.5 (3.2 to 3.8)	2.6 (2.3 to 2.9)	−0.9 (−1.2 to −0.5)
RMDQ score for disability^d^												
6 wk	8.0 (6.8 to 9.2)	6.2 (5.0 to 7.4)	−1.8 (−3.4 to −0.2)	5.2 (3.9 to 6.5)	3.1 (1.8 to 4.4)	−2.1 (−3.8 to −0.4)	8.8 (7.6 to 10.1)	6.1 (4.9 to 7.3)	−2.7 (−4.3 to −1.1)	7.3 (6.5 to 8.1)	5.2 (4.4 to 5.9)	−2.2 (−3.1 to −1.2)
12 wk	7.3 (6.0 to 8.5)	5.8 (4.5 to 7.0)	−1.5 (−3.2 to 0.2)	4.4 (3.0 to 5.8)	2.5 (1.1 to 3.8)	−1.9 (−3.7 to −0.2)	7.6 (6.3 to 8.8)	4.8 (3.6 to 6.1)	−2.7 (−4.4 to −1.1)	6.4 (5.6 to 7.2)	4.4 (3.6 to 5.2)	−2.0 (−3.0 to −1.0)
**Secondary Outcomes**												
Score of bothersomeness of LBP^e^												
6 wk	3.0 (2.8 to 3.2)	2.8 (2.6 to 3.0)	−0.2 (−0.5 to 0.1)	2.7 (2.4 to 2.9)	2.1 (1.9 to 2.3)	−0.5 (−0.8 to −0.2)	3.2 (3.0 to 3.4)	2.8 (2.6 to 2.9)	−0.5 (−0.7 to −0.2)	3.0 (2.8 to 3.1)	2.6 (2.4 to 2.7)	−0.4 (−0.6 to −0.2)
12 wk	2.9 (2.7 to 3.1)	2.8 (2.6 to 3.0)	−0.2 (−0.5 to 0.1)	2.4 (2.1 to 2.6)	1.8 (1.6 to 2.1)	−0.5 (−0.8 to −0.2)	2.9 (2.6 to 3.1)	2.4 (2.2 to 2.6)	−0.4 (−0.7 to −0.1)	2.7 (2.6 to 2.9)	2.3 (2.2 to 2.5)	−0.4 (−0.6 to −0.2)
NRS score for worst LBP in past 24 h^c^												
6 wk	4.5 (4.1 to 5.0)	3.9 (3.4 to 4.3)	−0.7 (−1.3 to −0.02)	3.4 (2.9 to 3.9)	2.1 (1.6 to 2.6)	−1.3 (−2.0 to −0.7)	5.3 (4.8 to 5.7)	3.6 (3.2 to 4.1)	−1.6 (−2.3 to −1.0)	4.4 (4.1 to 4.7)	3.2 (2.9 to 3.5)	−1.2 (−1.6 to −0.8)
12 wk	4.4 (3.9 to 4.9)	3.7 (3.2 to 4.2)	−0.8 (−1.5 to −0.1)	3.0 (2.4 to 3.5)	1.4 (0.9 to 2.0)	−1.5 (−2.3 to −0.8)	4.3 (3.8 to 4.8)	3.2 (2.7 to 3.7)	−1.2 (−1.9 to −0.5)	3.9 (3.5 to 4.2)	2.7 (2.4 to 3.1)	−1.1 (−1.6 to −0.7)

Abbreviations: CC, chiropractic care; LBP, low back pain; NRS, Numerical Rating Scale; RMDQ, Roland Morris Disability Questionnaire; UMC, usual medical care.

^a^ Estimated from mixed-effects models using all observed data, an unstructured covariance, and terms in the model for time (as a categorical variable), site, and site × group, time × group, and site × time × group interactions, adjusted for sex, age, pain duration, and worst pain during the past 24 hours. The Bonferroni method was used to control for analyzing the coprimary outcome variables. Mean between-group differences may be off by 0.1 due to rounding.

^b^ Walter Reed indicates Walter Reed National Military Medical Center, Bethesda, Maryland; Pensacola indicates Naval Hospital Pensacola, Pensacola, Florida; and San Diego indicates Naval Medical Center San Diego, San Diego, California.

^c^ Possible scores range from 0 (no LBP) to 10 (worst possible LBP).

^d^ Possible scores range from 0 to 24, with higher scores indicating greater disability.

^e^ Possible scores range from 1 (not at all bothersome) to 5 (extremely bothersome).

**Table 3.** Within-Group Percentage of Responders and Between-Group RRs for Primary Outcomes^a^

Scale	Walter Reed^b^			Pensacola^b^			San Diego^b^			Overall		
	Within-Group Responders, %		UMC + CC vs UMC Alone, RR (95% CI)	Within-Group Responders, %		UMC + CC vs UMC Alone, RR (95% CI)	Within-Group Responders, %		UMC + CC vs UMC Alone, RR (95% CI)	Within-Group Responders, %		UMC + CC vs UMC Alone, RR (95% CI)
	UMC Alone	UMC + CC		UMC Alone	UMC + CC		UMC Alone	UMC + CC		UMC Alone	UMC + CC	
NRS for average LBP during past wk												
6 wk	38.0 (29.9-48.3)	49.1 (40.5-59.5)	1.29 (0.96-1.73)	46.1 (37.4-56.8)	69.1 (59.3-80.5)	1.50 (1.20-1.88)	19.7 (13.2-29.3)	56.0 (46.0-68.2)	2.85 (1.85-4.38)	32.5 (27.3-38.8)	57.5 (51.2-64.6)	1.77 (1.46-2.14)
12 wk	37.1 (28.9-47.5)	48.5 (39.9-59.0)	1.31 (0.97-1.78)	52.8 (43.4-64.1)	73.4 (63.5-84.8)	1.39 (1.14-1.70)	39.2 (30.1-51.1)	63.5 (53.1-75.9)	1.62 (1.20-2.18)	42.5 (36.7-49.2)	60.9 (54.5-68.1)	1.43 (1.23-1.68)
RMDQ for disability												
6 wk	43.8 (35.4-54.1)	53.7 (45.0-64.1)	1.23 (0.94-1.60)	63.0 (53.9-73.6)	76.6 (67.5-86.9)	1.22 (1.03-1.43)	36.6 (28.0-47.7)	59.7 (49.9-71.4)	1.63 (1.20-2.22)	46.6 (40.9-53.0)	62.6 (56.6-69.3)	1.35 (1.16-1.56)
12 wk	50.4 (41.6-61.0)	59.2 (50.2-69.9)	1.18 (0.92-1.50)	71.7 (62.4-82.4)	79.5 (70.5-89.7)	1.11 (0.96-1.28)	47.2 (37.6-59.3)	72.4 (62.1-84.4)	1.53 (1.18-1.99)	55.5 (49.4-62.2)	69.9 (63.6-76.7)	1.26 (1.11-1.43)

Abbreviations: CC, chiropractic care; LBP, low back pain; NRS, Numerical Rating Scale; RMDQ, Roland Morris Disability Questionnaire; RR, relative risk; UMC, usual medical care.

^a^ Responder is defined as at least 30% improvement from baseline. Estimated from modified Poisson regression generalized estimating equation models with terms in the model for time (as a categorical variable), group, site, and site × group, time × group, and site × time × group interactions, adjusted for sex, age, pain duration, and worst pain during the past 24 hours.

^b^ Walter Reed indicates Walter Reed National Military Medical Center, Bethesda, Maryland; Pensacola indicates Naval Hospital Pensacola, Pensacola, Florida; and San Diego indicates Naval Medical Center San Diego, San Diego, California.

### Secondary Outcomes

Overall at weeks 6 and 12, participants receiving UMC with chiropractic care, compared with UMC alone, reported significantly lower mean worst LBP intensity within the past 24 hours (week 6: mean difference, −1.2; 95% CI, −1.6 to −0.8; week 12: mean difference, −1.1; 95% CI, −1.6 to −0.7) and symptom bothersomeness (week 6: mean difference, −0.4; 95% CI, −0.6 to −0.2; week 12: mean difference, −0.4; 95% CI, −0.6 to −0.2); the differences at each site also were statistically significant, with the exception of bothersomeness at Walter Reed (Table 2). Participants receiving UMC with chiropractic care had significantly better global perceived improvement at 6 weeks at all sites (overall: odds ratio [OR] = 0.18; 95% CI, 0.13 to 0.25; Walter Reed: OR = 0.26; 95% CI, 0.16 to 0.42; Pensacola: OR = 0.18; 95% CI, 0.10 to 0.33; San Diego: OR = 0.13; 95% CI, 0.08 to 0.21). Similarly, those receiving UMC with chiropractic care had significantly greater mean satisfaction with care at 6 weeks at all sites (overall: mean difference, 2.5; 95% CI, 2.1 to 2.8; Walter Reed: mean difference, 2.0; 95% CI, 1.4 to 2.6; Pensacola: mean difference, 2.3; 95% CI, 1.6 to 3.0; San Diego: mean difference, 3.1; 95% CI, 2.5 to 3.7). Overall, participants allocated to receive UMC with chiropractic care self-reported significantly less pain medication use than those receiving UMC alone at week 6 (OR = 0.73; 95% CI, 0.54 to 0.97) and week 12 (OR = 0.76; 95% CI, 0.58 to 1.00), but not at any of the individual sites.

### Additional Therapeutic Procedures and UMC

Chiropractic care consisted of several therapeutic procedures in addition to spinal manipulation (eTable 2 in Supplement 2). Use of these therapies varied substantially by site. Participants at Walter Reed were most likely to receive multiple ancillary therapies across the range of options, while most in San Diego received therapeutic exercise for strength and flexibility. Usual medical care included physical therapy referrals and prescription medication, with less variation across sites than that for chiropractic care (eTable 3 in Supplement 2).

### Adverse Events

Three unrelated serious adverse events were reported. There were 62 adverse effects reported throughout the 6-week active care phase: 38 at Walter Reed, 16 at Pensacola, and 8 at San Diego. Of the 19 adverse effects reported by participants receiving UMC alone, 3 were due to prescribed medications, 4 were related to epidural injections, and 12 consisted of muscle or joint stiffness attributed to physical therapy or self-care recommendations. Of the 43 adverse effects reported by participants receiving UMC with chiropractic care, 38 were described as muscle or joint stiffness attributed to chiropractic care (37 events) or physical therapy (1 event), 1 was reported as indistinct symptoms following an epidural injection, 3 were described as pain, tingling, or sensitivity in an extremity without reference to a specific treatment, and 1 was a lower-extremity burning sensation for 20 minutes following spinal manipulative therapy.

## Discussion

The changes in patient-reported pain intensity and disability as well as satisfaction with care and low risk of harms favoring UMC with chiropractic care found in this pragmatic clinical trial are consistent with the existing literature on spinal manipulative therapy in both military^23^ and civilian^20,36,37,38^ populations. The magnitude of mean between-group differences for both pain (NRS) and disability (RMDQ) are consistent with a moderate magnitude of effect as classified by the American College of Physicians and American Pain Society guidelines.^34,37^

This trial has several important strengths. The first is its pragmatic design. The advantages and disadvantages of a pragmatic clinical trial when evaluating complex treatment approaches have been well argued.^39^ These issues were considered by the study team prior to protocol development within the context that (1) placebo-controlled trials of spinal manipulation exist,^40,41^ (2) chiropractic care is already integrated into more than half of military treatment facilities across the United States,^42,43^ and (3) spinal manipulation or chiropractic care is recommended as a first line of treatment for pain management in military and civilian guidelines.^21,22^ Furthermore, a pragmatic approach provides the opportunity to better understand the effect of chiropractic care as it is currently integrated and delivered within military health care settings as part of a multidisciplinary approach.^44^ This is important given the lack of consensus regarding optimal treatment regimens for LBP in general as well as chiropractic care specifically.

Second, most trials of chiropractic care for LBP have compared it with other monotherapies or usual care. This study focused on understanding the effect of adding chiropractic care to UMC vs UMC alone, which is consistent with how military health care is provided. Results from this pragmatic design can directly inform military health care practices and shed light on the potential effect of chiropractic care in other integrated health care delivery settings, such as patient-centered medical homes.

Third, this trial included a more diverse sample in terms of race, ethnicity, and age than found in prior studies of either chiropractic or spinal manipulation.^25^

Fourth, our sample of 750 participants is the largest trial, to our knowledge, evaluating UMC with chiropractic care vs UMC alone, allowing us to look both across and within 3 military treatment facilities using standardized outcome measures. By addressing several important limitations, such as small sample sizes, real-world applicability, and inconsistencies in outcome measures identified in previous systematic reviews,^20,36^ our findings further support existing guidelines that recommend nonpharmacological treatments as a first line of treatment for LBP.^22,37^ This is a critically important issue as the US health care delivery system struggles to adequately address the challenges of managing LBP and the opioid epidemic.^15,16^

### Limitations

Although our study findings strengthen the scientific evidence for the use of chiropractic care in patients with LBP, study limitations leave a number of questions unanswered. Some are inherent to the nonspecific nature of LBP, the treatment approach used, and trial design. As is true with all studies of LBP of musculoskeletal origin, the specific diagnosis was difficult to determine or confirm, contributing toward patient heterogeneity that is not ideally characterized and is thus difficult to account for in the analysis. This problem was potentially exacerbated by the use of broad inclusion criteria, which are characteristic of pragmatic designs to increase the generalizability of study findings. However, it is difficult to specify the extent to which this heterogeneity contributed to patient outcomes within the context of this trial. Similarly, the use of hands-on, multimodal interventions commonly delivered by doctors of chiropractic makes it difficult to mask participants to treatment group or to control for the fact that those with chiropractic care received more time and attention from clinicians. Furthermore, cross-contamination of treatment approaches among and between clinicians makes it difficult to specifically identify which component(s) of both UMC and chiropractic interventions were associated with the beneficial effects found in this trial.

Additional limitations specific to the ways in which this particular trial was designed also exist. Participant visit numbers varied across sites for both UMC and chiropractic care. Although those enrolled across all 3 sites were encouraged in a similar manner to seek care based on their treatment group allocation, a larger number of participants in San Diego chose not to access UMC, a discrepancy that may explain why fewer participants at San Diego who received UMC alone were classified as respondents at 6 weeks. We attribute these differences in adherence to variations in trial recruitment strategies. At Walter Reed and Pensacola, most participants were recruited from primary care clinics by physicians, so they had already chosen to seek medical care for their LBP. The majority of participants at San Diego were recruited using flyers and screened for eligibility by Independent Duty Corpsmen. We do not know whether these participants chose not to access UMC (1) for logistical reasons, (2) because UMC had not been beneficial when used previously, or (3) for some other reason that may have affected their LBP outcomes. These are important considerations for future research.

Differences in participant characteristics, treatments received, and outcomes across sites are also limitations. The use of standardized outcome measures and inclusion of 250 participants at each site addressed anticipated differences in patient and practice characteristics. However, this strategy was only partially successful. It is not surprising that Pensacola had the highest proportion of responders at 6 and 12 weeks for both primary outcomes, as participants here were younger and more likely to report acute LBP (<1 month). It is less clear why participants at San Diego, most likely to report chronic LBP (>3 months: >75%) and less likely to seek UMC, showed larger between-group differences in LBP disability at both end points even though they had approximately half the chiropractic visits found at the other 2 sites. Further work is needed to determine the effect of chiropractic visit numbers on outcomes.

Another challenge, unique to conducting research in the military, was following patients who are highly transient, especially in times of war. When this study was designed, the active-duty population of interest was likely to be deployed. Consequently, we chose to follow participants for 12 weeks and excluded those who were scheduled to leave the country within that period. As a result, an additional limitation of this study is the relatively short follow-up.

## Conclusions

Chiropractic care, when added to UMC, resulted in moderate short-term treatment benefits in both LBP intensity and disability, demonstrated a low risk of harms, and led to high patient satisfaction and perceived improvement in active-duty military personnel. This trial provides additional support for the inclusion of chiropractic care as a component of multidisciplinary health care for LBP, as currently recommended in existing guidelines.^21,22,37^ However, study limitations illustrate that further research is needed to understand longer-term outcomes as well as how patient heterogeneity and intervention variations affect patient responses.

## Notes

Supplement 1. **Trial Protocol**

Supplement 2. **eTable 1.** Primary and Secondary Outcomes **eTable 2.** Additional Therapeutic Procedures Delivered by Doctors of Chiropractic for Participants in the Usual Medical Care With Chiropractic Care Group **eTable 3.** Types of Usual Medical Care **eReferences**

## References

[1] Vos T, Flaxman AD, Naghavi M, Years lived with disability (YLDs) for 1160 sequelae of 289 diseases and injuries 1990-2010: a systematic analysis for the Global Burden of Disease Study 2010. Lancet. 2012;380(9859):-. 23245607 10.1016/S0140-6736(12)61729-2 PMC6350784

[2] US Bone and Joint Initiative The burden of musculoskeletal diseases in the United States. 2016 http://www.boneandjointburden.org/. Accessed February 17, 2018.

[3] Meucci RD, Fassa AG, Faria NM Prevalence of chronic low back pain: systematic review [published online October 20, 2015]. Rev Saude Publica. 2015;49:S0034-89102015000100408. doi:10.1590/S0034-8910.2015049005874 26487293 PMC4603263

[4] Gaskin DJ, Richard P The economic costs of pain in the United States. J Pain. 2012;13(8):715-724. 22607834 10.1016/j.jpain.2012.03.009

[5] Institute for Health Metrics and Evaluation The Global Burden of Disease: Generating Evidence, Guiding Policy. Seattle, WA: Institute for Health Metrics & Evaluation; 2013.

[6] Cohen SP, Gallagher RM, Davis SA, Griffith SR, Carragee EJ Spine-area pain in military personnel: a review of epidemiology, etiology, diagnosis, and treatment. Spine J. 2012;12(9):833-842. 22100208 10.1016/j.spinee.2011.10.010

[7] Clark LL, Hu Z Diagnoses of low back pain, active component, U.S. Armed Forces, 2010-2014. MSMR. 2015;22(12):8-11. 26726722

[8] Deyo RA, Mirza SK, Turner JA, Martin BI Overtreating chronic back pain: time to back off? J Am Board Fam Med. 2009;22(1):62-68. 19124635 10.3122/jabfm.2009.01.080102 PMC2729142

[9] Manchikanti L, Knezevic NN, Boswell MV, Kaye AD, Hirsch JA Epidural injections for lumbar radiculopathy and spinal stenosis: a comparative systematic review and meta-analysis. Pain Physician. 2016;19(3):E365-E410. 27008296

[10] Machado GC, Maher CG, Ferreira PH, Day RO, Pinheiro MB, Ferreira ML Non-steroidal anti-inflammatory drugs for spinal pain: a systematic review and meta-analysis. Ann Rheum Dis. 2017;76(7):1269-1278. 28153830 10.1136/annrheumdis-2016-210597

[11] Fineberg SJ, Nandyala SV, Kurd MF, Incidence and risk factors for postoperative ileus following anterior, posterior, and circumferential lumbar fusion. Spine J. 2014;14(8):1680-1685. 24184650 10.1016/j.spinee.2013.10.015

[12] Marquez-Lara A, Nandyala SV, Fineberg SJ, Singh K Cerebral vascular accidents after lumbar spine fusion. Spine (Phila Pa 1976). 2014;39(8):673-677. 24384658 10.1097/BRS.0000000000000197

[13] Martin BI, Mirza SK, Franklin GM, Lurie JD, MacKenzie TA, Deyo RA Hospital and surgeon variation in complications and repeat surgery following incident lumbar fusion for common degenerative diagnoses. Health Serv Res. 2013;48(1):1-25. 22716168 10.1111/j.1475-6773.2012.01434.x PMC3465627

[14] Bally M, Dendukuri N, Rich B, Risk of acute myocardial infarction with NSAIDs in real world use: Bayesian meta-analysis of individual patient data. BMJ. 2017;357:j1909. 28487435 10.1136/bmj.j1909 PMC5423546

[15] Dowell D, Haegerich TM, Chou R CDC guideline for prescribing opioids for chronic pain—United States, 2016. JAMA. 2016;315(15):1624-1645. 26977696 10.1001/jama.2016.1464 PMC6390846

[16] Brummett CM, Waljee JF, Goesling J, New persistent opioid use after minor and major surgical procedures in US adults. JAMA Surg. 2017;152(6):e170504. 28403427 10.1001/jamasurg.2017.0504 PMC7050825

[17] Amorin-Woods LG, Parkin-Smith GF Clinical decision-making to facilitate appropriate patient management in chiropractic practice: “the 3-questions model.” Chiropr Man Therap. 2012;20(1):6. 22417567 10.1186/2045-709X-20-6 PMC3316132

[18] American Physical Therapy Association Position on thrust joint manipulation provided by physical therapists. February 2009 http://www.apta.org/uploadedFiles/APTAorg/Advocacy/State/Issues/Manipulation/WhitePaperManipulation.pdf. Accessed February 20, 2009.

[19] Weeks WB, Goertz CM, Meeker WC, Marchiori DM Public perceptions of doctors of chiropractic: results of a national survey and examination of variation according to respondents’ likelihood to use chiropractic, experience with chiropractic, and chiropractic supply in local health care markets. J Manipulative Physiol Ther. 2015;38(8):533-544. 26362263 10.1016/j.jmpt.2015.08.001

[20] Nahin RL, Boineau R, Khalsa PS, Stussman BJ, Weber WJ Evidence-based evaluation of complementary health approaches for pain management in the United States. Mayo Clin Proc. 2016;91(9):1292-1306. 27594189 10.1016/j.mayocp.2016.06.007 PMC5032142

[21] Office of the Army Surgeon General Pain Management Task Force, Final Report, May 2010: Providing a Standardized DoD and VHA Vision and Approach to Pain Management to Optimize the Care for Warriors and Their Families. Washington, DC: Office of the Army Surgeon General; 2010.

[22] Qaseem A, Wilt TJ, McLean RM, Forciea MA; Clinical Guidelines Committee of the American College of Physicians Noninvasive treatments for acute, subacute, and chronic low back pain: a clinical practice guideline from the American College of Physicians. Ann Intern Med. 2017;166(7):514-530. 28192789 10.7326/M16-2367

[23] Goertz CM, Long CR, Hondras MA, Adding chiropractic manipulative therapy to standard medical care for patients with acute low back pain: results of a pragmatic randomized comparative effectiveness study. Spine (Phila Pa 1976). 2013;38(8):627-634. 23060056 10.1097/BRS.0b013e31827733e7

[24] TRICARE Find a military hospital or clinic. https://tricare.mil/mtf#zip=&radius=40&facility=&country=&state=&region=undefined&specialty=23&service=&pageNo=0&pageCount=5&view=map&fids=188,189,68,57,130. Accessed January 23, 2018.

[25] Goertz CM, Pohlman KA, Vining RD, Brantingham JW, Long CR Patient-centered outcomes of high-velocity, low-amplitude spinal manipulation for low back pain: a systematic review. J Electromyogr Kinesiol. 2012;22(5):670-691. 22534288 10.1016/j.jelekin.2012.03.006

[26] Goertz CM, Long CR, Vining RD, Assessment of chiropractic treatment for active duty, U.S. military personnel with low back pain: study protocol for a randomized controlled trial. Trials. 2016;17:70. 26857706 10.1186/s13063-016-1193-8 PMC4746780

[27] Childs JD, Piva SR, Fritz JM Responsiveness of the numeric pain rating scale in patients with low back pain. Spine (Phila Pa 1976). 2005;30(11):1331-1334. 15928561 10.1097/01.brs.0000164099.92112.29

[28] van der Roer N, Ostelo RWJG, Bekkering GE, van Tulder MW, de Vet HCW Minimal clinically important change for pain intensity, functional status, and general health status in patients with nonspecific low back pain. Spine (Phila Pa 1976). 2006;31(5):578-582. 16508555 10.1097/01.brs.0000201293.57439.47

[29] Jensen MP, Karoly P Self-report scales and procedures for assessing pain in adults In: Turk DC, Melzack R, eds. Handbook of Pain Assessment. 2nd ed New York, NY: Guilford Press; 2001:15-34.

[30] Riddle DL, Stratford PW, Binkley JM Sensitivity to change of the Roland-Morris Back Pain Questionnaire: part 2. Phys Ther. 1998;78(11):1197-1207. 9806624 10.1093/ptj/78.11.1197

[31] Dunn KM, Croft PR Classification of low back pain in primary care: using “bothersomeness” to identify the most severe cases. Spine (Phila Pa 1976). 2005;30(16):1887-1892. 16103861 10.1097/01.brs.0000173900.46863.02

[32] Dworkin RH, Turk DC, Farrar JT, ; IMMPACT Core outcome measures for chronic pain clinical trials: IMMPACT recommendations. Pain. 2005;113(1-2):9-19. 15621359 10.1016/j.pain.2004.09.012

[33] Kamper SJ, Ostelo RW, Knol DL, Maher CG, de Vet HC, Hancock MJ Global Perceived Effect scales provided reliable assessments of health transition in people with musculoskeletal disorders, but ratings are strongly influenced by current status. J Clin Epidemiol. 2010;63(7):760-766, e1. 20056385 10.1016/j.jclinepi.2009.09.009

[34] Chou R, Qaseem A, Snow V, ; Clinical Efficacy Assessment Subcommittee of the American College of Physicians; American College of Physicians; American Pain Society Low Back Pain Guidelines Panel Diagnosis and treatment of low back pain: a joint clinical practice guideline from the American College of Physicians and the American Pain Society. Ann Intern Med. 2007;147(7):478-491. 17909209 10.7326/0003-4819-147-7-200710020-00006

[35] Bombardier C, Hayden J, Beaton DE Minimal clinically important difference: low back pain: outcome measures. J Rheumatol. 2001;28(2):431-438. 11246692

[36] Chou R, Deyo R, Friedly J, Noninvasive Treatments for Low Back Pain. Rockville, MD: Agency for Healthcare Research & Quality; 2016. 26985522

[37] Chou R, Deyo R, Friedly J, Nonpharmacologic therapies for low back pain: a systematic review for an American College of Physicians clinical practice guideline. Ann Intern Med. 2017;166(7):493-505. 28192793 10.7326/M16-2459

[38] Paige NM, Miake-Lye IM, Booth MS, Association of spinal manipulative therapy with clinical benefit and harm for acute low back pain: systematic review and meta-analysis. JAMA. 2017;317(14):1451-1460. 28399251 10.1001/jama.2017.3086 PMC5470352

[39] Schwartz D, Lellouch J Explanatory and pragmatic attitudes in therapeutical trials. J Clin Epidemiol. 2009;62(5):499-505. 19348976 10.1016/j.jclinepi.2009.01.012

[40] von Heymann WJ, Schloemer P, Timm J, Muehlbauer B Spinal high-velocity low amplitude manipulation in acute nonspecific low back pain: a double-blinded randomized controlled trial in comparison with diclofenac and placebo. Spine (Phila Pa 1976). 2013;38(7):540-548. 23026869 10.1097/BRS.0b013e318275d09c

[41] Haas M, Vavrek D, Peterson D, Polissar N, Neradilek MB Dose-response and efficacy of spinal manipulation for care of chronic low back pain: a randomized controlled trial. Spine J. 2014;14(7):1106-1116. 24139233 10.1016/j.spinee.2013.07.468 PMC3989479

[42] Lisi AJ, Goertz C, Lawrence DJ, Satyanarayana P Characteristics of Veterans Health Administration chiropractors and chiropractic clinics. J Rehabil Res Dev. 2009;46(8):997-1002. 20157856 10.1682/jrrd.2009.01.0002

[43] Green BN, Johnson CD, Lisi AJ, Tucker J Chiropractic practice in military and veterans health care: the state of the literature. J Can Chiropr Assoc. 2009;53(3):194-204. 19714234 PMC2732257

[44] Lurie JD, Morgan TS Pros and cons of pragmatic clinical trials. J Comp Eff Res. 2013;2(1):53-58. 24236521 10.2217/cer.12.74

