# Supplementary material for: Effect of Usual Medical Care Plus Chiropractic Care vs Usual Medical Care Alone on Pain and Disability Among US Service Members With Low Back Pain: A Comparative Effectiveness Clinical Trial
Source: JAMA Netw Open. 2018 May 18;1(1):e180105. doi: 10.1001/jamanetworkopen.2018.0105 (PMC6324527; doi:10.1001/jamanetworkopen.2018.0105)
Supplement: Supplement 1. — Trial Protocol [file jamanetwopen-1-e180105-s001.pdf]

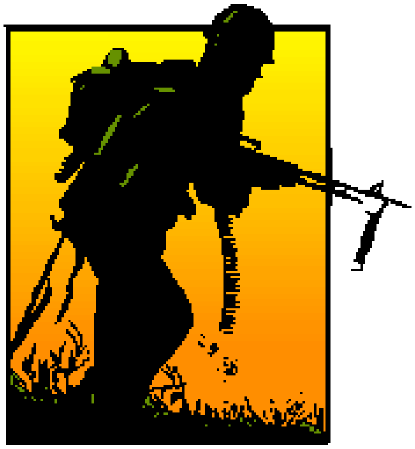

Study Protocol

Date: March 2014

Version: 13

Project Title: Assessment of Chiropractic Treatment for Low Back Pain and Smoking Cessation in Military Active Duty Personnel

Grant Title: Defense Health Program (DHP) Chiropractic Clinical Trial Award (W81XWH-11-2-0107)

Co-Principal Investigators:

Ian D Coulter, PhD
RAND Corp/Samueli Chair in Integrative Medicine

Christine Goertz, DC, PhD
Palmer College of Chiropractic/Vice Chancellor for Research and Health Policy

Joan Walter, JD, PA
Samueli Institute/Vice President, Military Medical Research Programs

CONFIDENTIALITY STATEMENT

The information contained in this document, especially unpublished data, is the property of Palmer College of Chiropractic and is therefore provided to you in confidence as an investigator, potential investigator, or consultant, for review by you, your staff, and an applicable Institutional Review Board/Independent Ethics Committee. It is understood that this information will not be disclosed to others without written authorization from a Co-Principal Investigator listed above.

Revisions:

4/14/2011	Corrected the grant contract number.
06/02/2011-<br>Version 4.1	Updated the Tobacco (Smoking) Cessation protocol.
06/14/2011-<br>Version 4.2	Updated 5.6 Reporting of Adverse Events with more exact details of the reporting process.
08/05/2011-<br>Version 5	Added the reference list.<br>Updated the Data Collection (added PROMIS-29; removed SF(RAND)-36).<br>Removed minimization on tobacco use.<br>Added outline for tobacco cessation training webinar.<br>Updated the Timeline (Table 2).
08/30/2011-<br>Version 6	Updated the Qualitative Analysis and Quality Assurance section.
012/01/2011-<br>Version 7	Added e-mail and text language (pg 20).<br>Added more specific on back-up network storage (pg 30).
03/08/2012<br>Version 8	Editorial changes (i.e. abbreviations spelled out; spelling errors corrected;<br>Ensured consistent use of CMT (Chiropractic Manipulative Therapy) and CMC (Conventional Medical Care)<br>Enlisted military personnel was changed to active duty military personnel in Specific Aim #1 (page 12)<br>National Navy Medical Center was changed to Walter Reed National Military Medical Center (WRNMMC)<br>Tobacco Cessation- Flow chart was added (Figure 11); description of study chiropractor's tobacco education survey is now described at the bottom of the 2nd paragraph.<br>Data Storage now states that the ICD/HIPAA documents will be kept in a locked filing cabinet.<br>Timeline (Table 2) has been updated.<br>Added:<br>Appendix B- Tobacco Cessation course handouts<br>Appendix B- Tobacco Cessation study chiropractor's survey<br>Appendix C- Blackboard Connect Protocol, including e-mail, voicemail, and text script reminders for assessments and appointments.
4/18/2012<br>Version 9	Added Appendix D- Data Collection Forms (Exam and DC Treatment)
7/23/2012<br>Version 10	Removed Communication Plan<br>Updated Section 5.2 Participant Visit Protocols, Section 5.5.2 Data Storage & Table 2<br>Added Appendix E- Participant Certification

8/29/2012<br>Version 11	Removed Phase II from the protocol<br>Qualified “altered mental capacity” exclusion criterion<br>Changed PCP to stand for primary care provider versus primary contact provider<br>Removed “Unable to speak English” as an exclusion criterion since all active duty personnel are required to speak English<br>Updated Exam form. Qualified the mental health question (#5) and updated the referral/further testing question for clarity (#6)<br>Updated Treatment form. Added relevant ICD codes were added and added another tobacco cessation option.<br>Updated Blackboard Connect Protocol (Appendix C): “Modifications to this protocol may occur based on individual participants needs or request.” Page 67<br>Updated Data Storage: “Treatment and Exam forms will be kept in the locked cabinet for a maximum of 7 days after all information has been entered into the CTCC database.” Page 32<br>Removed Rock Island Arsenal as a recruitment site. Page 18<br>Reduced the sample size to 750, since we are no longer recruiting the 100 participants for Rock Island.
6/26/2013<br>*Version* 12	Section 2.1<br>- Updated document to reflect the removal of Rock Island (changed 4 sites to 3 sites); Removed RI from Figure 2 and Figure 4.<br>- Changed ‘smoker’ to ‘tobacco-user’<br>Section 4.1. Added ‘Site PIs and PMs will have one-on-one monthly meetings with the PI and lead PM at Palmer.”<br>Section 5.1.1.1 – clarified low back pain episode<br>Section 5.1.2 – Eligibility criteria<br>- Added time frame for spinal fracture<br>- Added exclusion criterion of ‘spinal surgery within 12 weeks)<br>- Clarified exclusion of pregnancy by adding ‘planning to become pregnant within 8 weeks’<br>Section 5.2.1 – removed information sheet and changed photocopy of ICD to just a copy of the ICD will be given to the participant.<br>Section 5.2.2 Added in recruitment from an acute care provider; removed “It is at this point that patients complaining of LBP are typically either prescribed medication or referred to the base chiropractor.”; added independent duty corpsman as eligible to complete the baseline exam and give conventional medical care; and added that the exam form and baseline interview needs to be completed within 2 weeks of each other.<br>Section 5.2.3 Treatment Visits<br>- Clarified frequency/duration of chiropractic treatment based on what we have observed to date<br>- Clarified methods that Samueli/RAND will use when evaluating differences in participant treatment schedules<br>- Under “Tobacco Cessation’ section – changed method of re-training to allow for other training methods<br>- Changed frequency that chiropractor will complete a baseline tobacco cessation survery from ‘3 months after recruitment begins’ to ‘1 year after recruitment begins’.<br>Section 5.3 – clarified that NRS is on a 0-10 rating scale versus 0-11 which was inadvertent error<br>Section 5.3.2 – removed sentence pertaining to SF-36 as SF-36 was previously removed

	and replaced with PROMIS-29.<br>Section 5.4 – added clarification of reporting procedures for protocol deviations<br>Section 5.5.1 and Appendix D<br>- Removed use of Blackboard connect as method of reminding participants to complete study assessments; added new protocol for reminding participants of their assessments, as well as method for obtaining outcomes not collected at week 6 and month 3<br>Section 5.5.3 Compliance and Co-Interventions – Clarified participant follow-up and compliance plan.<br>Section 5.5.2 Data Storage – removed 'for a maximum of 7 days after all information has been entered into CTCC database' and replaced 'As soon as it ci completely entered in the CTCC system, the forms will be shredded' with 'After information has been entered into the database, it will be stored in the locked cabinet for up to 7 years after the study has been completed.'<br>Section 5.6<br>- Revised and clarified process for collecting, reporting, and reviewing adverse events (see Reactions and Discomforts section in Week 2/4 or Week 6 Assessment in Appendix A)<br>- Clarified reporting procedures for protocol violations<br>- Under 'Quality Analysis and Quality Assurance' section – revised frequency of Samueli/RAND site visits<br>Section 6.0: Timeline - Updated<br>Appendix A: added screenshots of all web-based assessments<br>Appendix D: removed Blackboard connect communication table; removed data plan pertaining to use of Blackboard connect; removed Blackboard connect scripts no longer being utilized<br>Appendix E: Data collection forms<br>- Added revised physician exam form<br>- Added revised treatment form
March 2013<br>Version 13	Section 5.6<br>Added procedures for 'Qualitative analysis and quality assurance' section

Table of Contents

Table of Contents 5

BACKGROUND 8

1.1 Introduction 8

1.2 Summary of Significance 14

2.0 STUDY DESIGN 15

2.1 Specific Aims 15

2.2 Sample and Methodology 16

3.0 OVERVIEW OF TRIAL ORGANIZATION 16

4.0 MAJOR STUDY COMMITTEES 17

4.1 Internal Steering Committee 17

4.2 External Advisory Committee 17

4.3 Data and Safety Monitoring Committee 17

4.4 Institutional Collaboration and Support 18

4.5 Publication Committee 18

5.0 RESEARCH DESIGN & METHODS 19

5.1 Study Population 19

5.1.1 Participant Recruitment 20

5.1.2 Eligibility Criteria 20

5.1.3 Randomization 21

5.1.4 Overview of Data Collection 21

5.2 Participant Visit Protocols 22

5.2.1 Baseline Visit (BL) 22

5.2.2 Exam 23

5.2.3 Treatment Visits 24

5.3 Primary Outcomes 27

5.3.1 Data Analysis 29
5.3.2 Sample Size 30
5.4 Reporting of Protocol Deviations 31
5.5 Data Monitoring 31
5.5.1 Data Management 31
5.5.2 Data and Safety Monitoring Plan. 32
5.5.3 Compliance and Co-Interventions 34
5.6 Reporting of Adverse Events 34
6.0 TIMELINE 38
References 39
Appendix A: Participant Web-based Study Assessments ..... **Error! Bookmark not defined.**
Baseline Interview ..... **Error! Bookmark not defined.**
Baseline Exam ..... **Error! Bookmark not defined.**
Baseline Assessment ..... **Error! Bookmark not defined.**
Week 2 and Week 4 Assessments ..... **Error! Bookmark not defined.**
Week 6 Assessment (Primary Endpoint) ..... **Error! Bookmark not defined.**
Month 3 Assessment ..... **Error! Bookmark not defined.**
Appendix B: Tobacco Course Handouts ..... **Error! Bookmark not defined.**
Presentation: Helping your Patients Quit- Guide ..... **Error! Bookmark not defined.**
Activity 1 - Invent your tobacco user ..... **Error! Bookmark not defined.**
Activity 2 – Motivation and Opportunity ..... **Error! Bookmark not defined.**
Activity 3 - Role-play using scenario cards ..... **Error! Bookmark not defined.**
HELPING YOUR PATIENTS ..... **Error! Bookmark not defined.**
QUIT TOBACCO ..... **Error! Bookmark not defined.**
Provider Instructions for Using the Personal Quit Plan ..... **Error! Bookmark not defined.**
The Personal Quit Plan ..... **Error! Bookmark not defined.**
The Personal Quit Plan Follow-Up ..... **Error! Bookmark not defined.**

Patient Materials .... **Error! Bookmark not defined.**

Appendix C: Tobacco Cessation DC questionnaire .... **Error! Bookmark not defined.**

Appendix D: Assessment Reminder and Follow-Up Protocol .... **Error! Bookmark not defined.**

Appendix E: Paper-Based Data Collection Forms .... **Error! Bookmark not defined.**

Appendix F: Participant Certification .... **Error! Bookmark not defined.**

BACKGROUND

1.1 Introduction

The focus of this study is on low back pain (LBP) for several critical reasons. First, LBP is well-recognized as a public health problem in both military and civilian populations. Second, ninety percent of LBP in clinical practice is diagnosed as "idiopathic," because its pathophysiology, diagnosis and treatment are not well understood.[1,2] This pervasiveness, as well as the lack of understanding of the condition, have led to a consensus among experts on the importance of an extensive research agenda focusing on clinically relevant scientific questions about the problem.[3,4] Third, LBP has no cure, or even a "silver bullet" medical approach. Carey et al. (2009) recently conducted a survey to determine health care utilization patterns in patients with chronic LBP.[1] They found high health care utilization in this group, with an average of 21 visits to an average of 2.7 provider types annually. Many of the tests and treatments used did not conform to evidence-based practice. The authors conclude that 1) care utilization for chronic LBP is very high, including high rates of use of advanced imaging, narcotics, and physical treatments; 2) use of evidence-based treatments is low when compared with current best evidence; and 3) many treatments appear to be over-utilized. Fourth, the most recent comprehensive survey of complementary and alternative medicine (CAM) use in the US showed that 17% of those who use CAM treatments, including chiropractic, do so for back pain or problems, making it the most common and costly condition for which chiropractic treatment is sought.[5] Fifth, a cadre of randomized clinical trials (reviewed below) generally shows a positive benefit from chiropractic manipulative therapy (CMT), the cornerstone of chiropractic practice, for LBP. Finally, investigators for this study are able to present pilot data demonstrating the effectiveness of CMT in active duty military personnel for LBP (reviewed below).

Overview of Low Back Pain and the Use of Chiropractic Manipulative Therapy: LBP is a well-recognized public health problem in the US with estimates of overall prevalence ranging from 12-33% and one year prevalence ranging from 22-65%.[6] Back pain is the 2nd most frequent reason for physician visits, the 5th for hospitalizations, and 3rd for surgery. There is no question it is debilitating to society: in the civilian sector, work-related cases result in over one million lost work days per year and nearly 15 times that many visits to medical doctors. Care and productivity cost estimates in the US are at least $26 billion or more annually.[7,8]

Low Back Pain in the Military

Low back pain (LBP) is the most common cause of disability worldwide, but it is even more prevalent in active duty military personnel. A recent study published in Archives of Internal Medicine reported that back pain is most prevalent in soldiers in combat deployments, and it is among the most likely conditions to interrupt combat duty.[9]

In fact, more than 50% of all diagnoses resulting in disability discharges from the military across all branches are due to musculoskeletal conditions.[10]. A study by Jones and Hanson (2010) found that

payments to veterans amounted to $485 million to newly disabled Army personnel in 1993.[11] Lincoln and colleagues examined the natural history and risk factors that led to disability among US Army personnel.[12] Their study was a retrospective cohort that followed active-duty Army personnel from initial hospitalization for a musculoskeletal-related condition for years 1989-1996 through the development of physical disability up to 1997. To be included in the study, subjects had to have been on active duty at the time of hospitalization, been hospitalized for a musculoskeletal disorder or severe sprain/strain during 1989-1996, and have completed a health risk appraisal during that period. Data were derived from the Total Army Injury and Health Outcomes Database. The outcome of interest, disability, was defined as having been assigned the following status at a medical evaluation board during the study period and up to 1997:

- Permanent disability/retirement (disability rating of at least 30% or having 20 years of service)
- Severance without benefits (disability rating of less than 30% and having less than 20 years of service)
- Temporary disability (similar to permanent disability except for the possibility that the condition will change within the next five years and enable the subject to return fit for duty)

The data from this project demonstrated that intervertebral disc degeneration had the highest cumulative disability at 6 and 12 months, and that the five-year cumulative risk of disability was highest for intervertebral disc degeneration, intervertebral disc derangement and non-specific back pain. One surprising finding was that personnel at highest risk included those with 1-4 years of service, similar to the group under study in this project, compared to those with more than 10 years of service. Back conditions were associated with the highest 5-year cumulative risk of disability discharge, making them a critical issue with regard to troop and personnel readiness.

Back pain has been characterized as “The Silent Military Threat,” because of its negative impact on mission readiness, and the degree to which it compromises a fit fighting force. For these reasons, military personnel with LBP need a practical and effective treatment that relieves their pain and allows them to return to duty quickly, but also one that preserves function and military readiness, addresses the underlying causes of the episode and protects against re-injury. The DoD/VA clinical practice guidelines (CPG’s) for the treatment of LBP offer wide options for care. These include screening for “red flag” indicators of potential surgical or medical urgencies, and first-line treatments including NSAID’s and acetaminophen; patient education on the importance of exercise; and monitoring and documentation of clinical course. However, they are generally focused on standardizing minimum care and documentation that can be applied in all practice settings where service members are treated, and less focused on long-term strategies for the minimization of lost duty time and for secondary prevention. The program of research proposed herein is aimed at meeting the needs to establish evidence-based standards of care for acute, sub-acute and chronic LBP, which can be integrated in to the healthcare systems for all active-duty personnel, including combat-deployed troops and Special Operation Forces.

Definition of Chiropractic Manipulative Therapy

Chiropractic manipulative therapy (CMT) is a manual therapy commonly used to treat low back pain and is the cornerstone of chiropractic practice. The procedure in its broadest definition describes the therapeutic application of a load (force) to specific body tissues (usually vertebral joints). CMT can vary in terms of its velocity, amplitude and frequency, as well as anatomical location, choice of levers, and direction of force application.[13,14] In a course of care, the dosage of CMT (e.g. in terms of treatment frequency) can also vary significantly. Numerous procedures are used in practice, but a more detailed and quantitative biomechanical picture of CMT is emerging from studies on the forces applied and the resultant kinetics and kinematics.[14-16] CMT can be divided into two broad categories by their force/time profiles: those maneuvers that deliver a high-velocity low amplitude load or impulse “thrust” to body tissues (HVLA- CMT) and those that deliver a low-velocity variable amplitude load (LVVA- CMT).[17-19] Velocity refers to the speed with which a load is applied, while amplitude refers to the depth of the thrust into body tissues. HVLA manipulations are called “adjustments” by chiropractors, or “manipulation” by other professionals. Both terms distinguish it from LVVA maneuvers, which most experts label as “mobilization”.[14,20] HVLA- CMT procedures are often associated with a cavitation sound or “crack,” as synovial joint linings are quickly separated. In contrast, in low-velocity maneuvers, the loads are applied slowly, and the amplitude (depth) of each load may vary depending on the clinical situation. Both forms of CMT are typically used by most doctors of chiropractic and both will be used in this study.

Figure 1. Proposed Mechanisms of Action for CMT

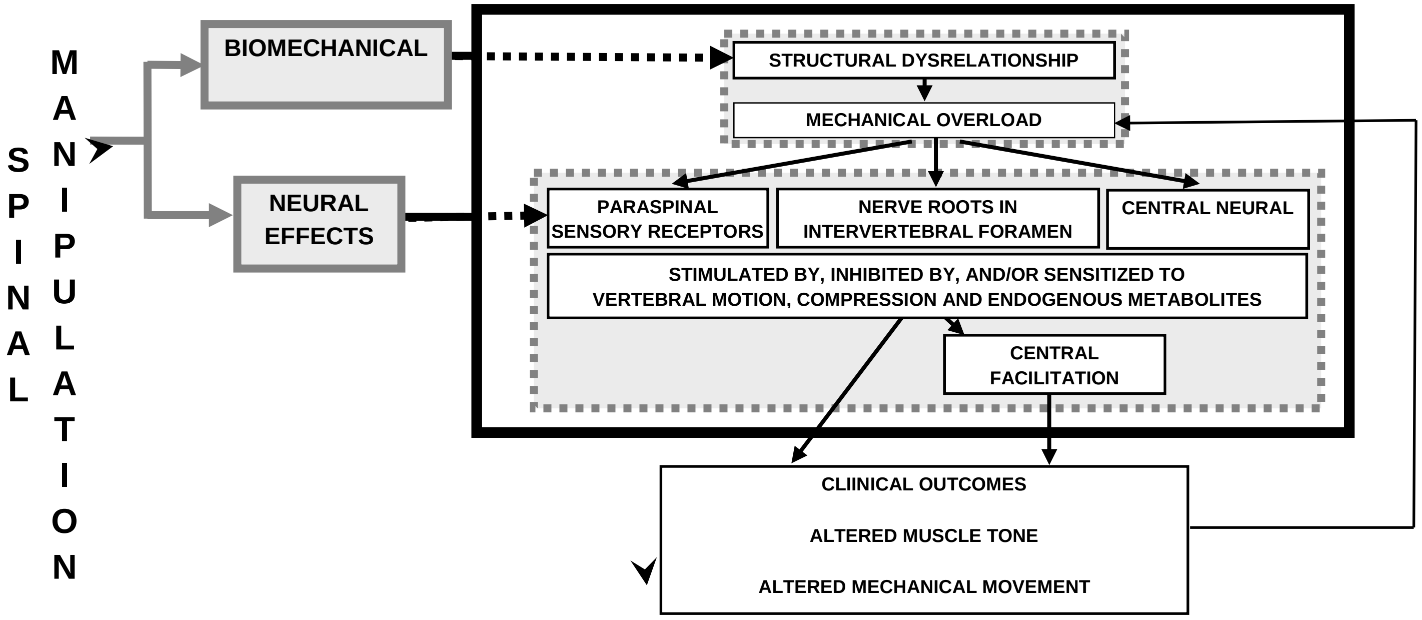

Figure 1 presents a flow diagram depicting one of the predominant theoretical paradigms used to understand the physiological basis for CMT [21], developed by Dr. Joel Pickar at the Palmer Center for Chiropractic Research. It shows the potential mechanistic relationships between segmental

biomechanics, the nervous system, chiropractic manipulation therapy and end-organ physiology. The figure is not all inclusive; circulatory and immunological changes in response to CMT have also been suggested.[22,23] The dark black line represents a "black box" of mechanisms by which a disordered motion segment is thought to contribute to a patient's symptomatology in general. The facet joints are thought to become restricted, disturbed or functionally asymmetric due to paraspinal muscle dysfunction, synovial meniscoids or inclusions trapped between articular surfaces of the facet joints, intra-articular or myofascial adhesions and/or distortion of the annulus fibrosus.[24-29] Any of these vertebral dysrelationships may produce a biomechanical overload with effects on nerve roots or spinal cord directly or via meningeal traction, or on surrounding paraspinal tissues that secondarily alter their physiology including the signaling properties of mechanically- or chemically-sensitive neurons in the paraspinal tissues.[30] The changes in neural activity are thought to modify neural integration, either by directly affecting reflex activity and/or by affecting central neural integration within motor, nociceptive and autonomic neuronal pools. Pain, discomfort, altered muscle function or autonomic function comprises the signs or symptoms that might cause patients to seek CMT. Mechanical treatment using manipulation, then, theoretically alters the inflow of sensory signals from paraspinal tissues or activity of central neurons either by direct effects on the nervous system or via indirect effects on tissue biomechanics. A goal of CMT is to remove joint restrictions and "restore maximal, pain-free movement of the musculoskeletal system".[22,27,31,32]

Randomized Controlled Trials (RCTs) of chiropractic manipulation therapy

The 1975 NINCDS conference on the "Research Status of Spinal Manipulative Therapy" pointed out the lack of any significant research to justify claims made by chiropractors or any other practitioner of CMT.[33] By 1992, at least 45 RCTs of all forms of CMT for the treatment of acute, sub-acute and chronic LBP have been published.[8] Thirty-one favored CMT over the comparison treatments in at least a subgroup of patients, and the rest found no significant differences[8].

The majority of systematic reviews are in agreement that CMT appears to reduce pain and disability at least some of the time to some degree for a significant proportion of LBP patients.[34-37] They also agree on the highly variable quality of extant trials and reviews [38,39], on the inconsistent results, small effect sizes, and large variation in outcomes.[40,41] Part of the problem is attributable to poor trial methodologies, part to poor execution and reporting, and part is probably due to the large variation in CMT treatments that have been studied. Finally, it is suspected that there is large and as yet unidentified heterogeneity in LBP patients.[42]

The unexplained inconsistency in RCT results currently obscures the true utility of CMT for LBP patients and contributes to ongoing debate. Current RCT evidence has not ruled out CMT's potential effectiveness for LBP, but the appropriate role of CMT in treating LBP has not been confirmed; thus additional high quality RCTs are required.[11,37,43,44] Successful completion of the study aims outlined in this proposal will not only provide critical information regarding the impact of CMT in military populations; it will also represent the largest multi-site randomized clinical trial conducted to evaluate CMT to date.

Chiropractic manipulation therapy and Chiropractic

CMT delivered by doctors of chiropractic is commonly used by LBP patients. Although CMT is a treatment procedure used by both conventional and CAM professions, chiropractors provide over 90% of CMT in the US.[4] Chiropractors report using some form of CMT on at least 80% of their patients.[45,46] At least 7.5% of the US population seeks care from chiropractors annually, representing approximately 190 million patient visits.[5,47] A national survey of patterns and perceptions of care found that 20% of those reporting back or neck pain sought chiropractic care, while 37% sought conventional care.[48] Surveys suggest that patients are highly satisfied with chiropractic care.[49,50] More than 60% of chiropractic patients report their care as being "very helpful," while 27% report the same for conventional medical care.[48]

Comparative Effectiveness Research

This protocol describes a comparative effectiveness trial (CER). The Institute of Medicine definition [51] for Comparative Effectiveness Research (CER) is:

"CER is the generation and synthesis of evidence that compares the benefits and harms of alternative methods to prevent, diagnose, treat and monitor a clinical condition, or to improve the delivery of care. The purpose of CER is to assist consumers, clinicians, purchasers, and policy makers to make informed decisions that will improve health care at both individual and population levels."

The Agency for Healthcare Research and Quality (AHRQ) states that their Effective Health Care Program purpose is to fund research that provides reliable and practical data that can inform decisions in clinical practice.[52] CER has been identified by several names in the past: pragmatic trials, head-to-head trials and practical clinical trials.

Some writers have contrasted CER (or Practical Clinical Trials – PCTs) trials to explanatory trials [53], which are hypothesis driven and usually done with the hope of revealing the biological effect of a treatment. In contrast CER or pragmatic trials are done to assist decision makers. Zwarenstein and Treweek (2009) note that there is often a mismatch between the clinical setting in which decisions must be made and the RCTs (explanatory trials to test hypotheses).[54] They note "evidence from an explanatory trial is unlikely to inform a pragmatic question, nor vice versa…".[51]

One key feature of CER is a focus on effectiveness rather than efficacy.[55] Efficacy establishes a causal connection between an intervention and a specific outcome. To do this it is necessary to control all biases so that the only thing contributing to the outcome is the specific intervention. This requires that the enrollments in the trial are controlled and random, that the intervention is standardized and controlled (it must be constant and identical across all subjects), that the populations are homogeneous and that the outcome measures are standardized and objective. These trials will have at least two arms, one where the intervention is given and one where a sham treatment or a placebo is given. Individual subjects are randomly allocated to one of the arms. Neither the provider of the therapy nor the patient should know which arm of the trial the patient is in (double blinding).

The problem is that to achieve the kind of controls you need for an efficacy RCT, you end up creating a situation that is very different from the normal way in which the therapy will ultimately be practiced in the real world. The exclusion criteria for the subjects in the trial may be so restrictive that the very sub-populations the provider wants to treat were not even included in the trial. In summary, the evidence from efficacy RCTs may be rigorous but not relevant to the real world of practice. Most methodologists describe pragmatic trials as enrolling all patients to whom health care providers might offer the intervention, allowing clinicians to administer the intervention and co-interventions without restrictions and measuring patient-important outcomes.[56] Maclure (2009) in discussing how to describe pragmatic trials to policy makers, states "pragmatic trials are real-world studies for decision whereas explanatory trials are specialized studies for information."[57] The investigative team has chosen a modified comparative effectiveness approach for the proposed study that combines elements of both efficacy trials and pragmatic trials. We believe that this is the best way to answer questions that will be meaningful to policy makers as they consider the appropriate role for CMT in active duty military populations. Further, our experience in the conduct of clinical trials in military treatment facilities (aka site) has shown us that this type of trial is feasible to conduct in busy clinical practice settings.

Smoking in the Military

Smoking is a known major public health concern in the United States. Recent statistics indicate that 20.6% of American adults, or 46 million people, smoke.[58] Smoking is a proven risk factor for major illnesses, such as lung cancer, chronic obstructive pulmonary disease (COPD), heart attacks, stroke and a host of other related cancers and vascular syndromes.[59] The individual lifelong impacts of smoking include decreased overall quality of life, significant disability that affects work life and productivity, as well as decreased life expectancy. The societal impact in the United States has been estimated as: number of smoking-related deaths per year (450,000); productivity loss due solely to smoking-related premature deaths of workers ($97 billion); and medical costs for smoking-related illness ($96 billion).[58]

Smoking in the military is associated with even more disturbing statistics. A 2009 report by the Institute of Medicine reported an overall smoking rate of more than 30% in active duty (AD) personnel. Additional data suggest that as many as two thirds of military personnel deployed use tobacco. The report recommended that the DoD close "the pipeline of new tobacco users entering the military and promote cessation programs to ensure abstinence." Furthermore, it urged the military to "treat tobacco use in the same way as other health-related behaviors, such as alcohol abuse and poor physical fitness."

The 2005 DoD Survey of Health-Related Behaviors reported that smoking rates were highest among members of the Army and Marine Corps (38.2% and 36.3%, respectively); smoking was more prevalent among men than women (37.8% v. 35.5% overall, respectively); and that the 18-25 year old segment of the military has higher smoking rates (38.7%) than the 26-55 year-olds (35.7%).[60]

The high costs of smoking in the military, reportedly $564 million [61], are driven higher still when one considers literature indicating that smokers are more likely to fail trainings and PT tests;[62-64] indulge in other substance abuse behaviors; and to sustain injuries, particularly musculoskeletal injuries.[65,66] Since the heaviest tobacco use occurs in the same populations that characterize young combat soldiers and

Marines, who are more prone to injury, and given the high overall rates of tobacco use in the military, it is appropriate that programs targeting smoking cessation be evaluated in military patients seeking care for LBP.

Research has linked smoking with back pain, identifying it as a risk factor for back pain severity and duration.[67-69] Smoking has also been implicated as self-medication for back pain.[70] It therefore comes as no surprise that chiropractors have taken increasing interest in promoting cessation of tobacco use by their patients.[71,72] Gordon et al found that chiropractors in Oregon advise their patients to quit smoking and were interested in learning more about helping their patients quit smoking.[73] The authors subsequently developed and pilot tested a tobacco cessation program tailored for use in private chiropractic practice. Cessation outcomes were positive and the program was found to be promising.[74]

It should be noted that no randomized trials have assessed the effect of CMT for smoking cessation.[36] There is no current reason to suspect a direct effect on tobacco cessation. However, CMT might help relieve back pain, which in turn might reduce patients' need to self-medicate with tobacco. A recent study by Gordon et al provides both preliminary data and a smoking cessation program for delivery by doctors of chiropractic, which form the model for the proposed nested study of tobacco cessation.[74] We will attempt to replicate these findings in active duty smokers with LBP.

1.2 Summary of Significance

The significance of this study is high. Low back pain (LBP) is a prevalent public health problem in both the military and civilian populations. Currently a clear "gold standard" medical treatment for low back pain does not exist and studies show that evidence-based guidelines are rarely used in general practice. Thus, there is a need to consider innovative treatment options for chronic diseases such as LBP. Our preliminary data suggest that chiropractic manipulative therapy (CMT) in addition to standard medical care may be superior to standard medical care alone in active duty service members. In addition, doctors of chiropractic are well positioned to provide information to support tobacco cessation. The results from this randomized clinical trial, with a nested tobacco (smokers and smokeless tobacco users) cessation intervention will provide critical information regarding the health and mission-support benefits of chiropractic health care delivery for active duty service members in the military.

2.0 STUDY DESIGN

2.1 Specific Aims

The primary objectives of the proposed study described in this clinical protocol are to: 1) assess the effectiveness of chiropractic manipulative therapy (CMT) for pain management and improved function in active duty service members with low back pain that do not require surgery; and 2) to assess the impact of a chiropractic intervention on smoking cessation. This study will focus on active duty service members who are not deployed in theater. The primary hypothesis is that CMT, in addition to conventional medical care, will provide significantly better pain relief and improved functional status in volunteers with LBP than conventional medical care alone. A secondary hypothesis is that education and monitoring of tobacco habits provided during routine chiropractic care visits for LBP will result in a significant decrease in the average number of tobacco use per week among those who self-identify as a tobacco user at baseline. Please refer to Figure 2 ‘Chiropractic Trials Logic Model’ for a snapshot of the program design.

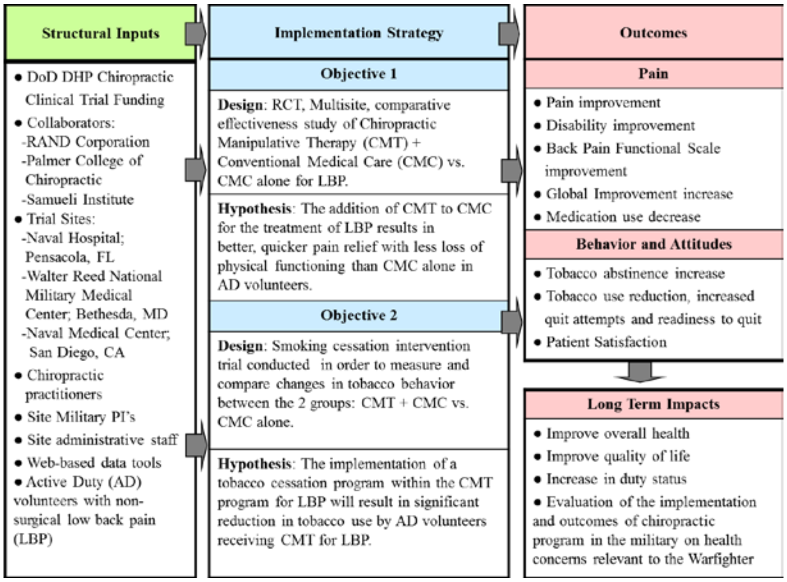

Figure 2. Chiropractic Trials Logic Model.

SPECIFIC AIMS

A multi-site RCT will be conducted at 3 military sites [Walter Reed National Military Medical Center in Bethesda, MD (n = 250); Naval Hospital in Pensacola, FL (n = 250); Naval Medical Center in San Diego, CA (n = 250)] to accomplish the following two Specific Aims.

Specific Aim #1: To evaluate the pain and functional outcomes of CMT plus conventional medical care to those of conventional medical care alone in a total of 750 active duty military personnel ages 18-50 with non-surgical acute, sub-acute or chronic LBP.

Specific Aim #2: To measure the impact of a tobacco cessation program delivered by doctors of chiropractic in active duty volunteers receiving CMT for LBP.

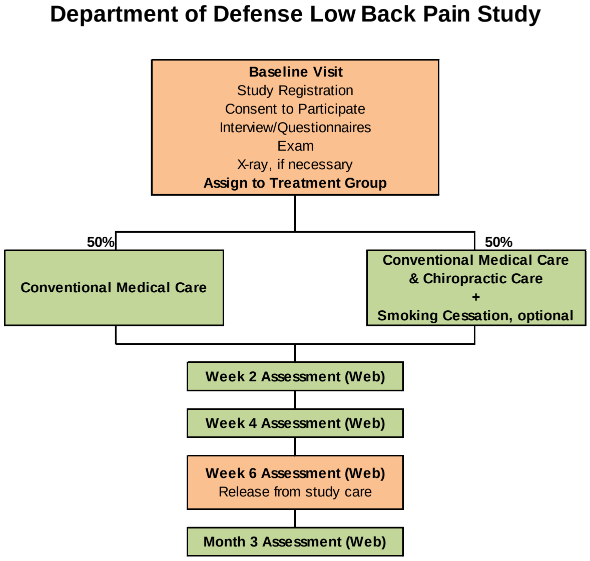

Figure 3. Study Flow Chart.

2.2 Sample and Methodology

To accomplish the specific aims, a multi-site Clinical Comparative Effectiveness Trial designed to rigorously compare the outcomes of CMT and conventional medical care (CMC) to CMC alone. Chiropractic treatment will include chiropractic manipulative therapy (CMT) plus ancillary physiotherapeutic interventions. CMC will be delivered following current standards of medical practice at each site. At each of the three participating sites, active duty military personnel, ages 18-50, who present with acute, sub-acute or chronic low back pain that does not require surgery will be randomized to one of the two treatment groups. Outcome measures include the Numerical Rating Scale for pain, the Roland-Morris Low Back Pain and Disability questionnaire, the Back Pain Functional Scale for assessing function, and the Global Improvement questionnaire for patient perception regarding improvement in function. Patient Expectation and Patient Satisfaction questionnaires will be used to examine volunteer expectations toward care and perceptions of that care. Pharmaceutical use and duty status data will also be collected. The PROMIS-29 will be used to compare the general health component and quality of life of our sample at baseline. As a secondary aim, this clinical trial will include a nested study designed to measure the impact of a tobacco cessation program delivered by a doctor of chiropractic.

3.0 OVERVIEW OF TRIAL ORGANIZATION

Responsibility for the conduct of the clinical trial described in this protocol is placed on the Palmer College of Chiropractic, in collaboration with RAND Corporation, the parent institution, and the Samueli Institute (SIIB). Please see the organizational chart in Figure 4.

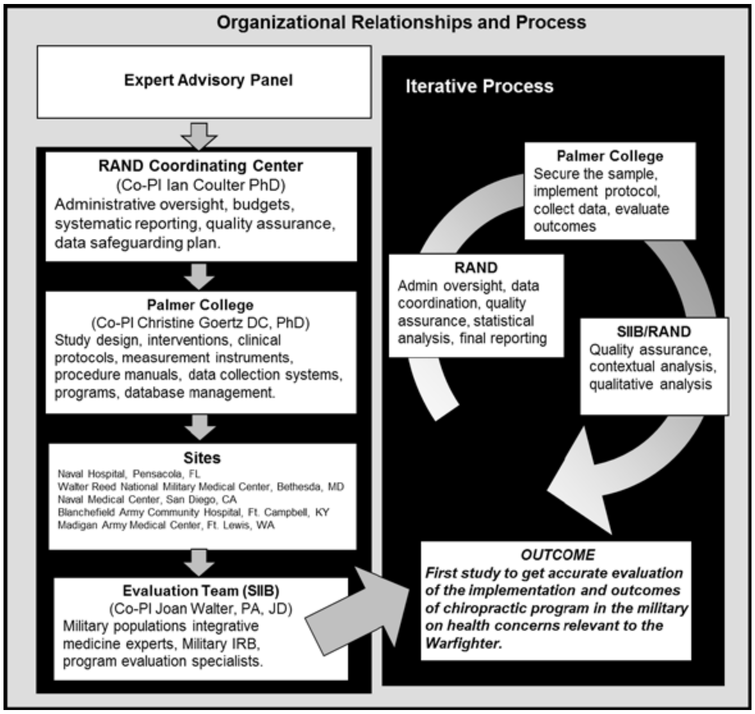

Figure 4. Organizational Plan

Dr. Christine Goertz is Co-PI of the grant, with the overall administrative and scientific responsibility for the success of the clinical trial described in this protocol. As Vice Chancellor for Research and Health Policy at Palmer College of Chiropractic Research, reporting directly to the Chancellor, she is in an excellent position to ensure the availability of institutional resources. She will work closely and continuously with the Internal Steering Committee (ISC) and Expert Advisory Committee (EAC) to assure progress, focus, coordination and synergy.

4.0 MAJOR STUDY COMMITTEES

4.1 Internal Steering Committee

Dr. Coulter will establish and chair an Internal Steering Committee (ISC) composed of the lead investigators and project managers from RAND, Palmer and Samueli Institute, as well as the site PIs. This committee will provide a wide range of input to manage the multi-centered trial. The primary purpose of the ISC is to share information, monitor progress, raise issues and solve problems and plan for future research. The ISC will make recommendations for policy or protocol additions or changes when necessary. In keeping with a governance structure most conducive to productive research, the ISC will be advised when major decisions regarding the study must be taken. ISC members have the responsibility to communicate and share appropriate decisions with their project staff.

Regular telephone meetings of the ISC will take place on a weekly basis, with RAND, Palmer and Samueli personnel required to attend by teleconference. Agendas will be prepared in advance of each meeting and minutes will be recorded, circulated and kept. These meetings will also be used to confirm the success or make recommendations regarding the more regular communication expected between Co-PIs. Site PIs and PMs will have one-on-one monthly meetings with the PI and lead PM at Palmer.

4.2 External Advisory Committee

The External Advisory Committee (EAC) will be the primary advisory body for the clinical trial and will assist the Co-PIs to meet its goals. The EAC will provide written annual reports to the project investigators focused on the following issues: 1) progress of research projects; 2) effectiveness of communication and collaboration between co-investigators; 3) use of resources; 4) changes to the original research plan; 5) the Co-PI's effectiveness; and 6) identified challenges, problems and proposed solutions. The EAC will first meet 3 months after the award date and then will meet yearly after that. The EAC is comprised of six individuals who have all agreed to sit on the Board. These individuals represent leaders in either the research community in LBP, the chiropractic research community or in the military. They include the following individuals: Anthony J Lisi, DC, National Director Chiropractic Services, Department of Veteran Affairs; Valerie Johnson, DC, Staff Chiropractor, VAGLA, Department of Veteran Affairs; Dan Cherkin, PhD, Senior Scientific Investigator, Group Health Research Institute; Marion McGregor, DC, FCCS(C), PhD, Canadian Memorial Chiropractic College; Scott Haldeman, DC, MD, PhD, Clinical Professor Neurology, UC Irvine; Reed Phillips, DC, PhD, NCMIC Foundation.

4.3 Data and Safety Monitoring Committee

The Data and Safety Monitoring Committee (DSMC) is a standing independent committee at the PCCR to provide an independent means of examining objectively accruing controlled trial data for indications of harm (from adverse events from the interventions applied or tested) as well as benefit.

The DSMC will monitor the overall conduct of the RCT described in this protocol. Responsibilities of the DSMC are: 1) to ensure the overall safety of participants in clinical trials conducted by PCCR investigators by protecting participants from avoidable harm and declaring clear benefit when there is proof beyond a

reasonable doubt; and 2) to provide DoD and the EAC with advice about the scientific and ethical conduct of clinical trials.

The DSMC will meet at least twice per year either in person or by teleconference. The DSMC will evaluate the adverse event data to protect the safety of study participants. If necessary, DSMC members will make recommendations to the Co-PIs and DoD regarding continuation, termination or other modifications of the trial based on observed adverse events of the treatments under study.

The DSMC is comprised of a biostatistician, medical physician, doctor of chiropractic and epidemiologists/clinical trialists, none of whom are affiliated with Palmer. The team biostatistician will prepare a study report for the DSMC including accrual plots and other enrollment data, data collection forms processing status, baseline characteristics of enrolled participants, follow-up and treatment compliance, protocol violations and all web-based reportable adverse events (see Reporting of *Adverse Events)* every 6 months.

4.4 Institutional Collaboration and Support

Scientific and institutional collaboration and commitment are key attributes to the success of the proposed project. During the planning process of the grant writing, the Co-PIs identified key investigators, core resources and institutions that demonstrated a high level of enthusiasm for developing this application. An *ad hoc* planning team was composed to consider and reach out to potential DoD partners based on common and complementary scientific interests, expertise, past experience and productivity. Enthusiastic letters of support were included in the original grant application from each Institution and Military site involved in the project. Before the grant was received, each of the partner institutions had already contributed considerable resources to the development of this joint project and each had committed the institution and its resources to successfully carrying out the project.

4.5 Publication Committee

The publication committee consists of all investigators and other invited individuals that have contributed scientifically to this study. This committee will meet six months after the onset of primary data collection and will continue meeting once a quarter with potential to bi-weekly toward the end of implementation. They will discuss potential papers, necessary data, projected journal submissions, conference presentations and timelines for each publication project.

5.0 RESEARCH DESIGN & METHODS

5.1 Study Population

Naval Air Station (Pensacola, FL) n = 250

The Naval Air Station (NAS) Pensacola has approximately 11,200 active duty personnel and is the primary training base for all Navy, Marine and Coast Guard aviators and Naval Flight officers. It is also home to the Navy Flight Demonstration Team (Blue Angels), Naval Air Technical Training Center and Training Air Wing 6. Health care is provided at the Naval Hospital Pensacola (NHP), a 108-bed hospital, as well as at the Naval Branch Health Clinic which supports approximately 12,000 active duty personnel ranging in age from 18 to 55 years old. Chiropractic services were established in September 2003 and are offered at the Chiropractic Clinic, which is part of the Sports Medicine and Reconditioning Team (SMART) clinic. The clinic sees approximately 600 new patients, and 3,500 to 4,000 total patient visits a year, all of whom are active duty personnel. Seventy percent of the patients are treated for low back pain. Of these, 30% are treated for acute pain, 50% for sub-acute and 20% for chronic pain. Dr. Greg Lillie is the treating chiropractor at this site.

Walter Reed National Military Medical Center (Bethesda, MD) n = 250

The 500-bed Walter Reed National Military Medical Center (WRNMMC) is the Navy's third largest health care delivery system, providing more than 12,500 ambulatory surgeries and almost 8,000 inpatient admissions each year. It is the designated hospital for Navy and Marine casualties returning to the continental United States from Operation Enduring Freedom (OEF) and Operation Iraqi Freedom (OIF). In 2006, there were approximately 7,960 hospital admissions, 455,503 visits to hospital clinics and an average daily patient load of 128. Since 2003, WRNMMC has treated more than 1,600 war wounded service members. The chiropractic clinic there is staffed by two chiropractors and additional supportive staff, who provide care for Army (33%), Navy (40%), Marine Corps (11%) and Air Force (15%) personnel based in the Washington, DC area. Navy personnel stationed at Washington Navy Yard, Naval Surface Weapons Centers in Indian Head, and Carderock, MD, the US Naval Academy and Naval Air Station Patuxent River are among the patients served, as are Marines stationed at Quantico and Marine Corps barracks in Washington, DC. Low back pain is the most common condition treated. The doctors of chiropractic at this site are William E. Morgan and Terence Kearney.

Naval Medical Center (San Diego,CA) n = 250

NMCSD sees about 3, 600 to 4,000 active duty patients per year, 20 to 25% of which are new patient exams. Chiropractic services are part of the Physical Therapy Department and are located in the acute care area at both the Naval Air Station and BUDS Medical (SEALs). Patients seeking services come from diffuse locations including medical sick call, acute care area, flight medicine, BUDS medical primary providers, physical therapy and other specialty providers. The chief complaint of chiropractic patients is spinal pain, most often LBP (40%). Approximately 20% of the patient population is special operation forces including Explosive Ordinance Demolition (EOD), Special Boats Units and SEALs, who often suffer LBP, premature disc degeneration, disc herniations and facet arthritis. The treating doctor of chiropractic is David W. Ward and Bart Green.

5.1.1 Participant Recruitment

5.1.1.1 Recruitment Strategies

The volunteers required for this clinical trial will be recruited primarily through the existing primary care triage system established at each site. Initial recruitment efforts will include efforts by primary contact providers with patients who present with an episode of acute, sub-acute or chronic LBP during routine patient care visits. Other recruitment efforts may include signs and brochures describing the study posted and distributed in the patient care clinic lobbies and review of existing patient records to identify patients with a previous diagnosis of low back pain.

Volunteers will not be compensated for participation in the study. Any recruitment materials will accurately reflect the study and will not advertise "free treatment" or promise a cure or benefit beyond that mentioned in the consent form protocols. In addition, all materials will be approved by local and central DoD/Palmer/RAND IRBs. Materials will not be coercive or offer undue inducements. We do not anticipate recruitment of vulnerable populations for this study.

5.1.1.2 Enrollment of Women and Ethnic as well as Racial Minorities

The inclusion of women and minorities will be proportional to that found in the active duty military population. General demographics on military populations indicate a 14.8% female distribution. Ethnic/minority distribution is as follows: 17.6% African American, 8.8% Hispanic and 9.2% other (Bray, Olmsted, et al, 2006). All reasonable attempts will be made to include representation from women and ethnic and racial minorities proportional to that found in active duty service members across the US.

5.1.2 Eligibility Criteria

All participants must meet the following inclusion and exclusion criteria.

**Inclusion Criteria**
Age $\geq$ 18 and $\leq$ 50
Diagnosis of acute, subacute or chronic low back pain
Ability to provide voluntary written informed consent
Active duty at one of the three participating military sites
**Exclusion Criteria**
LBP from other than somatic tissues as determined by history, examination and course (i.e., pain referred from visceral conditions)
Co-morbid pathology or poor health conditions that may directly impact spinal pain. Volunteers who have case histories and physical examination findings indicating other than average good health.
Bone and joint pathology contraindicating CMT. Volunteers with recent spinal fracture (within the last 8 weeks), recent spinal surgery (within the last 12 weeks), concurrent spinal or paraspinal tumor(s), spinal or paraspinal infection(s), inflammatory arthropathies and significant / severe osteoporosis will be referred for appropriate care.
Other contraindications for CMT of the lumbar spine and pelvis (i.e., unstable spinal segments, cauda equine syndrome)
Pregnancy or planning to become pregnant within 8 weeks

Exclusion Criteria Cont'd
Altered mental capacity as determined by the clinician (e.g. nonsensical statements, not oriented to time and place)
Use of manipulative care for any reason within the past month
Unwilling to provide phone and electronic contact information
Unable to confirm that they will not be transferred during the active phase of the study: i.e., deployment, receive orders for a distant duty assignment or training site or otherwise be absent from the current military site over the next 8 weeks (active study participation period).
Does not agree to be enrolled regardless of group assignment
PTSD Classification

5.1.3 Randomization

A total of 750 active duty (AD) personnel ages 18-50 with acute, sub-acute or chronic LBP will be randomized to one of the two treatment groups. Randomization will be done by using an adaptive computer-generated minimization treatment allocation to balance volunteer characteristics between groups on the baseline factors of sex, age, LBP duration and baseline Numeric Rating Scale (NRS) measurement. The site project manager will access the Treatment Assignment Module in the password-protected web database system on a secured computer network and pull down the volunteer ID. The minimization algorithm will run and produce the coded treatment assignment. The treatment group assignment and date, time and study personnel ID will be stored in the SQL database. All study personnel are blinded to the next treatment assignment. The back-up treatment assignment protocol is by predetermined sequentially numbered, opaque envelopes and to be used when the web-based system for randomization is unavailable.

5.1.4 Overview of Data Collection

The Manual of Operating Procedures (MOOP) will be created in Year One and posted on a password-protected secure server with a user friendly web interface. The MOOP will include current versions of consent and HIPAA forms, paper back-up of all web data collection forms, and all protocols and procedures that are standardized across and within sites.

Using the model that was successful in conducting our pilot study at Ft Bliss, Palmer will hire site project managers for each data collection site. The site project managers will be trained and supervised by an experienced project manager at the Palmer Center for Chiropractic Research. Site project managers will have the overall day-to-day responsibility for ensuring the completion and accuracy of data collection. The data collection instruments and timing for data collection efforts are indicated in Table 1.

Table 1. Data Collection Schedule

Item	Baseline-Interview	Baseline-Questionnaire	Week2 Questionnaire	Week4 Questionnaire	Week6 Questionnaire	Month3 Questionnaire
NRS		x	x	x	x	x
RMDQ		x	x	x	x	x
BPFS		x	x	x	x	x
Bothersomeness		x	x	x	x	x
PROMIS - 29		x			x	x
Healthcare Utilization & Medication Use		x	x	x	x	x
Tobacco Use/Dependence		x			x	x
Patient Satisfaction/ Global Improvement (modified VAS)			x	x	x	
Patient Expectation		x				
Self-report type of care received			x	x	x	
AE			x	x	x	
Duty Status	x		x	x	x	x
LBP history, clinical findings and prior care	x	x				
Sociodemographic characteristics	x	x				

Data will be collected from participants at baseline, Week 2, Week 4 , Week 6 (at the end of active care), and Month 3. The primary endpoint is at Week 6. The baseline assessment will be scheduled as in-person visits with the site project manager. All other follow-up assessments will be collected via a web-based data collection system.

We will attempt to obtain outcomes data from all participants in the trial, including those who never attend, who drop out of care, or who move away. It is required for all participants to have cell phones and an active email address. Therefore, follow-up reminders will be sent via telephone calls, text messages and emails.

5.2 Participant Visit Protocols

5.2.1 Baseline Visit (BL)

At this baseline visit, the site project manager will explain the handling of data and personal health information as dictated by HIPAA and guide potential participants through the informed consent process. This interview will be held in a room with a closed door to insure volunteer privacy. During the informed consent process, the site project manager will explain the study requirements and provide a study flow chart (Figure 3) to the potential participant. This discussion will include an explanation of the interview time commitments, examination procedures (which may or may not have already occurred), follow-up assessments, intervention commitments, potential risks of participation, potential benefits of participation, what to do in case of an adverse experience, and the option for discontinuing study

participation. In addition, the site project manager will answer any questions the participant may have about chiropractic care and the research study requirements and commitment. The study staff will ensure that volunteers understand and agree to all aspects of participation before proceeding.

Volunteers will have the opportunity to discuss the implications of participating in the study at the baseline visit or at any time thereafter if questions arise. These points include the following:

1. Volunteers who qualify for the study will be randomly assigned to one of two treatment groups. One involves conventional medical care only and the other involves chiropractic care in combination with conventional medical care.
2. All exams (including screening x-rays) and treatments will be provided at no charge to the volunteer.
3. Investigators cannot predict whether the treatments will be effective for each volunteer. However, based on previous evidence and clinical experience, there is a likelihood that volunteers will experience improvement in their LBP condition.
4. Potential study participants must be screened for eligibility, and if they are not eligible for the study they will be told so at that point, and will continue to receive care at the site.
5. Volunteers are expected to meet all scheduled appointments and complete all study questionnaires, interviews and tests.
6. Volunteers are expected not to initiate other types of manual or medical care for their LBP during their involvement in the study and to inform their study clinician if such treatment, or treatment for any other health problem, becomes necessary.

Once informed consent has been obtained, both the participant and site project manager will sign and date the consent form. The participant will receive a copy of the informed consent document. If the participant requests documentation about their participation in the study that does not contain protected health information, they will be given a participant certificate (Appendix F).

5.2.2 Exam

As discussed in recruitment strategies (section 5.1.1.1) there are 2 ways in which a potential participant enters into the study. They can either be recruited directly from their acute or primary care provider within the site clinic system or through recruitment efforts such as signs and brochures.

When military personnel present in the site clinic system, they meet initially with an independent duty corpsman (IDC) (corpsmen with specialized medical training) or other primary care provider (PCP) prior to having care rendered. The PCP or a IDC will be informed in advance that patients seeking care for back pain may be eligible for the trial and will be trained to complete an exam form which assesses study eligibility (Appendix E). When the medical examination is complete and the provider has given the military personnel their recommended conventional medical care treatments, the exam form will be given to the site project manager. S/He will key enter items from the study eligibility form into the secure module during the baseline interview after the informed consent document (ICD) has been signed. If a potential participant does not have their baseline interview completed within 2 weeks of the exam, then a new exam form and interview will need to be completed.

If study participant is recruited through other recruitment efforts, they will first go through the baseline visit and then be given the exam form. The site project manager will assist the potential participant in obtaining an appointment with a primary care provider or an independent duty corpsman who will be able to complete the exam form and provide them with conventional medical care as required in this study. The form will be returned to the site project manager, who will enter the information into the secure web module. After the form has been entered into the system, the participant will know if they meet all eligibility criteria. If so, they will be randomized to a treatment group (per section 5.1.3).

5.2.3 Treatment Visits

Conventional Medical Care

Conventional medical care may include the following: a focused history and physical examination; limited diagnostic imaging restricted to select volunteers (i.e., for example, those with radiculopathy); education about self-management, including maintaining activity levels as tolerated and local ice/heat application; pharmacologic management with the use of analgesics and anti-inflammatory agents; and additional therapies that may be applied for volunteers not responding to the initial interventions, including physical therapy and referral to a pain clinic.

Chiropractic Care & Conventional Medical Care

Volunteers in the chiropractic care groups will receive the same care listed above in the conventional medical care group, as well as chiropractic manipulative therapy (CMT). CMT will occur from a doctor of chiropractic over a six week period of time. We will set an *a priori* treatment schedule of up to 2 times per week for six weeks for this study; for a total of up to 12 total visits. It is conceivable that a participant may not medically need this number of visits. Therefore, as with conventional medical care, the treatment plan will be based on the patient's baseline evaluation by the chiropractor, the severity of the patient's condition, and how they respond to treatment. Two times a week was chosen as this frequency is a common treatment schedule seen in general clinical practice and was used in our pilot work at Ft Bliss. A duration of six weeks was chosen over the four weeks used in our pilot because in this study we will be including volunteers with sub-acute and chronic low back pain, which generally requires a longer treatment period. Following numerous discussions with the chiropractic clinicians at the three sites participating in this trial, the investigators have concluded that at some sites it may not be feasible to follow a tightly prescribed treatment schedule. Many of the DoD doctors of chiropractic frequently have waiting times of up to two weeks for a new patient, with a similar wait for additional patient visits. To address potential differences among sites on this issue, we will take the following steps: 1) each site will have a large enough sample size to detect significant differences between groups; 2) we will carefully track the number of visits each volunteer received and the timing of those visits; 3) investigators at RAND and SIIB will use both quantitative and qualitative methods to evaluate the potential impact that varying treatment schedules has on patient care outcomes. At each treatment visit, the study clinician will determine which of the two most common therapeutic approach(s) to consider with each participant: high-velocity, low-amplitude (HVLA) or low-velocity, variable-amplitude

(LVVA). An informal survey of the doctors of chiropractic participating in this study confirmed that these two treatments are most commonly used in active duty service members. Thus, treatment will be limited to these two techniques for the purposes of this study.

The clinician will decide which form of CMT to use based primarily upon the diagnosis and combination of co-morbid or complicating diagnoses. The volunteer's previous response to care (if known), flexibility and mobility, and general condition are also considered. Clinicians then make a second decision regarding the application (location and direction) of CMT. This decision is most often based upon the diagnosis; however, other items are considered such as tenderness, hypertonicity, hypomobility, positions of relief and provocation, imaging findings (e.g., spinal curvatures, degeneration, spondylolisthesis) and other factors individual to the case.

Lastly, the clinician considers what other forms of treatment would benefit the volunteer. Rehabilitation exercises (attended or at-home), passive stretching, neuromobilization techniques, manual muscular therapies (e.g., ischemic compression, friction massage), counseling (proper movement, activities and nutrition), or other modalities such as ultrasound, electrical neuromuscular stimulation (e.g. interferential current), heat, ice, taping and bracing may also be used as adjunctive therapies.[75] The type of CMT and adjunctive therapies used in the study will be carefully tracked throughout the study.

At each chiropractic visit, a treatment form will be completed (Appendix E) by the chiropractor. All forms will follow the data storage collection procedure described in Section 5.2.

Tobacco Cessation

Investigation of a tobacco cessation program delivered by doctors of chiropractic will be imbedded in the LBP trial. Volunteers randomized to the CMT who self-identify as tobacco users (i.e. smokers or smokeless tobacco users) at baseline and agree to participate by signing the informed consent document will receive the program. The tobacco cessation program to be used is based on the "Clinical Practice Guidelines for Treatment of Tobacco Use and Dependence" of Fiore et al.[76,77] These guidelines promote the use of the "5As" of a tobacco cessation intervention to be delivered by health care practitioners. It has been refined for dental and chiropractic practice by Gordon et al (R21 DA021349) and adapted for a large RCT in public dental clinics.[74,78] Volunteers for the LBP trial will be screened to identify tobacco users and level of interest in quitting as in Gordon et al.[74,78] Smokers will be defined as persons who have smoked at least one cigarette, cigar, or pipe in the last 7 days. Smokeless tobacco users will be defined as anyone taking at least one dip or

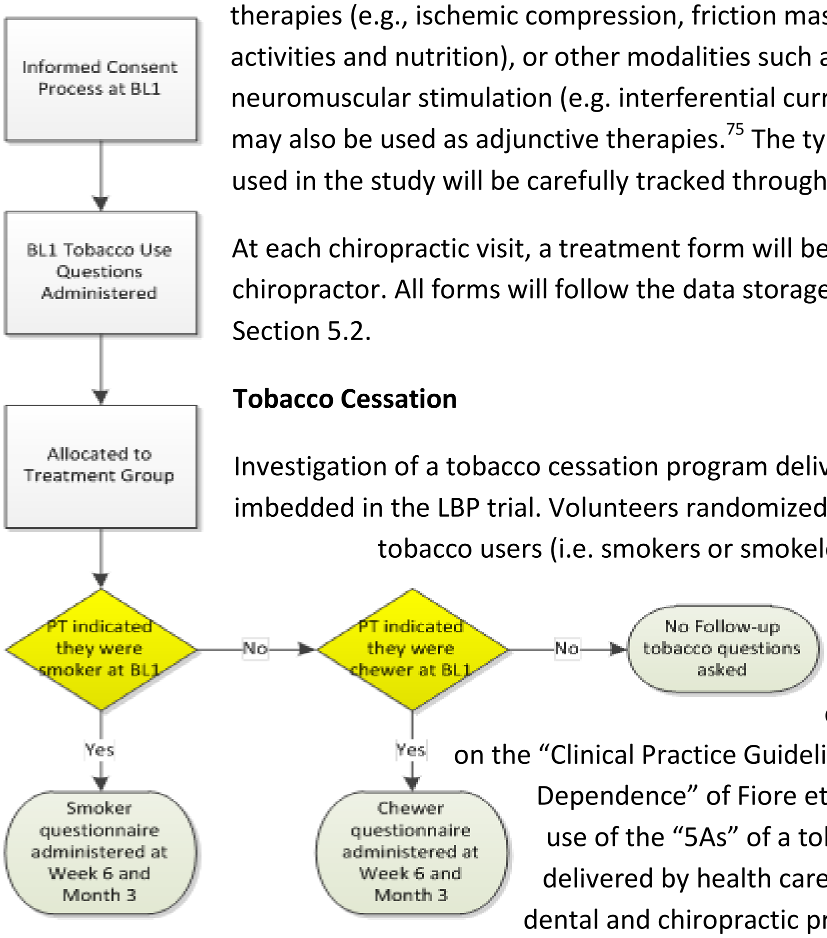

Figure 5. Tobacco Cessation Study Flow Chart.

chew in the last 7 days. Willingness to participate in the tobacco cessation component of the study will be documented through written informed consent. Those who wish to participate in the LBP study but not the tobacco cessation program will still be allowed into the LBP study as participation in the nested tobacco cessation study is optional.

Prior to initiation of data collection, study chiropractors will attend a 3-hour webinar training session on delivering the intervention.[74,78] (Handouts and lecture items are in Appendix B.) The webinar will include a PowerPoint presentation to present the tobacco cessation program. Refinements may be made to address study clinic operating procedures. Brief follow-up training sessions will be required every six months The outline for the webinar is: 1) Tobacco Cessation in Chiropractic Setting; 2) Tobacco-Related Health Problems; 2a) Chronic Pain/Musculoskeletal Problems; 2b) Decreased Healing; 2c) Respiratory Problems; 2d) Heart Diseases; 2e) Allergies; 2f) Diabetes; 2g) Macular Degeneration; 3) Helping your Patients Quit Tobacco; 3a) Patient Flow; 3b) Ask about Tobacco Use; 3c) Ask Assessment Questions; 3d) Advise-Relate Findings & Give Direct Advice to Quit; 3e) Arrange Help for Quitting; 4) Assess Readiness to Quit; 5) What about "Hard Core" Users?; 5a) Motivational Interviewing; 5b) Express Empathy; 5c) Promote Patient Autonomy; 5d) Avoid Argumentation; 5e) Roll with Resistance; 5f) Develop Discrepancy; 5g) Support Self-Efficacy; 6) Complete Personal Quit Plan; 6a) Reasons for Quitting; 6b) 5 Step for Quitting: Get Ready, Get Support, Learn New Skills & Behaviors; Get Cessation Treatment; Be Prepared; 7) Quitting Resources; and 8) Follow-Up.

Prior to recruitment at each site, the study chiropractor will complete a baseline tobacco cessation survey and then again 1 year after recruitment has started at their site. (Survey is in Appendix C.)

Tobacco Cessation Intervention

Volunteers in the chiropractic treatment group will receive the "5As" tobacco cessation program as adapted for chiropractic clinics.[74] It is designed to be brief and fit into clinic patient flow. The program will be modified slightly to fit into this randomized trial.

Ask: The volunteers will be asked about their tobacco use status at each visit by the treating chiropractor. The questions will include the baseline questions from the baseline instrument.

Advise: The chiropractor will discuss health risks of using tobacco. S/he will emphasize how tobacco affects the volunteers' low back condition and other health problems they may have such as chronic pain, decreased healing, respiratory conditions, and allergies. The chiropractor will then advise the volunteer to quit, and will be direct and non-judgmental, as well as acknowledging the difficulty of quitting.

Assess: The chiropractor will ask a series of questions about the readiness to quit. These questions will include the "5 Rs" of motivational interviewing: relevance, risks, rewards, roadblocks, and repetition. Reasons for quitting must be made personally relevant. The volunteers must identify risks and rewards for themselves. Personal obstacles will be identified. The chiropractor will express empathy, promote patient autonomy, and support self-efficacy.

Assist: The chiropractor will assist the volunteer in completing a personal quit plan with quit date. The chiropractor will work with the volunteer on a five-step plan for quitting (get ready, get support and encouragement, learn new skills and behaviors, get cessation treatment, and be prepared for difficulties), disseminate tobacco cessation resources, and make necessary referrals. This discussion will include pharmacotherapy options (prescription and nicotine patches, natural methods (e.g., exercise and relaxation), and web-based quitting programs. All volunteers in the program will be given a volunteer packet with motivational cessation information.

Arrange: Study investigators will arrange follow-up to determine adherence to the program, level of motivation to quit and program success. This information will be gathered at each follow up visit.

Ending Care

Volunteers in either care group may at some time during the six-week period have severe exacerbations that may require referral for surgery or other specialty care. Criteria for ending care may include the presence of any exclusion criteria, worsening pain, or loss of function. The chiropractor will have the responsibility to end care for participants in the CMT plus conventional medical care (CMC) group, while the primary care provider will provide this service to the CMC group. Any volunteer who must change care due to referral will remain in the study and for statistical purposes will be included in his/her original group for analysis. All attempts will be made to obtain outcome measures at all collection points regardless of whether participants change care (intention-to-treat analysis).

Figure 6. Follow-up reminders (See Appendix D)

E-mail follow-up reminder:
Thank you for your continued participation in the ACT low back pain study. As a reminder we ask that you fill out assessments at week 2, 4, 6, and month 3. It is time for your week xx assessment. Please complete this by XX at https://backtoaction. If you have any questions, please contact me at XX (site manager's contact information).
Thank you,
Site PM (to be determined)
Reminder: Your login is your email address.

Text follow-up reminder:
Your ACT assessment for week XX is ready to be completed. Please go to www.backtoaction.com and complete by XX. (provide date of one week later)
Thank you, Site PM

5.3 Primary Outcomes

Demographics

The following information will be collected at baseline after receipt of written informed consent: Date of birth, gender, ethnicity, race, marital status.

Low Back Pain Variables (Appendix A)

Numerical Rating Scale (NRS) (primary endpoint)

Volunteers will be asked to rate their level of pain on that day on an ordinal 11-box scale (0=no LBP; 10=worst LBP possible) at baseline and at all of the follow-up assessments. The NRS has excellent metric properties, is easy to administer and score, and has received much use in LBP research.[79,80] Pain data will be collected at baseline and at all endpoint visits. The question will capture information pertaining to pain over the last 24 hours.

Roland Morris Disability Questionnaire (RMDQ) (primary endpoint)

As in previous LPB studies, the volunteer self-report[81] modified 24-item version of the RMDQ will be used to assess LBP-related disability. The RMDQ may be the most common and respected LBP assessment instrument in LBP outcomes research.[82] It is a one-page questionnaire related to LBP disability with documented reliability and validity.[83] It can discriminate between different forms of treatment for back pain, and it is sensitive to clinical change.[81,84,85] The RMDQ has been chosen for a number of clinical trials of LBP treatments for its excellent metric properties, ease of use, patient acceptance, and high face validity. This questionnaire will be administered at baseline and at all endpoint visits.

Bothersomeness of Symptoms

The bothersomeness of symptoms commonly associated with LBP will be measured using an existing measure from the LBP literature. Volunteers will be asked to rate how bothersome various aspects of their LBP have been during the past week, each symptom measured on a 1 to 5 scale (where 1=not at all bothersome and 5=extremely bothersome). A LBP bothersomeness index will be calculated by summing the four symptom ratings (1-20). Bothersomeness questions are practical and have demonstrated good internal consistency, construct validity, and responsiveness to change with time in patients with LBP and sciatica.[86] Bothersomeness will be measured at baseline and at all endpoint visits.

Back Pain Functional Scale (BPFS)

The Back Pain Functional Scale is a 12-question functional status survey designed for use as an individual patient decision-making tool. Each of the 12 questions is answered using a 5-point Likert-type scale and therefore scores for this scale will range from 0-60.[85] In recent studies, the BPFS is showing more improved sensitivity to change than the RMDQ. This scale will be administered at baseline and all endpoint visits.

Patient Expectation

Previous work has shown that patient expectation regarding benefit of care can be a significant non-specific effect.[87] Two questions regarding patient expectation of benefit from CMT and their general expectation of improvement in 1 month were modified for this study. Patient expectation will be assessed at the baseline visit only.

Patient Satisfaction

A one item patient satisfaction questionnaire was developed based on the work of Cherkin et al.[50] This will be administered at week 2, week 4 and week 6 of care.

Global Improvement Scale

This is a modification of the Visual Analog Scale (VAS) developed to assess degree of improvement over a specified period of time. It will be administered at week 6 and month 3.

Medication Use

Based upon the pilot study conducted at Ft Bliss, volunteers will most likely have been prescribed pain medication by a primary care provider prior to being enrolled in the study. At baseline and at all endpoint visits, we will collect data on the types of medication used and frequency of use.

Duty Status

Volunteers will be asked about their duty status (full, light, limited) at their baseline interview. Change to that status will be questioned at each assessment.

Tobacco Cessation Variables

Tobacco Use

Volunteers will be asked about their tobacco use with the questionnaire by Gordon et al.[74,78] The 7-day abstinence will be determined from the point-prevalence of tobacco use: "Have you smoked, even a puff, in the last 7 days?" Prolonged abstinence will be defined as no tobacco use in the prior 6 months. Other variables will include annual quit attempts, number of cigarettes smoked per day, extent of current tobacco use, and current readiness to quit.

Tobacco Dependence

The level of tobacco dependence will be evaluated as in Fagerstrom & Schneider.[88] We will ask how long the volunteer has smoked, how soon after waking the first cigarette is smoked, and if there are strong cravings when going two hours without a cigarette.

5.3.1 Data Analysis

The analysis team will conduct the data analyses using SAS System for Windows (Release 9.2). They will collaborate with the investigators in presenting and interpreting the results. Descriptive statistics of participant baseline characteristics will be presented for each treatment group to assess their comparability as well as the generalizability of the sample. Descriptive statistics of the primary and secondary outcome variables will be presented for each treatment group at baseline, weeks 2, 4, 6 and month 3.

The primary outcome analysis will focus on the changes from baseline to week 6 since this is the length of the chiropractic care group. Similar analyses will be conducted including the 2 week, 4 week and 3 month waves. Outcome measures will be compared between the chiropractic and medical care only groups at baseline to check for imbalances in the randomization.

A difference-of-differences approach will be used to compare changes over time in the chiropractic arm to changes over time in the conventional medical care only arm. This will be implemented as a regression model rather than by literally modeling change scores. Each study participant will contribute an observation for each wave of data collection. The regression models can be ordinary, logistic, ordered logistic or Poisson depending on the distribution of the outcome measure. As an example we present

the logistic version of the model that might be used for a simple satisfied vs. not satisfied survey response.

Model 1. Pre-Post Comparison of Satisfaction between Chiropractic and Medical Care

$$\ln\left(\frac{p_{ijt}}{1-p_{ijt}}\right) = \beta_0 + \beta_1\tau_1 + \vec{\beta}_2\vec{x}_{ijt}$$

Where p_{ijt} is the probability participant *i* responds as satisfied in treatment arm *j* in time period *t*. *t* takes on 2 values, 0 or 1, for the baseline and 6 week study periods in the basic model. The τ_1 takes on the value 1 in the chiropractic treatment arm at week 6 and takes on the value zero in the medical care arm. τ_1 takes on the value of zero in both treatment arms in the baseline wave. β_1 is an estimate of the chiropractic treatment effect. This model can be easily modified to reflect multiple post time periods (e.g., weeks 2, 4, and month 3). Participant level random effects can be included to control for clustering within participant over time. X can include other patient level covariates to control for imbalance in randomization and differential attrition. It may also be fit for a single outcome type or simultaneously fit across several outcomes to accommodate the correlations between outcomes within participant. Adjusted mean differences and 95% confidence intervals between conventional medical care alone and conventional medical care plus CMT at week 6 will be based on the final models. An intention-to-treat analysis will be used.

5.3.2 Sample Size

Pain Sample Size

The study power is primarily driven by the requirement to detect a practically important average difference between treatment and control groups of 2.0 in the RMDQ. A sample size of 250 (100 treatment and 100 medical care after 20% attrition) will produce a power of 80% at the 5% level for a 2-sided test for this difference. At this sample size the power to detect a practically important difference of 1.0 for the NRS is 92%. This sample size will also have 80% power for a difference of 4.4 (less than half a population standard deviation) in the BPF scale. For a dichotomous self-reported satisfaction measure the power will be 80% to detect a difference of 20% (e.g. 40% for chiropractic participants vs. 20% for medical care.)

Tobacco Cessation Sample Size

A conservative estimate of the subset of the study population that smokes is 30%. Two measures of tobacco use reduction will be considered, 7 day abstinence and prolonged abstinence. With a sample size of 75 (30 treatment and 30 medical care after 20% attrition), we will have 80% power at the 5% level for a difference in 7 day abstinence of 35% (e.g., 9% vs. 44%). We will have 80% power at the 5% level for a difference in prolonged abstinence of 37% (e.g., 13% vs. 50%). We will have more power for similar comparisons when combining sites. After adjusting for clustering within sites a combined multi-site estimate would have 80% power to detect differences of 14% for 7 day abstinence and 16% for prolonged abstinence.

5.4 Reporting of Protocol Deviations

All protocol deviations will be tracked and submitted to the Palmer DSMC and reported to study IRBs per respective reporting requirements. It is important to note that any deviation to the protocol that may have an effect on the safety or rights of the volunteer, or the integrity of the study will be promptly reported to the Palmer College of Chiropractic Human Protections Officer within 24 hours of becoming aware of the deviation.

In this study, protocol deviations will be tracked on the web system by all personnel. Necessary information needed to complete the form includes: date of occurrence, participants involved (options include: all, several, one, none), and a notes field for specific details. After submission an automatic email notification will be sent to the lead and site project manager. The site project manager will have primary responsibility for accessing the protocol deviation report and updating the submission with the appropriate category and notes.

5.5 Data Monitoring

5.5.1 Data Management

The Clinical Trial Coordinating Center (CTCC) within the Palmer Center for Chiropractic Research has: 1)developed web-based data collection forms; 2) program web applications to support data and project management; 3)will continue to provide technical support; and 4) execute procedures for data security and data quality control, storage and back-up. The programmer has designed the web applications and database structures based on Palmer Center for Chiropractic Research standards. He has programed the ASP.NET web application elements in C# and Structured Query Language (SQL) using Microsoft Visual Studio 2010 and Microsoft SQL Server Enterprise Manager. Web application modules include patient baseline screening questionnaires and follow-up data collection, patient eligibility checks, random treatment assignment, participant tracking, report generation and follow-up scheduling. Data entry interfaces are programmed with appropriate participant flow restrictions, validation schemes and skip patterns. The CTCC uses a Project/Users Permissions System (PUPS) to control project personnel access to web modules. All data are stored on a secure internal Microsoft SQL Server. The system was developed and tested by data-related CTCC personnel on a development server and then published to the training site for further testing and training by other CTCC personnel. After testing was complete the site was published to the official project site that resides on a production web server secured with Secure Socket Layers (SSL) certified by Thawte Server CA. The servers are all maintained by Information Services, Palmer College of Chiropractic. All project systems are integrated with the Central Patient Database (CPD) and use a PUPS to control project personnel access to web modules. All data are stored on an internal Microsoft SQL Server to which only the Data Core Manager, Data Manager, Web Application Architect and Web Programmer have access via a Microsoft SQL Server Enterprise Manager interface. The CTCC manager will monitor the quality of the web applications and related databases, manage change requests and create documentation, and assisted by the Web Programmer.

All participant questionnaires will be administered via the web. Baseline screening will be performed on the site PM’s computer. Follow-up questionnaires can be completed at any computer available to the

participant. During the baseline screening, the participant will be asked to create a username/email and password that can be used to provide secure access on follow-up questionnaires. Forgotten passwords will be emailed to their personal email account. In the event of a forgotten username, the participant will be asked to contact their site project manager. During baseline screening, the participant will also be required to provide email and cell phone information. Follow-up contact will be made using email, cell phone voicemail, and/or text messaging.

Web reports detailing when follow-up contacts need to be made with each participant will be made available to the lead and site project managers. The web system is also programmed to send out a reminder email to each participant when study assessment becomes available (date that data collection window opens for that study assessment) as well as when the online assessment is actually due if the participant has not completed the assessment by the due date. Site PMs may also contact study participants by email, text message, and/or phone call for additional reminders if needed. (See Appendix D for details)

Data management and quality control of web forms are performed using SQL views, stored procedures and real-time, web-based reports. Automated reports are viewed by the Data Manager and Project Managers to determine if quality improvement actions such as improved documentation, protocol revisions or personnel retraining.

Final project datasets are assembled by transferring data from Microsoft SQL Server to SAS System for Windows (Release 9.1). The Data Core Manager writes and tests SAS programs to create datasets as requested by the investigators and creates the data dictionaries. Database management system copy is used to move data across software applications.

5.5.2 Data and Safety Monitoring Plan.

Participant Safety Monitoring Plan: See Reporting of Adverse Events.

Data Monitoring Plan: *Data Collection and Management*

Information is collected at every stage of recruitment, treatment allocation, and throughout treatment, so that patient flow can be reported according to the CONsolidated Standards of Reporting Trials (CONSORT) guidelines.[89] Specifically, we collect recruitment source, total number of responses per recruitment source, potential participants' resolution (i.e., ineligible, do not wish to participate, allocated), the number allocated to each treatment group, participant compliance to treatment protocol, the number lost to follow-up, and the number of participants completing the trial. All self-report questionnaires are web based and password protected. They are stored in a secure database at the CTCC. Site PMs have oversight for all data collection.

The project's web system is password-protected and uses a Microsoft SQL Server database platform to store all data. Study personnel have unique user IDs and passwords restricting access from a Main Menu. All data collected by study personnel are recorded in user-friendly data-entry interfaces. The CTCC data manager creates the data dictionaries and datasets for analysis.

Quality control procedures are utilized to ensure that recruitment is on schedule, treatment allocation is occurring as planned, data collection protocols are being used accurately, data collected through the Computer Assisted Telephone Interview (CATI) and other web interfaces are being stored correctly in the SQL databases, and that the data are being transferred and retrieved properly.

Data Storage

All Informed Consent Documents/HIPAA and all paper data collection forms used will only include unique study ID numbers or participant name, be accessible only by study personnel, and kept in locked filing cabinets. Treatment and Exam forms will be kept in the locked cabinet. After information has been entered into the database, it will be stored in the locked cabinet for up to 7 years after the study has been completed. Computer files with volunteer names will be password protected with restricted access to project staff who will only use this information to recruit volunteers and obtain follow-up data. All analytic data files and tracking databases will be maintained on a secure, password-protected server at the Clinical Trial Coordinating Center (CTCC)**.**

Data Transfer

Electronic data are collected via web forms on a web server secured with Secure Socket Layers (SSL) certified by ipsCS CLASEA1 Certification Authority, www.ipsCS.com. All data are stored on an internal Microsoft SQL Server to which only the CTCC Data Manager and Database Programmer have direct access. All web forms and reports have limited accessibility based on individual project role.

Once data have been verified, cleaned, and de-identified by the Data Core Manager, these data will be a passed for analysis to RAND Corporation via encrypted password-protected file transfer (secured FTP). All RAND research that involves acquisition of private, individual-level data is required to follow the common federal rule for the protection of human subjects of research. These guidelines are spelled out in 45 CFR 46 and in RAND's Multiple Project Assurance of Compliance with the regulations. The Assurance of Compliance is on file with the Department of Health and Human Services and also serves as our assurance of compliance with the regulations of other federal departments and agencies.

It is not possible for all study data to remain anonymous. However, to protect confidentiality, paper data will be kept in locked filing cabinets and will be identifiable only by unique study ID numbers. Computer files will be password protected with access restricted to staff who will use this information only to recruit volunteers or obtain follow-up data. All web based questionnaires and data files are password protected and stored in a secure database at the CTCC.

CTCC Data Security and Confidentiality

The CTCC computer servers are stored in locked, temperature-controlled rooms with the other institutional servers at Palmer College. Back-up tapes of the network drive are produced nightly. Palmer College uses Symantec Backup Exec and FalconStor for server backups. Tapes are handled solely by network staff, primarily by one person. Backup tapes are transported daily to a storage vault in a

building designated for this purpose. The vault is used exclusively for backup tape storage and the door requires both a key and combination for access.

5.5.3 Compliance and Co-Interventions

Non-compliance with treatment protocols is a potential confounder of outcomes in clinical trials. Participants are asked on each questionnaire about any co-interventions, including care from providers outside the study and the use of medications or other self-administered treatments. Individual site tracking logs were created to monitor participant's compliance with respect to completing assessments within the allotted time period (see Appendix D). In addition, another log is utilized to ensure data is collected at all study chiropractic treatment visits.

5.6 Reporting of Adverse Events

AE Collection

For this study an Adverse Event (AE) will be defined as any untoward medical occurrence that may present itself during the conduct of the study and that may or may not have a causal relationship with the study procedures. AEs will be monitored at three levels: 1) self-report AE collected at Week 2, Week 4, and Week 6 assessments or via self-report to the study physician or site PM directly; 2) serious adverse events (SAE) regardless of their attribution reported via Week 2, Week 4, Week 6 or Month 3 assessments or via self-report to the study physician or site PM directly; and 3) Unanticipated Problems Involving Risks to Subjects or Others (UPIRTSOs) that are unanticipated, serious, and at least possibly related to the research procedures that do not meet the SAE definition also reported directly to the site PM. Each allocated participant will be given a business card with the PMs' contact information as well as instructions for when it is important to contact the PM. Additionally, contact information for the site PM as well as the PI are listed in the informed consent document.

AE Reporting

Since the majority of AEs will be reported via the participant at the online assessments, a protocol was developed to ensure oversight of this data is being maintained and that AEs meeting site IRB reporting requirements are conveyed to the site PM/PI contemporaneously. A designated study clinician will be assigned to review a live report of AEs reported from the 'Reactions and discomforts' section of the participant online assessment. This portion of the assessment is designed to illicit adverse events during the time period from the last online assessment (see Appendix A). These questions have been added to study assessments at week 2, week 4, and week 6. Additionally, participants are told during the informed consent process about the importance of reporting adverse experiences and instructions for how to report these experiences.

The designated study clinician will convey these events to the site PM for appropriate reporting to IRB. The study clinician may also ask that the site PM contact the participant if more information is needed regarding a reported adverse experience that could be potentially serious, appear to have no resolution date, or appear to require additional medical follow-up for safety purposes. Our goal is to ensure that

we are following up with any event that could appear to affect participant safety and report adverse events per all study IRB reporting guidelines.

We will use the FDA definition of SAE, which is any adverse experience occurring during treatment that results in any of the following outcomes: death, a life-threatening adverse experience, in patient hospitalization or prolongation of existing hospitalization, a persistent or significant disability/incapacity, or a congenital anomaly/birth defect.

All SAEs and UPIRTSOs will be reported to the IRBs (Palmer and RAND), Palmer DSMC, and the United States Army Medical Research and Material Command, Office of Research Protections (USAMRMC ORP) within 5 business days. The site PM will be responsible for reporting all AEs to the lead PM at Palmer. The lead PM will report to Palmer IRB, Palmer DSMC and to RAND PM. The RAND PM will report to RAND IRB and USAMRMC ORP.

UPIRTSOs, SAEs related to participation in the study, and all volunteer deaths related to participation in the study will be promptly reported by phone (301-619-2165), by email (hsrrb@amedd.army.mil), or by facsimile (301-619-7803) to the USAMRMC ORP, Human Research Protections Office (HRPO). A complete written report should follow the initial notification. In addition to the methods above, the complete report can be sent to the US Army Medical Research and Materiel Command, ATTN: MCMR-ZB-P, 504 Scott Street, Fort Detrick, Maryland 21702-5012.

The medical monitor is required to review all UPRIRTSOs involving risk to volunteers or others, SAEs, and all volunteer deaths associated with the protocol, and provide an unbiased written report of the event to the USAMRMC Office of Research Protections (ORP), HRPO. At a minimum, the medical monitor will comment on the outcomes of the event or problem, and in the case of a SAE or death, comment on the relationship to participation in the study. The medical monitor will also indicate whether he/she concurs with the details of the report provided by the study investigator. Reports for events determined by either the investigator or medical monitor to be possibly or definitely related to participation, and reports of events resulting in death will be promptly forwarded to the HRPO.

All study protocol violations will be reported to the Palmer DSMC. Protocol violations meeting respective study site's IRB criteria for reporting will also be reported per IRB guidelines.

Qualitative Analysis and Quality Assurance

Site visits will be conducted to ensure the rigor and robustness of the trials. This oversight will provide quality control and feedback to the Palmer Center for Chiropractic Research and to DoD for implementation of future research programs. In addition, because it is a multi- site study in which the intervention will vary from context to context, by using both quantitative and qualitative data, we can systematically describe each context to determine how they are similar and different from each other and to determine to what degree (if any) these similarities and differences might affect the effectiveness of the intervention being studied. Robustness is the confidence that an intervention will perform (within a given range) similarly across different contexts. In this study, programs must be successfully implemented across widely varying military contexts.

The approach to be used is akin to fidelity evaluation and is a practice- level method that serves purposes similar to treatment adherence in clinical studies. Fidelity measurement assesses integrity of initial and on- going implementation (i.e., is the practice model being delivered according to design). How faithfully an intervention is implemented as it was planned affects how well it succeeds and how reliably it can be adapted to other settings.

Fidelity is assessed by a process evaluation tailored to the practice and can involve multiple data sources including questionnaires, administrative records, and qualitative observations. The method to be used will be to interview key informants who have been involved at the site (in this instance WRNMMC) with the chiropractic trials either in the implementation, administration or in the running of the trials. We have identified 7 key informants (potential participants) to be interviewed such as local leadership, providers, and study staff.

Potential participants will be invited to take part in this qualitative portion of the ACT 1 study. Research staff will review the informed consent document with each partipant emphasizing the purpose of the qualitative piece, and potential information to be gained by participating in this study. Research staff will also emphasize that participation in this piece is voluntary and that refusal to participate will not have any implications. The interview will begin after the informed consent document is signed and all participant questions are answered to satisfaction.

Standard probes, such as verification and compare and contrast questions will be used. These questions provide more details about the topic being discussed and usually generate lists of items, short qualitative answers, and close-ended, quantitative data. We will follow well-established procedures for conducting semi-structured interviews.

Quality Assurance

The Clinical Trial Coordinating Center will use their standard Quality Assurance / Quality Control (QA/QC) processes. Their focus will be on the recruitment process and treatment protocols, looking at the application of both the eligibility and exclusion criteria. Initial questions during the site visits will glean information as to whether randomization occurred and was followed. We will also examine the administration of the trials including the management of Informed Consent Documents, HIPAA forms, adverse events and data transfer. Overall we are interested in fidelity to the treatment protocol and identifying challenges to maintaining standardized practice and evaluation methods.

These indicators will be part of the chart review:

- Informed consent forms and processes
- Eligibility criteria
- Missed visits
- Concomitant therapies
- SAE identification and reporting
- Treatment and procedure protocols
- Protocol endpoint identification

Additional QA for the trial includes:

- Monitoring all clinical research training certifications and healthcare licensures of all study personnel
- Protocol audits
- Data Management QC procedures
- Tracking & Monitoring protocol deviations

6.0 TIMELINE

Table 2. Timeline for Study I (ACT1) Controlled Trial for Low Back Pain and Tobacco Cessation

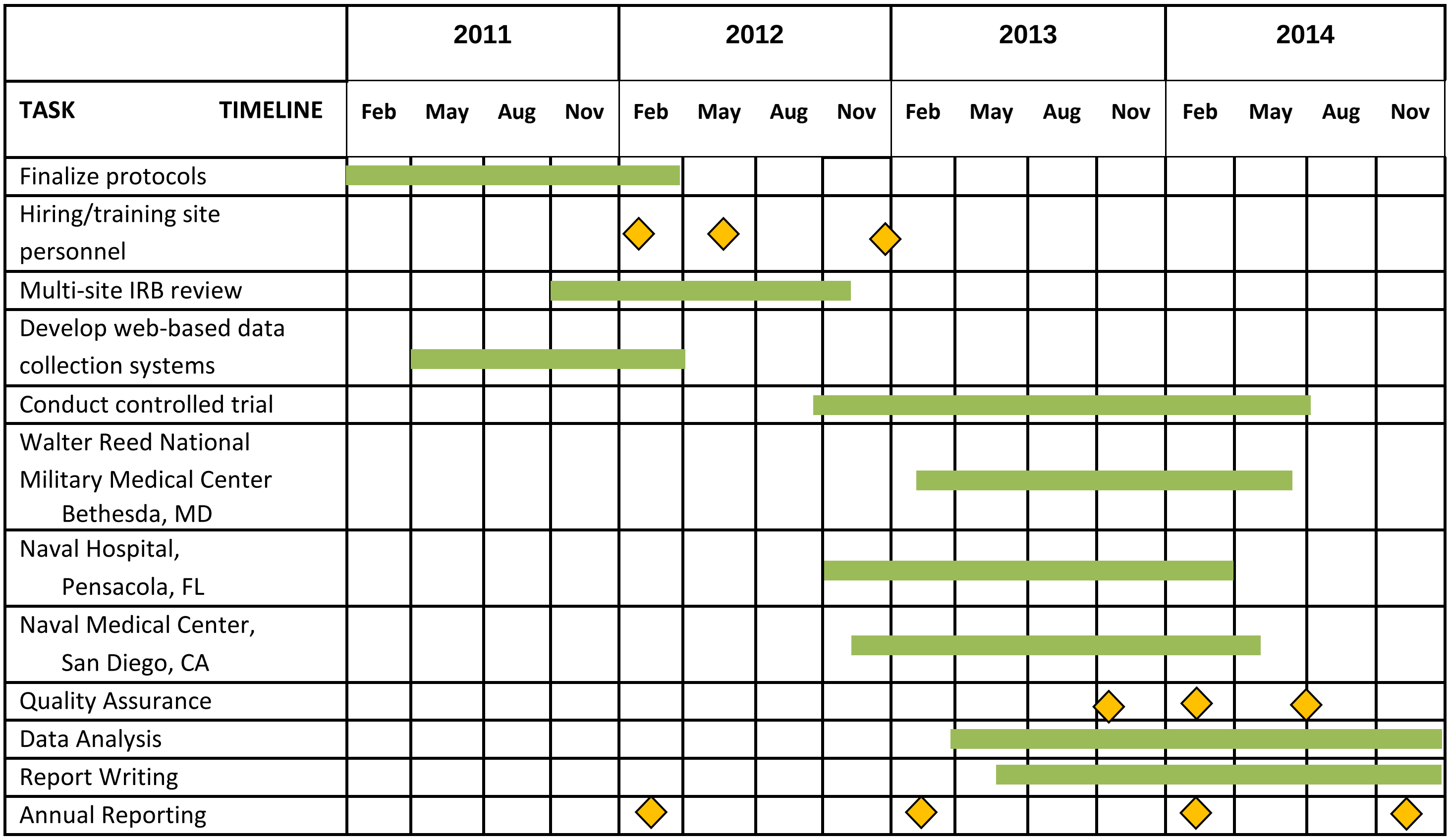

References

Reference List

1. Carey TS, Freburger JK, Holmes GM, Castel L, Darter J, Agans R, et al. A long way to go: practice patterns and evidence in chronic low back pain care. *Spine.* 2009;34:718-724.

2. Nachemson AL, Jonsson E. Neck and Back Pain. Philadelphia: Lippincott Williams & Wilkins; 2000.

3. Borkan JM, Koes B, Reis S, Cherkin DC. A report from the Second International Forum for Primary Care Research on Low Back Pain. Reexamining priorities. *Spine (Phila Pa 1976).* 1998;23:1992-1996.

4. Shekelle PG, Markovich M, Louie R. Factors associated with choosing a chiropractor for episodes of back pain care. *Med Care.* 1995;33:842-850.

5. Barnes PM, Bloom B, Nahin RL. Complementary and alternative medicine use among adults and children: United States, 2007. *Natl Health Stat Report.* 2008;1-23.

6. Walker BF. The prevalence of low back pain: a systematic review of the literature from 1966 to 1998. *J Spinal Disord.* 2000;13:205-217.

7. Webster BS, Snook SH. The cost of 1989 workers' compensation low back pain claims. *Spine.* 1994;19:1111-1115.

8. Anderson R, Meeker WC, Wirick BE, Mootz RD, Kirk DH, Adams A. A meta-analysis of clinical trials of spinal manipulation. *J Manipulative Physiol Ther.* 1992;15:181-194.

9. Cohen SP, Nguyen C, Kapoor SG, Anderson-Barnes VC, Foster L, Shields C, et al. Back pain during war: an analysis of factors affecting outcome. *Arch Intern Med.* 2009;169:1916-1923.

10. Feuerstein M, Berkowitz SM, Peck CA, Jr. Musculoskeletal-related disability in US Army personnel: prevalence, gender, and military occupational specialties. *J Occup Environ Med.* 1997;39:68-78.

11. Jones BH, Hanson BC. Injuries in the Military: A Hidden Epidemic. A Report by the Armed Forces Epidemiology Board Injury Work Group. Aberdeen Proving Ground, MD: U.S. Army Center for Health Promotion and Preventive Medicine.; 2010.

12. Lincoln AE, Smith GS, Amoroso PJ, Bell NS. The natural history and risk factors of musculoskeletal conditions resulting in disability among US Army personnel. *Work.* 2002;18:99-113.

13. Triano JJ. The Mechanics of Spinal Manipulation. In: Herzog W, editor. Clinical Biomechanics of Spinal Manipulation.New York: Churchill Livingstone; 2000. 92-190.

14. Herzog W. The Mechanical Neuromuscular, and Physiologic Effects Produced by Spinal Manipulation. In: Herzong W, editor. Clinical Biomechanics of Spinal Manipulation.New York: Churchill Livingstone; 2000. 191-207.

15. Triano JJ. Biomechanical Analysis of Motions and Loads During Spinal Manipulation University of Michigan; 1998.

16. Gudavalli MR, Triano JJ. An analytical model of lumbar motion segment in flexion. *J Manipulative Physiol Ther.* 1999;22:201-208.

17. Herzog W. Torque: an appraisal of misuse of terminology in chiropractic literature and technique. *J Manipulative Physiol Ther.* 2000;23:298-299.

18. Haldeman S, Chapman-Smith D, Petersen DM, Jr. Guidelines for chiropractic quality assurance and practice parameters: proceedings of the Mercy Center Consensus Conference. *Aspen Publishers, Inc.* 1992.

19. Evans DW. Mechanisms and effects of spinal high-velocity, low-amplitude thrust manipulation: Previous theories. *JMPT.* 2002;25:251-262.

20. Andersson GB. Epidemiological features of chronic low-back pain. *Lancet.* 1999;354:581-585.

21. Budgell B. Spinal Manipulative Therapy and Visceral Disorders. *Chiropractic Journal of Australia.* 1999;29:123-128.

22. Pickar JG. Neurophysiological effects of spinal manipulation. *Spine J.* 2002;2:357-371.

23. Greenman PE. Principles of Manual Medicine. Baltimore: Williams & Wilkins; 1989.

24. Brennan PC. Review of the Systemic Effects of Spinal Manipulation. In: Gatterman MI, editor. Foundations of chiropractic. 1 ed. St. Louis: Mosby; 1995. 267-278.

25. Farfan HF. The Scientific Basis of Manipulation Procedures. In: Buchanan WW, Kahn MF, Laine V, Rodnan GP, Scott JT, Zvaifler NJ, et al., editors. Clinics in rheumatic diseases.London: W.B. Saunders Company Ltd.; 1980. 159-177.

26. Giles LGF. Anatomical Basis of Low Back Pain. Baltimore: Williams & Wilkins; 1989.

27. Vernon H. Basic Scientific Evidence for Chiropractic Subluxation. In: Gatterman MI, editor. Foundations of Chiropractic: Subluxation.New York: Mosby; 1997. 35-55.

28. Lewit K. Manipulative Therapy in Rehabilitation of the Locomotor System. Oxford: Butterworth-Heinemann; 1991.

29. Haldeman S. The Clinical Basis for Discussion of Mechanisms of Manipulative Therapy. In: Korr IM, editor. The neurobiologic mechanisms in manipulative therapy.NY: Plenum Press; 1978. 53-75.

30. Vernon H. Biological rationale for possible benefits of spinal manipulation. Cherkin DC, Mootz RD, editors. AHCPR Publication No. 98-N002, 105-115. 1997. Chiropractic in the United States: Training, Practice and Research.

Ref Type: Case

31. Henderson CNR. Three Neurophysiologic Theories on the Chiropractic Subluxation. In: Gatterman MI, editor. Foundations of chiropractic: Subluxation. 2 ed. St. Louis: Mosby; 2005. 296-303.

32. Denslow JS. An analysis of the variability of spinal reflex thresholds. *J Neurophysiol.* 1944;7:207-215.

33. Goldstein M. The research status of spinal manipulative therapy: NINCDS monograph No. 15. Bethesda: U.S. Department of Health, Education, and Welfare; 1975.

34. Assendelft W, Koes BW, Knipschild PG, Bouter LM. The relationship between methodological quality and conclusions in reviews of spinal manipulation. *JAMA.* 1995;274:1942-1948.

35. Lawrence DJ, Meeker W, Branson R, Bronfort G, Cates JR, Haas M, et al. Chiropractic management of low back pain and low back-related leg complaints: a literature synthesis. *J Manipulative Physiol Ther.* 2008;31:659-674.

36. Bronfort G, Haas M, Evans R, Leininger B, Triano J. Effectiveness of manual therapies: the UK evidence report. *Chiropr Osteopat.* 2010;18:3.

37. Bronfort G, Haas M, Evans R, Kawchuk G, Dagenais S. Evidence-informed management of chronic low back pain with spinal manipulation and mobilization. *Spine J.* 2008;8:213-225.

38. Dagenais S, Tricco AC, Haldeman S. Synthesis of recommendations for the assessment and management of low back pain from recent clinical practice guidelines. *Spine J.* 2010;10:514-529.

39. Furlan AD, Clarke J, Esmail R, Sinclair S, Irvin E, Bombardier C. A critical review of reviews on the treatment of chronic low back pain. *Spine (Phila Pa 1976).* 2001;26:E155-E162.

40. Walker BF, French SD, Grant W, Green S. Combined chiropractic interventions for low-back pain. *Cochrane Database Syst Rev.* 2010;4:CD005427.

41. Ferreira ML, Ferreira PH, Latimer J, Herbert R, Maher CG. Does spinal manipulative therapy help people with chronic low back pain? *Aust J Physiother.* 2002;48:277-284.

42. Haigh R, Clarke AK. Effectiveness of rehabilitation for spinal pain. *Clin Rehabil.* 1999;13 Suppl 1:63-81.:63-81.

43. Shekelle PG, Adams AH, Chassin MR, Hurwitz EL, Brook RH. Spinal manipulation for low-back pain. *Ann Intern Med.* 1992;117:590-598.

44. Cooper RA, McKee HJ. Chiropractic in the United States: trends and issues. *Milbank Q.* 2003;81:107-38, table.

45. Hawk C, Long CR, Boulanger K, Morschhauser E, Fuhr A. Chiropractic care for patients aged 55 years and older: report from a practice-based research program. *J Am Geriatr Soc.* 2000;48:534-545.

46. Christensen M.G., Kerkhoff D, Kollasch M.W., Cohn L. Job analysis of chiropractic. Greeley, CO: National Board of Chiropractic Examiners; 2000.

47. Barnes PM, Powell-Griner E, McFann K, Nahin RL. Complementary and alternative medicine use among adults: United States, 2002. *Adv Data.* 2004;1-19.

48. Wolsko PM, Eisenberg DM, Davis RB, Kessler R, Phillips RS. Patterns and perceptions of care for treatment of back and neck pain: results of a national survey. *Spine (Phila Pa 1976).* 2003;28:292-297.

49. Carey TS, Garrett J, Jackman A, McLaughlin C, Fryer J, Smucker DR. The outcomes and costs of care for acute low back pain among patients seen by primary care practitioners, chiropractors, and orthopedic surgeons. The North Carolina Back Pain Project. *N Engl J Med.* 1995;333:913-917.

50. Cherkin DC, MacCornack FA. Patient evaluations of low back pain care from family physicians and chiropractors. *West J Med.* 1989;150:351-355.

51. Sox HC, Greenfield S. Comparative effectiveness research: a report from the Institute of Medicine. *Ann Intern Med.* 2009;151:203-205.

52. Conway PH, Clancy C. Comparative-effectiveness research--implications of the Federal Coordinating Council's report. *N Engl J Med.* 2009;361:328-330.

53. Schwartz D, Lellouch J. Explanatory and pragmatic attitudes in therapeutical trials. *J Chronic Dis.* 1967;20:637-648.

54. Zwarenstein M, Treweek S. What kind of randomized trials do we need? *CMAJ.* 2009;180:998-1000.

55. Luce BR, Kramer JM, Goodman SN, Connor JT, Tunis S, Whicher D, et al. Rethinking randomized clinical trials for comparative effectiveness research: the need for transformational change. *Ann Intern Med.* 2009;151:206-209.

56. Karanicolas PJ, Montori VM, Devereaux PJ, Schunemann H, Guyatt GH. A new 'mechanistic-practical" framework for designing and interpreting randomized trials. *J Clin Epidemiol.* 2009;62:479-484.

57. Maclure M. Explaining pragmatic trials to pragmatic policymakers. *J Clin Epidemiol.* 2009;62:476-478.

58. CDC. Annual Smoking-Attributable Mortality, Years of Potential Life Lost, and Productivity Losses-United States 2000-2004. *MMWR.* 2008;57.

59. National Cancer Institute. Changes in Cigarette-Related Disease Risks and Their Implications for Prevention and Control. Bethesda, MD; 1997. Report No.: Vol. 8.

60. Bray RM, Hourani LL, Rae Olmsted KL, et al. 2005 Department of Defense Survey of Health Related Behavious Among Active Duty Military Personnel, A Component of the Defense Lifestyle Assessment Program (DLAP). RTI International; 2006. Report No.: Prepared for the Assistant Secretary of Defense (Health Affairs), U.S. Department of Defense, Cooperative Agreement No. DAMD17-00-2-0057.

61. Dall TM, Zhang Y, Chen YJ, Wagner RC, Hogan PF, Fagan NK, et al. Cost associated with being overweight and with obesity, high alcohol consumption, and tobacco use within the military health system's TRICARE prime-enrolled population. *Am J Health Promot.* 2007;22:120-139.

62. Klesges RC, Haddock CK, Chang CF, Talcott GW, Lando HA. The association of smoking and the cost of military training. *Tob Control.* 2001;10:43-47.

63. Jensen RG. The effect of cigarette smoking on Army Physical Readiness Test performance of enlisted Army medical department personnel. *Mil Med.* 1986;151:83-85.

64. Kao TC, Schneider SJ, Hoffman KJ. Co-occurrence of alcohol, smokeless tobacco, cigarette, and illicit drug use by lower ranking military personnel. *Addict Behav.* 2000;25:253-262.

65. Knapik JJ, Sharp MA, Canham-Chervak M, Hauret K, Patton JF, Jones BH. Risk factors for training-related injuries among men and women in basic combat training. *Med Sci Sports Exerc.* 2001;33:946-954.

66. Altarac M, Gardner JW, Popovich RM, Potter R, Knapik JJ, Jones BH. Cigarette smoking and exercise-related injuries among young men and women. *Am J Prev Med.* 2000;18:96-102.

67. Deyo RA, Bass JE. Lifestyle and low-back pain. The influence of smoking and obesity. *Spine (Phila Pa 1976).* 1989;14:501-506.

68. Goldberg MS, Scott SC, Mayo NE. A review of the association between cigarette smoking and the development of nonspecific back pain and related outcomes. *Spine (Phila Pa 1976).* 2000;25:995-1014.

69. Vogt MT, Hanscom B, Lauerman WC, Kang JD. Influence of smoking on the health status of spinal patients: the National Spine Network database. *Spine (Phila Pa 1976).* 2002;27:313-319.

70. Jamison RN, Stetson BA, Parris WC. The relationship between cigarette smoking and chronic low back pain. *Addict Behav.* 1991;16:103-110.

71. Hawk C, Long CR, Perillo M, Boulanger KT. A survey of US chiropractors on clinical preventive services. *J Manipulative Physiol Ther.* 2004;27:287-298.

72. Rupert RL. A survey of practice patterns and the health promotion and prevention attitudes of US chiropractors. Maintenance care: part I. *J Manipulative Physiol Ther.* 2000;23:1-9.

73. Gordon JS, Istvan J, Haas M. Tobacco Cessation: Attitudes and Behaviors of Oregon Chiropractors. *J Amer Chiro Assoc.* 2005;42:8-14.

74. Gordon JS, Istvan J, Haas M. Tobacco cessation via doctors of chiropractic: results of a feasibility study. *Nicotine Tob Res.* 2010;12:305-308.

75. Liebenson C. Active rehabilitation protocols. In: Liebenson C, editor. Rehabilitation of the spine: a practitioner's manual.Baltimore, MD: Williams and Wilkins; 1996. 355-390.

76. Fiore MC, Bailey WC, Cohen SJ, Dorfman SF, Goldstein MG, Gritz ER, et al. Treating Tobacco Use and Dependence. Clinical Practice Guidelines. Rockville, MD: U.S.Department of Health and Human Services,Public Health Services; 2000.

77. Fiore MC, Jaen CR, Baker TB, Bailey WC, Benowitz NL, Curry SJ, et al. Treating Tobacco Use and Dependence: 2008 Update. Clinical Practice Guideline. Rockville, MD: U.S. Department of Health and Human Services. Public Health Services.; 2008.

78. Gordon JS, Andrews JA, Albert DA, Crews KM, Payne TJ, Severson HH. Tobacco cessation via public dental clinics: results of a randomized trial. *Am J Public Health.* 2010;100:1307-1312.

79. Childs JD, Piva SR, Fritz JM. Responsiveness of the Numeric Pain Rating Scale in Patients with Low Back Pain. *Spine.* 2005;30:1331-1334.

80. van der Roer N, Ostelo RWJG, Bekkering GE, van Tulder MW, de Vet HCW. Minimal Clinically Important Change for Pain Intensity, Functional Status, and General Health Status in Patients with Nonspecific Low Back Pain. *Spine.* 2006;31:578-582.

81. Deyo RA, Cherkin D, Conrad D. The Back Pain Outcome Assessment Team. *Health Serv Res.* 1990;25:733-737.

82. Deyo R. Chiropractic care for back pain. *Health News.* 1998;4:1-2.

83. Roland M, Fairbank J. The Roland-Morris Disability Questionnaire and the Oswestry Disability Questionnaire. *Spine.* 2000;25:3115-3124.

84. Riddle DL. Classification and low back pain: a review of the literature and critical analysis of selected systems. *Phys Ther.* 1998;78:708-737.

85. Stratford PW, Binkley JM, Riddle DL. Development and initial validation of the back pain functional scale. *Spine.* 2000;25:2095-2102.

86. Patrick DL, Deyo RA, Atlas SJ, Singer DE, Chapin A, Keller RB. Assessing health-related quality of life in patients with sciatica. *Spine.* 1995;20:1899-1908.

87. Kalauokalani D, Cherkin DC, Sherman KJ, Koepsell TD, Deyo RA. Lessons from a trial of acupuncture and massage for low back pain: patient expectations and treatment effects. *Spine (Phila Pa 1976).* 2001;26:1418-1424.

88. Fagerstrom KO, Schneider NG. Measuring nicotine dependence: a review of the Fagerstrom Tolerance Questionnaire. *J Behav Med.* 1989;12:159-182.

89. Moher D, Hopewell S, Schulz KF, Montori V, Gotzsche PC, Devereaux PJ, et al. CONSORT 2010 Explanation and Elaboration: Updated guidelines for reporting parallel group randomised trials. *J Clin Epidemiol.* 2010;63:e1-37.

Appendix A. Participant Web-based Study Assessment

DoD-ACT

Baseline 1 Visit Interview

Select Site: Select

Informed Consent: Has the participant signed the informed consent document?

- No
- Yes

If no, specify reason:

Tobacco Cessation Consent: Did the participant consent to be in the tobacco cessation study?

- No
- Yes
- Not Administered
- Not a tobacco user

Next >>

DoD-ACT

Baseline 1 Visit

First name:

Last name:

Middle initial:

Rank:

Cell phone*:

Home phone:

Work phone: ext.

Are you able to receive text messages, in addition to voicemail and email messages?

○ No ○ Yes

Main email*:

Personal email:

Date of birth:

<< Previous Next >>

DoD-ACT

Baseline 1 Visit

1. Duty status:

- ○ Full Duty - no restrictions
- ○ Profiled - limited duty
- ○ Not on Active Duty

2. Sex:

○ Female ○ Male

3. Ethnicity:

- ○ Hispanic or Latino
- ○ Not Hispanic or Latino
- ○ Unspecified

4. Race (mark all that apply):

- ☐ American Indian or Alaska Native
- ☐ Asian
- ☐ Native Hawaiian or Other Pacific Islander
- ☐ Black or African American
- ☐ White
- ☐ Unspecified

<< Previous | Next >>

DoD-ACT

Baseline 1 Visit

1. How did you hear about this research study?

- ○ Acute Care Provider
- ○ Primary Care Provider
- ○ Internal Medicine Provider
- ○ Physical Therapist
- ○ Chiropractor
- ○ Poster
- ○ Word of Mouth
- ○ Other

Specify Other: ____________________

<< Previous   Next >>

DoD-ACT

Baseline 1 Visit

Interview Questions

1. How long ago did your current episode of low back pain begin? Was it...

   An **episode of pain** is defined as at least some low back pain requiring you to seek treatment or modify your activity during consecutive weeks (a week or more with no pain separates episodes).

   - ○ Less than 7 days ago
   - ○ 7 days to less than 16 days ago
   - ○ 16 days to less than 1 month ago
   - ○ 1-3 months ago
   - ○ More than 3 months and less than 6 months ago
   - ○ 6 months to less than 1 year ago
   - ○ 1 year or more ago
   - ○ You are not experiencing a current episode of low back pain

2. Where is your pain currently located? (Mark all that apply)

   - ☐ Low Back
   - ☐ Buttocks
   - ☐ Thigh
   - ☐ Lower Leg

3. Have you received manipulative care from any healthcare provider in the past month?

   ○ No ○ Yes

4. Are you likely to be deployed or receive a distant duty assignment in the next 8 weeks?

   ○ No ○ Yes

5. Do you agree to be enrolled in this clinical trial regardless which treatment group you are assigned to?

   ○ No ○ Yes

6. During this study, you will be asked to read a consent form and complete internet/web-based questionnaires. Do you need assistance from another person to complete these activities?

◯ No ◯ Yes

7. Are you pregnant or planning to become pregnant in the next 8 weeks?

◯ No ◯ Yes

<< Previous   Submit

DoD-ACT

Baseline 1 Exam

Exam Questions

1. Do you suspect the low back pain is caused primarily by a visceral source or systemic condition (e.g., renal disease, endometriosis, MS, malignancy, GI disease)?

   ○ No ○ Yes

2. Is there a condition requiring priority care such that treatment for low back pain should be delayed?

   ○ No ○ Yes

3. Has the participant suffered a spinal fracture within the past 8 weeks?

   ○ No ○ Yes

4. Do you suspect the presence of a spinal or paraspinal infection, inflammatory arthropathy of the spine (i.e. rheumatoid arthritis, ankylosing spondylitis) or severe osteoporosis?

   ○ No ○ Yes ○ Suspected

5. Does the participant exhibit signs of cognitive or memory impairment that compromises your ability to accurately assess health status (e.g. nonsensical statements, not oriented to time and place)?

   ○ No ○ Yes

6. Does this participant need a referral or further testing to rule out pathology that would exclude his/her participation in this study?

   ○ No ○ Yes

7. Does the participant carry a PTSD classification?

   ○ No ○ Yes

8. Notes:

---

9. Treatment Recommendations: (mark all that apply)

- ☐ Prescription Medications
  - ☐ Muscle relaxants
  - ☐ Narcotics
  - ☐ Antidepressants
  - ☐ Anesthetic/Steroid
  - ☐ NSAIDs
- ☐ Referral To
  - ☐ Physical Therapy
  - ☐ Chiropractic
  - ☐ Neurology
  - ☐ Orthopedic
- ☐ Self-Care
  - ☐ OTC Medications
  - ☐ Exercises
  - ☐ Behavior Modification
  - ☐ Other
- ☐ Other

  Specify:

ICD Codes

- ☐ 719.45 - Sacroiliac arthralgia / SI joint arthralgia
- ☐ 721.3 - Lumbosacral spondylosis without myelopathy (facet arthrosis) (facet hypertrophy)
- ☐ 722.93 - Disc disorder of lumbar region, other, unspecified, NOS
- ☐ 722.2 - Discogenic Pain
- ☐ 722.73 - Intervertebral disc disorder with myelopathy lumbar
- ☐ 724.2 - Lumbalgia
- ☐ 724.3 - Sciatica
- ☐ 724.4 - Lumbosacral neuritis or radiculitis
- ☐

724.8 - Lumbar Facet Syndrome (Other symptoms refer to back)
- ☐ 729.1 - Myalgia / myofascial pain
- ☐ 737.2 - Lordosis (acquired) (postural) (hyperlordosis)
- ☐ 739.3 - Nonallopathic lesions of lumbar region NOS
- ☐ 739.4 - Nonallopathic lesions of sacral region NOS
- ☐ 846.1 - Sacroiliac (ligament) sprain
- ☐ 847.2 - Lumbar sprain / strain
- ☐ 847.3 - Sprain of sacrum
- ☐ 847.4 - Sprain of coccyx

CPT Codes

- ☐ 99201 - Problem Focused New Patient Eval.
- ☐ 99202 - Expanded New Patient Eval.
- ☐ 99203 - Detailed New Patient Eval.
- ☐ 99204 - Comprehensive New Patient Eval.
- ☐ 99211 - Est. Patient Eval. – Minimal
- ☐ 99212 - Est. Patient Eval. – Problem Focused
- ☐ 99213 - Est. Patient Eval. – Expanded
- ☐ 99214 - Ext. Patient Eval. - Detailed
- ☐ 20550 - Drain/inject, ligament/cyst
- ☐ 20610 - Drain/inject joint/bursa
- ☐ 96372 - Intramuscular Injection (Dr. supervised)
- ☐ 97010 - Hot/Cold Packs
- ☐ 97014 - Electrical Muscle Stimulation
- ☐ 97035 - Ultrasound
- ☐ 97039 - Mechanical Massage
- ☐ 97110 - Therapeutic Exercise
- ☐ 97112 - Neuromuscular Re-Ed
- ☐ 97124 - Massage
- ☐ 97116 - Gait training therapy
- ☐ 97139 - Physical (unlisted) medicine procedure
- ☐ 97140 - Manual Therapy Technique
- ☐ 97140 - Trigger Point
- ☐ 97530 - Therapeutic Activities
- ☐ 97535 - Self management training
- ☐ 97750 - Physical performance test

DoD-ACT1

SECTION
-
Pain Rating Scales

Select the number that best describes your low back pain at its **worst** during the **past 24 hours**?

No Pain										Worst Possible Pain
○0	○1	○2	○3	○4	○5	○6	○7	○8	○9	○10

Select the number that best describes your **average** low back pain during the **past week**?

No Pain										Worst Possible Pain
○0	○1	○2	○3	○4	○5	○6	○7	○8	○9	○10

Next >>

DoD-ACT 1

Roland Morris Disability Questionnaire

When your back hurts, you may find it difficult to do some of the things you normally do.

This list contains sentences that people have used to describe themselves when they have back pain. When you read them, you may find that some stand out because they describe you **today**. As you read the list, thing of yourself **today**. When you read a sentence that describes how you feel today, choose YES. If the sentence does not describe you, then choose NO.

Remember, only choose YES if you are sure that the sentence describes you **today**.

	No	Yes
I stay home most of the time because of my back.	○	○
I change position frequently to try and get my back comfortable.	○	○
I walk more slowly than usual because of my back.	○	○
Because of my back, I am not doing any jobs that I usually do around the house.	○	○
Because of my back, I use a handrail to get upstairs.	○	○
Because of my back, I lie down to rest more often.	○	○
Because of my back, I have to hold on to something to get out of an easy chair.	○	○
Because of my back, I try to get other people to do things for me.	○	○

Next >>

DoD-ACT 1

Roland Morris Disability Questionnaire

Remember, only choose YES if you are sure that the sentence describes you **today**.

	No	Yes
I get dressed more slowly than usual because of my back.	○	○
I only stand up for short periods of time because of my back.	○	○
Because of my back, I try not to bend or kneel down.	○	○
I find it difficult to get out of a chair because of my back.	○	○
My back is painful almost all of the time.	○	○
I find it difficult to turn over in bed because of my back.	○	○
My appetite is not very good because of my back.	○	○
I have trouble putting on my socks (stockings) beause of the pain in my back.	○	○

Go To Next Question >

Skip To Next Section >>

DoD-ACT

SECTION
Roland Morris Disability Questionnaire

Remember, only choose YES if you are sure that the sentence describes you **today**.

	No	Yes
I only walk short distances because of my back pain.	○	○
I sleep less well because of my back pain.	○	○
Because of my back pain, I get dressed with help from someone else.	○	○
I sit down for most of the day because of my back.	○	○
I avoid heavy jobs around the house because of my back.	○	○
Because of my back pain, I am more irritable and bad tempered with people than usual.	○	○
Because of my back, I go upstairs more slowly than usual.	○	○
I stay in bed most of the time because of my back.	○	○

<< Previous   Next >>

DoD-ACT 1

Back Pain Function Scale

On the questions listed below, we are interested in knowing whether you are having **ANY DIFFICULTY** at all with the activities **because of your back problem** for which you are currently seeking attention. Please porvide an answer for each activity.

Today, do you or would you have any DIFFICULTY at all with the following activities BECAUSE OF YOUR BACK PROBLEM?

(choose one response on each line)

	Unable to perform activity	Extreme difficulty	Quite a bit of difficulty	Moderate difficulty	A little bit of difficulty	No difficulty
Any of your usual work, housework, or school activities	○	○	○	○	○	○
Your usual hobbies, recreational, or sporting activities	○	○	○	○	○	○
Performing heavy activities around your home	○	○	○	○	○	○
Bending or stooping	○	○	○	○	○	○
Putting on your shoes or socks (pantyhose)	○	○	○	○	○	○
Lifting a box of groceries from the floor	○	○	○	○	○	○

Next >>

DoD-ACT1

Back Pain Function Scale

Today, do you or would you have any DIFFICULTY at all with the following activities BECAUSE OF YOUR BACK PROBLEM?

(choose one response on each line)

	Unable to perform activity	Extreme difficulty	Quite a bit of difficulty	Moderate difficulty	A little bit of difficulty	No difficulty
Sleeping	○	○	○	○	○	○
Standing for 1 hour	○	○	○	○	○	○
Walking a mile	○	○	○	○	○	○
Going up or down 2 flights of stairs (about 20 stairs)	○	○	○	○	○	○
Sitting for 1 hour	○	○	○	○	○	○
Driving for 1 hour	○	○	○	○	○	○

<< Previous   Next >>

DoD-ACT 1

Bothersomeness

During the past week, how bothersome have each of the following symptoms been? (choose one response for each symptom)

	Not at all bothersome	Slightly bothersome	Moderately bothersome	Very bothersome	Extremely bothersome
Low back pain	○	○	○	○	○
Leg pain (sciatica)	○	○	○	○	○
Neck pain	○	○	○	○	○

During the past week, how much did pain interfere with your normal work (including both work outside the home and housework)?

- ○ Not at all
- ○ A little bit
- ○ Moderately
- ○ Quite a bit
- ○ Extremely

Next >>

DoD-ACT 1

Bothersomeness

If you had to spend the rest of your life with the symptoms you have right now, how would you feel about it?

- ◯ Very dissatisfied
- ◯ Somewhat dissatisfied
- ◯ Neither satisfied nor dissatisfied
- ◯ Somewhat satisfied
- ◯ Very satisfied

During the past 4 weeks, about how many days did you cut down on the things you usually do for more than half the day because of your low back pain?

[ ] # of days

During the past 4 weeks, how many days did low back pain keep you from going to work or school?

[ ] # of days

[<< Previous] [Next >>]

DoD-ACT1

Health Survey - PROMIS-29

In the past 7 days...

	Without any difficulty	With a little difficulty	With some difficulty	With much difficulty	Unable to do
Are you able to do chores such as vacuuming or yard work?	○	○	○	○	○
Are you able to go up and down stairs at a normal pace?	○	○	○	○	○
Are you able to go for a walk of at least 15 minutes?	○	○	○	○	○
Are you able to run errands and shop?	○	○	○	○	○

Next >>

DoD-ACT1

Health Survey - PROMIS-29

In the past 7 days...

	Never	Rarely	Sometimes	Often	Always
I felt fearful.	○	○	○	○	○
I found it hard to focus on anything other than my anxiety.	○	○	○	○	○
My worries overwhelmed me.	○	○	○	○	○
I felt uneasy.	○	○	○	○	○

Next >>

DoD-ACT1

Health Survey - PROMIS-29

In the past 7 days...

	Never	Rarely	Sometimes	Often	Always
I felt worthless.	○	○	○	○	○
I felt helpless.	○	○	○	○	○
I felt depressed.	○	○	○	○	○
I felt hopeless.	○	○	○	○	○

<< Previous   Next >>

DoD-ACT1

Health Survey - PROMIS-29

During the past 7 days...

	Not at all	A little bit	Somewhat	Quite a bit	Very much
I feeel fatigued.	○	○	○	○	○
I have trouble starting things because I am tired.	○	○	○	○	○

In the past 7 days...

	Not at all	A little bit	Somewhat	Quite a bit	Very much
How run-down did you feel on average?	○	○	○	○	○
How fatigued were you on average?	○	○	○	○	○

<< Previous   Next >>

DoD-ACT1

Health Survey - PROMIS-29

In the past 7 days...

	Very poor	Poor	Fair	Good	Very good
My sleep quality was...	○	○	○	○	○

In the past 7 days...

	Not at all	A little bit	Somewhat	Quite a bit	Very much
My sleep was refreshing.	○	○	○	○	○
I had a problem with my sleep.	○	○	○	○	○
I had difficulty falling asleep.	○	○	○	○	○

<< Previous   Next >>

DoD-ACT1

Health Survey - PROMIS-29

In the past 7 days...

	Not at all	A little bit	Somewhat	Quite a bit	Very much
I am satisfied with how much work I can do (include work at home).	○	○	○	○	○
I am satisfied with my ability to work (include work at home).	○	○	○	○	○
I am satisfied with my ability to do regular personal and household responsibilities.	○	○	○	○	○
I am satisfied with my ability to perform my daily routines.	○	○	○	○	○

<< Previous   Next >>

DoD-ACT 1

Health Survey - PROMIS-29

In the past 7 days...

	Not at all	A little bit	Somewhat	Quite a bit	Very much
How much did pain interfere with your day to day activities?	○	○	○	○	○
How much did pain interfere with work around the home?	○	○	○	○	○
How much did pain interfere with your ability to participate in social activities?	○	○	○	○	○
How much did pain interfere with your household chores?	○	○	○	○	○

<< Previous   Next >>

DoD-ACT1

Health Survey - PROMIS-29

In the past 7 days...

How would you rate your pain on average?

No Pain										Worst Imaginable Pain
◯ 0	◯ 1	◯ 2	◯ 3	◯ 4	◯ 5	◯ 6	◯ 7	◯ 8	◯ 9	◯ 10

Next >>

DoD-ACT1

Health Care and Medication Use

Since the beginning of your current episode of pain, from which of the following providers have you sought treatment for your low back pain?
(Choose all that apply)

- ☐ Primary care doctor
- ☐ Medical specialist (specify type below)
- ☐ Doctor of Osteopathy
- ☐ Doctor of Chiropractic
- ☐ Acupuncturist
- ☐ Massage therapist
- ☐ Physical therapist
- ☐ Pain clinic or pain specialist
- ☐ Counselor or mental health specialist
- ☐ N/A
- ☐ Other (specify type below)

Specify: ______________________

During the **past week**, how often have you taken **pain relieving medication** (including prescription and over-the-counter medications or supplements)?

- ○ 0 days
- ○ 1-2 days
- ○ 3-4 days
- ○ 5-6 days
- ○ 7 days

Next >>

DoD-ACT 1

Health Care and Medication Use

During the **past week**, have you taken **non-narcotic analgesics**?

Examples: Acetaminophen, Tylenol, Tylenol Extra Strength, Ultram, etc.

- ◯ No
- ◯ Yes, for back pain ONLY
- ◯ Yes, for other reasons ONLY
- ◯ Yes, for back pain AND other reasons

During the **past week**, have you taken **non-steroidal anti-inflammatory drugs (NSAIDS)**?

Examples: Advil, Aleve, Aspirin, Bextra, Celebrex, Disalcid, Excedrin, Excedrin PM, Feldene, Ibuprofen, Indomethacin, Meclomen, Motrin, Maproxen/Naprosyn, Relefen, Sulindac, Trilisate, Tolectic, etc.

- ◯ No
- ◯ Yes, for back pain ONLY
- ◯ Yes, for other reasons ONLY
- ◯ Yes, for back pain AND other reasons

<< Previous   Next >>

During the **past week**, have you taken **sedatives or muscles relaxants**?

Examples: Alprazolam, Ambien, Baclofen, Diazepam, Donnatal, Flexeril, Lorazepam, Meprobamate, Methocarbamol/Robaxin, Norflex, Phenergan, Phenobarbital, Skelaxin, Soma, Temazepam, tizanidine, Tranxene, Tylenol PM, Valium, Xanax, Zanaflex, etc.

- ◯ No
- ◯ Yes, for back pain ONLY
- ◯ Yes, for other reasons ONLY
- ◯ Yes, for back pain AND other reasons

During the **past week**, have you taken **narcotic analgesics**?

Examples: ASA w/ Codeine, Darvocet, Darvon, Demerol, Dilaudid, Fentanyl, Meperidine, Morphine, Oxycodone, Percodan, Talwin, Tylenol-3, Tylenol w/ Codeine, Tylox, Vicodin, etc.

- ◯ No
- ◯ Yes, for back pain ONLY
- ◯ Yes, for other reasons ONLY
- ◯ Yes, for back pain AND other reasons

<< Previous   Next >>

During the **past week**, have you taken **anti-depressants**?

Examples: Amitriptyline, Celexa, Desipramine, Doxepin, Effexor, Imipramine, Lexapro, Nortriptyline, Paxil, Prozac/Fluoxetine, Trazodone, Wellbutrin, Zoloft, etc.

- No
- Yes, for back pain ONLY
- Yes, for other reasons ONLY
- Yes, for back pain AND other reasons

During the **past week**, have you taken **supplements**?

Examples: Chondroitin Sulfate, fish oils, flax seeds/oils, Glucosamine, willow bark, etc.

- No
- Yes, for back pain ONLY
- Yes, for other reasons ONLY
- Yes, for back pain AND other reasons

<< Previous   Next >>

DoD-ACT1

Tobacco Use

Do you currently smoke cigarettes (smoked even 1 puff in the last 7 days)? (This includes name brand, generic, all natural and roll-your-own cigarettes) .

- ◯ No
- ◯ Yes

Do you currently use chewing tobacco or snuff (took even 1 dip in the last 7 days)?

- ◯ No
- ◯ Yes

Next >>

Tobacco Use

During the past 7 days, how many cigarettes did you smoke on a typical day?

(# of cigarettes)

How soon after you wake up do you usually smoke your first cigarette?

- Less than 5 minutes
- 5-30 minutes
- 31-60 minutes
- More than 60 minutes

How many years have you smoked?

(# of years)

<< Previous   Next >>

DoD-ACT1

Tobacco Use

Do you experience strong cravings to smoke when you have to go without a cigarette for more than an hour or two?

- ◯ Never
- ◯ Seldom
- ◯ Sometimes
- ◯ Often
- ◯ Always

Have you been advised to quit smoking by your: (check all that apply)

- ☐ Doctor of Chiropractic
- ☐ Medical Doctor
- ☐ Dentist
- ☐ Nurse/Physician Assistant
- ☐ Dental Hygientist
- ☐ Other Health Professional
- ☐ None

In the past year, how many times have you made a serious attempt to quit smoking (A serious attempt is 24 hours or more without smoking)?

- ◯ None
- ◯ 1 time
- ◯ 2 times
- ◯ 3 times
- ◯ More than 3 times

<< Previous   Next >>

Tobacco Use

If you made a serious attempt to quit, which of the following methods did you use? (check all that apply)

- [ ] Went cold turkey (quit with no help)
- [ ] Made a quit plan
- [ ] Set a specific date to quit
- [ ] Nicotine patches, nicotine gum or nicotine lozenges
- [ ] Prescription medication (Zyban, Wellbutrin, Chantix)
- [ ] Telephone tobacco quit line
- [ ] Self-help guide
- [ ] Chiropractic adjustment/care
- [ ] Acupuncture/acupressure
- [ ] Massage
- [ ] Hypnosis
- [ ] Biofeedback
- [ ] Meditation/yoga/relaxation/imagery
- [ ] Herbal or botanical supplements
- [ ] Homeopathy
- [ ] Other

If Other, please specify: ____________________

<< Previous   Next >>

Are you seriously thinking about quiting smoking in the next 30 days?

- No
- Yes

Mark the number that shows how you feel about quitting.

- 0 No thought of quitting
- 1
- 2 Should consider quitting some day
- 3
- 4 Should quit but not quite ready
- 5
- 6 Thinking about cutting down or quitting
- 7
- 8 Have cut down and seriously considering quitting
- 9
- 10 Ready to quit now

<< Previous   Next >>

DoD-ACT 1

Tobacco Use

Do you currently use the following brands of chewing tobacco/snuff?

Loose moist snuff (Copenhagen, Cougar, Kodiak, Skoal, etc.)	◯No	◯Yes
Snuff pouches (Skoal bandits, etc.)	◯No	◯Yes
Snus (Camel, etc.)	◯No	◯Yes
Chew (Beech-Nut, Red Man, Granger, etc.)	◯No	◯Yes

In a typical week, how many days do you use chewing tobacco or snuff?

(# of days / week)

How many days does a can/tin/pouch last you?

(# of days)

<< Previous   Next >>

DoD-ACT

SECTION
Tobacco Use

How soon after you wake up do you use chewing tobacco or snuff?

- Less than 5 minutes
- 5-30 minutes
- 31-60 minutes
- More than 60 minutes

Do you swallow the tobacco juice from chewing tobacco?

- Never
- Sometimes
- Frequently

How many years have you chewed tobacco or dipped snuff?

(# of years)

<< Previous Next >>

DoD-ACT 1

Tobacco Use

Do you experience strong cravings for a dip/chew when you go more than one hour or two without one?

- ◯ Never
- ◯ Seldom
- ◯ Sometimes
- ◯ Often
- ◯ Always

Have you been advised to quit chewing tobacco by your: (check all that apply)

- ☐ Doctor of Chiropractic
- ☐ Medical Doctor
- ☐ Dentist
- ☐ Nurse/Physician Assistant
- ☐ Dental Hygientist
- ☐ Other Health Professional
- ☐ None

In the past year, how many times have you made a serious attempt to quit chewing tobacco or snuff? (A serious quit attempt is 24 hours or more without chewing).

- ◯ None
- ◯ 1 time
- ◯ 2 times
- ◯ 3 times
- ◯ More than 3 times

<< Previous   Next >>

Tobacco Use

If you made a serious attempt to quit, which of the following methods did you use? (check all that apply)

- ☐ Went cold turkey (quit with no help)
- ☐ Made a quit plan
- ☐ Set a specific date to quit
- ☐ Nicotine patches, nicotine gum or nicotine lozenges
- ☐ Prescription medication (Zyban, Wellbutrin, Chantix)
- ☐ Telephone tobacco quit line
- ☐ Self-help guide
- ☐ Chiropractic adjustment/care
- ☐ Acupuncture/acupressure
- ☐ Massage
- ☐ Hypnosis
- ☐ Biofeedback
- ☐ Meditation/yoga/relaxation/imagery
- ☐ Herbal or botanical supplements
- ☐ Homeopathy
- ☐ Other

If Other, please specify: ____________________

<< Previous   Next >>

DoD-ACT1

Tobacco Use

Are you seriously thinking about quiting chewing tobacco/snuff in the next 30 days?

- No
- Yes

Mark the number that shows how you feel about quitting.

- 0 No thought of quitting
- 1
- 2 Should consider quitting some day
- 3
- 4 Should quit but not quite ready
- 5
- 6 Thinking about cutting down or quitting
- 7
- 8 Have cut down and seriously considering quitting
- 9
- 10 Ready to quit now

<< Previous   Next >>

DoD-ACT

SECTION
Expectations

How helpful do you believe medical care **plus** chiropractic care will be for your current low back pain?

Not helpful at all — Extremely helpful

0 1 2 3 4 5 6 7 8 9 10

How helpful do you believe medical care alone will be for your current low back pain?

Not helpful at all — Extremely helpful

0 1 2 3 4 5 6 7 8 9 10

One month from now, do you expect your low back pain to be:

Completely gone
Much better
Moderately better
A little better
About the same
A little worse
Much worse

DoD-ACT1

Episodes of Pain

An **episode of pain** is defined as at least some low back pain requiring you to seek treatment or modify your activity during consecutive weeks (a week or more with no pain separates episodes).

Have you had **more than one episode** of low back pain?

- ◯ No
- ◯ Yes

If Yes,

Date of first episode of low back pain:

Month... / ______ (month / year)

How many episodes have you had?

______ (# of episodes)

Is the rate of frequency of your low back pain episodes:

- ◯ Increasing in frequency
- ◯ Decreasing in frequency
- ◯ Unchanged in frequency

Next >>

DoD-ACT

SECTION
Demographic Information

What is your marital status?

- Married or living with significant other
- Divorced or separated
- Widowed
- Never been married

What is the highest grade or level of school you have completed or the highest degree you have received?

- Some grade school or high school
- High school graduate
- GED or equivalent
- Some college or other program, no degree
- Associate degree: occupational, technical, or vocational
- Associate degree: academic program
- Bachelor's degree (for example: BA, AB, BS, BBA)
- Master's degree (for example: MA, MS, MEng, MEd, MBA)
- Professional school degree (for example: MD, DC, DDS, DO)
- Doctoral degree (for example: PhD, EdD)

Which armed forces are you a member of?

- Navy
- Army
- Marines
- Air Force
- Coast Guard
- Air National Guard
- Army National Guard
- Special Operations
- ROTC
- USPHS
- NOAA

Next >>

DoD-ACT 1

Demographic Information

Have you ever been to a Doctor of Chiropractic?

- ◯ No
- ◯ Yes

If yes, do you consider yourself someone who receives regular chiropractic care?

- ◯ No
- ◯ Yes

If yes, how often would you say you receive chiropractic care?

- ◯ Annually
- ◯ Semi-annually
- ◯ Monthly
- ◯ Bi-weekly
- ◯ Weekly

<< Previous   Next >>

DoD-ACT

SECTION
Demographic Information

What is your household income?

- Less than $20,000
- $20,000 - $39,999
- $40,000 - $59,000
- $60,000 - $79,000
- $80,000 or more

How would you describe the amount of physical activity in your daily routine?

- No physical activity
- Very light physical activity
- Light physical activity
- Moderate physical activity
- Heavy physical activity
- Very heavy physical activity

What is your height?

(feet)
(inches: up to 1 decimal place)

What is your weight?

(lbs)

<< Previous Next >>

Week 2 and Week 4 Assessments

DoD-ACT1

SECTION
-
Pain Rating Scales

Select the number that best describes your low back pain at its **worst** during the **past 24 hours**?

No Pain — Worst Possible Pain

○0 ○1 ○2 ○3 ○4 ○5 ○6 ○7 ○8 ○9 ○10

Select the number that best describes your **average** low back pain during the **past week**?

No Pain — Worst Possible Pain

○0 ○1 ○2 ○3 ○4 ○5 ○6 ○7 ○8 ○9 ○10

Next >>

8/30/2011 12:03:38 PM

DoD-ACT1

Roland Morris Disability Questionnaire

When your back hurts, you may find it difficult to do some of the things you normally do.

This list contains sentences that people have used to describe themselves when they have back pain. When you read them, you may find that some stand out because they describe you **today**. As you read the list, thing of yourself **today**. When you read a sentence that describes how you feel today, choose YES. If the sentence does not describe you, then choose NO.

Remember, only choose YES if you are sure that the sentence describes you **today**.

	No	Yes
I stay home most of the time because of my back.	○	○
I change position frequently to try and get my back comfortable.	○	○
I walk more slowly than usual because of my back.	○	○
Because of my back, I am not doing any jobs that I usually do around the house.	○	○
Because of my back, I use a handrail to get upstairs.	○	○
Because of my back, I lie down to rest more often.	○	○
Because of my back, I have to hold on to something to get out of an easy chair.	○	○
Because of my back, I try to get other people to do things for me.	○	○

Next >>

8/30/2011 12:05:35 PM

DoD-ACT1

Roland Morris Disability Questionnaire

Remember, only choose YES if you are sure that the sentence describes you **today**.

	No	Yes
I get dressed more slowly than usual because of my back.	◯	◯
I only stand up for short periods of time because of my back.	◯	◯
Because of my back, I try not to bend or kneel down.	◯	◯
I find it difficult to get out of a chair because of my back.	◯	◯
My back is painful almost all of the time.	◯	◯
I find it difficult to turn over in bed because of my back.	◯	◯
My appetite is not very good because of my back.	◯	◯
I have trouble putting on my socks (stockings) beause of the pain in my back.	◯	◯

Go To Next Question >

Skip To Next Section >>

DoD-ACT

SECTION
Roland Morris Disability Questionnaire

Remember, only choose YES if you are sure that the sentence describes you **today**.

	No	Yes
I only walk short distances because of my back pain.		
I sleep less well because of my back pain.		
Because of my back pain, I get dressed with help from someone else.		
I sit down for most of the day because of my back.		
I avoid heavy jobs around the house because of my back.		
Because of my back pain, I am more irritable and bad tempered with people than usual.		
Because of my back, I go upstairs more slowly than usual.		
I stay in bed most of the time because of my back.		

<< Previous   Next >>

DoD-ACT1

Back Pain Function Scale

On the questions listed below, we are interested in knowing whether you are having **ANY DIFFICULTY** at all with the activities **because of your back problem** for which you are currently seeking attention. Please porvide an answer for each activity.

Today, do you or would you have any DIFFICULTY at all with the following activities BECAUSE OF YOUR BACK PROBLEM?

(choose one response on each line)

	Unable to perform activity	Extreme difficulty	Quite a bit of difficulty	Moderate difficulty	A little bit of difficulty	No difficulty
Any of your usual work, housework, or school activities	○	○	○	○	○	○
Your usual hobbies, recreational, or sporting activities	○	○	○	○	○	○
Performing heavy activities around your home	○	○	○	○	○	○
Bending or stooping	○	○	○	○	○	○
Putting on your shoes or socks (pantyhose)	○	○	○	○	○	○
Lifting a box of groceries from the floor	○	○	○	○	○	○

Next >>

DoD-ACT1

Back Pain Function Scale

Today, do you or would you have any DIFFICULTY at all with the following activities BECAUSE OF YOUR BACK PROBLEM?

(choose one response on each line)

	Unable to perform activity	Extreme difficulty	Quite a bit of difficulty	Moderate difficulty	A little bit of difficulty	No difficulty
Sleeping	○	○	○	○	○	○
Standing for 1 hour	○	○	○	○	○	○
Walking a mile	○	○	○	○	○	○
Going up or down 2 flights of stairs (about 20 stairs)	○	○	○	○	○	○
Sitting for 1 hour	○	○	○	○	○	○
Driving for 1 hour	○	○	○	○	○	○

<< Previous   Next >>

DoD-ACT1

Bothersomeness

During the past week, how bothersome have each of the following symptoms been? (choose one response for each symptom)

	Not at all bothersome	Slightly bothersome	Moderately bothersome	Very bothersome	Extremely bothersome
Low back pain	○	○	○	○	○
Leg pain (sciatica)	○	○	○	○	○
Neck pain	○	○	○	○	○

During the past week, how much did pain interfere with your normal work (including both work outside the home and housework)?

- ○ Not at all
- ○ A little bit
- ○ Moderately
- ○ Quite a bit
- ○ Extremely

Next >>

DoD-ACT1

Bothersomeness

If you had to spend the rest of your life with the symptoms you have right now, how would you feel about it?

- ◯ Very dissatisfied
- ◯ Somewhat dissatisfied
- ◯ Neither satisfied nor dissatisfied
- ◯ Somewhat satisfied
- ◯ Very satisfied

During the past 4 weeks, about how many days did you cut down on the things you usually do for more than half the day because of your low back pain?

______ # of days

During the past 4 weeks, how many days did low back pain keep you from going to work or school?

______ # of days

<< Previous   Next >>

8/30/2011 12:10:21 PM

DoD-ACT1

Health Care and Medication Use

Since the beginning of your current episode of pain, from which of the following providers have you sought treatment for your low back pain?
(Choose all that apply)

- ☐ Primary care doctor
- ☐ Medical specialist (specify type below)
- ☐ Doctor of Osteopathy
- ☐ Doctor of Chiropractic
- ☐ Acupuncturist
- ☐ Massage therapist
- ☐ Physical therapist
- ☐ Pain clinic or pain specialist
- ☐ Counselor or mental health specialist
- ☐ N/A
- ☐ Other (specify type below)

Specify:

During the **past week**, how often have you taken **pain relieving medication** (including prescription and over-the-counter medications or supplements)?

- ○ 0 days
- ○ 1-2 days
- ○ 3-4 days
- ○ 5-6 days
- ○ 7 days

Next >>

8/30/2011 12:12:25 PM

DoD-ACT1

Health Care and Medication Use

During the **past week**, have you taken **non-narcotic analgesics**?

Examples: Acetaminophen, Tylenol, Tylenol Extra Strength, Ultram, etc.

- ◯ No
- ◯ Yes, for back pain ONLY
- ◯ Yes, for other reasons ONLY
- ◯ Yes, for back pain AND other reasons

During the **past week**, have you taken **non-steroidal anti-inflammatory drugs (NSAIDS)**?

Examples: Advil, Aleve, Aspirin, Bextra, Celebrex, Disalcid, Excedrin, Excedrin PM, Feldene, Ibuprofen, Indomethacin, Meclomen, Motrin, Maproxen/Naprosyn, Relefen, Sulindac, Trilisate, Tolectic, etc.

- ◯ No
- ◯ Yes, for back pain ONLY
- ◯ Yes, for other reasons ONLY
- ◯ Yes, for back pain AND other reasons

<< Previous | Next >>

DoD-ACT1

Health Care and Medication Use

During the **past week**, have you taken **sedatives or muscles relaxants**?

Examples: Alprazolam, Ambien, Baclofen, Diazepam, Donnatal, Flexeril, Lorazepam, Meprobamate, Methocarbamol/Robaxin, Norflex, Phenergan, Phenobarbital, Skelaxin, Soma, Temazepam, tizanidine, Tranxene, Tylenol PM, Valium, Xanax, Zanaflex, etc.

- ◯ No
- ◯ Yes, for back pain ONLY
- ◯ Yes, for other reasons ONLY
- ◯ Yes, for back pain AND other reasons

During the **past week**, have you taken **narcotic analgesics**?

Examples: ASA w/ Codeine, Darvocet, Darvon, Demerol, Dilaudid, Fentanyl, Meperidine, Morphine, Oxycodone, Percodan, Talwin, Tylenol-3, Tylenol w/ Codeine, Tylox, Vicodin, etc.

- ◯ No
- ◯ Yes, for back pain ONLY
- ◯ Yes, for other reasons ONLY
- ◯ Yes, for back pain AND other reasons

<< Previous   Next >>

DoD-ACT1

Health Care and Medication Use

During the **past week**, have you taken **anti-depressants**?

Examples: Amitriptyline, Celexa, Desipramine, Doxepin, Effexor, Imipramine, Lexapro, Nortriptyline, Paxil, Prozac/Fluoxetine, Trazodone, Wellbutrin, Zoloft, etc.

- ◯ No
- ◯ Yes, for back pain ONLY
- ◯ Yes, for other reasons ONLY
- ◯ Yes, for back pain AND other reasons

During the **past week**, have you taken **supplements**?

Examples: Chondroitin Sulfate, fish oils, flax seeds/oils, Glucosamine, willow bark, etc.

- ◯ No
- ◯ Yes, for back pain ONLY
- ◯ Yes, for other reasons ONLY
- ◯ Yes, for back pain AND other reasons

<< Previous   Next >>

8/30/2011   12:13:02 PM

DoD-ACT1

Satisfaction with Care

How satisfied are you with your care?

Not at all satisfied ... **Extremely satisfied**

○0 ○1 ○2 ○3 ○4 ○5 ○6 ○7 ○8 ○9 ○10

Compared to your first visit, your low back pain is:

- ○Completely gone
- ○Much better
- ○Moderately better
- ○A little better
- ○About the same
- ○A little worse
- ○Much worse

Next >>

8/30/2011 12:40:23 PM

DoD-ACT1

Self-Reported Care

During the past 2 weeks, what type of care have you had?

- ☐ Medical care
- ☐ Rehabilitation / Physical therapy
- ☐ Chiropractic care
- ☐ None of the above

Next >>

8/30/2011 12:40:51 PM

DoD-ACT1

Self-Reported Care

Medical care

What type of doctor did you see:

- ☐ Primary care MD
- ☐ Orthopaedist
- ☐ Neurologist
- ☐ Other (specify below)

Specify:

How many visits did you have (total)?

visits

Did your doctor prescribe medication?

- ○ No
- ○ Yes

Indicate any self-care instructions given by the doctor(s)? (mark all that apply)

- ☐ Exercise
- ☐ Stretching
- ☐ Over-the-counter medication
- ☐ Restrictions on daily living
- ☐ Bed rest
- ☐ Other

<< Previous   Next >>

8/30/2011   12:41:02 PM

DoD-ACT1

Self-Reported Care

Chiropractic care

How many visits did you have (total)?

visits

Indicate the type of care that you received? (mark all that apply)

- ☐ Manipulation/adjustment
- ☐ Self-care instructions
- ☐ Ice
- ☐ Heat
- ☐ Electrical stimulation
- ☐ Ultrasound
- ☐ Soft tissue/massage
- ☐ Tape/bracing
- ☐ Other

<< Previous   Next >>

8/30/2011   12:41:14 PM

DoD-ACT1

Self-Reported Care

Self-care

Indicate care that you initiated on your own without the recommendation of a healthcare provider? (mark all that apply)

- ☐ Exercise
- ☐ Over-the-counter medication
- ☐ Nutritional supplements
- ☐ Diet
- ☐ Massage
- ☐ Acupuncture
- ☐ None of the above
- ☐ Other (specify below)

Specify:

<< Previous   Next >>

8/30/2011   12:41:24 PM

DoD-ACT

SECTION
Reactions and Discomforts

During the past 2 weeks, have you seen a healthcare provider for any reason besides routine care*?

- ○ No
- ○ Yes

*Any planned visit to the physician such as preventive care, routine physical exams, or maintenance exams. Please be sure to tell us of any unplanned visit to the doctor or hospital.

If yes, please describe the reason for the visit and any treatment that was provided.

Next >>

DoD-ACT

SECTION
Reactions and Discomforts

Were you hospitalized during the course of treatment?

○ No
◉ Yes

If yes, please describe*:

*Please be sure to include whether it was an ER visit only or not (and length of visit), hospital admission and discharge dates.

<< Previous Next >>

DoD-ACT

SECTION
Reactions and Discomforts

During the past 2 weeks, have you experienced any discomfort and/or an unpleasant reaction that you think could be connected to the treatment you received in this study?

- ○ No
- ○ Yes

If yes, indicate what discomforts or reactions you experienced.
(check all that apply)

- ☐ Reaction to medication(s)
- ☐ Muscle and/or joint soreness
- ☐ Neck pain
- ☐ Headache
- ☐ Pain or tingling down the arm/hand or leg/foot
- ☐ Mid and upper back
- ☐ Dizziness
- ☐ Broken rib
- ☐ Broken hip
- ☐ Other

[<< Previous] [Next >>]

DoD-ACT

SECTION
Reactions and Discomforts

Reaction to medication(s)

Please describe the study treatment that you believe is connected to this discomfort/reaction:

Were you hospitalized because of this reaction/discomfort?

- ○ No
- ○ Yes

If Yes, please describe*:

*Please be sure to include admission and discharge dates.

<< Previous Next >>

DoD-ACT

SECTION
Reactions and Discomforts

Reaction to medication(s), continued

Name of medication:

Describe your reaction to the medicine:

How would you rate the amount of discomfort?

No Discomfort … **Unbearable Discomfort**

○0 ○1 ○2 ○3 ○4 ○5 ○6 ○7 ○8 ○9 ○10

<< Previous   Next >>

DoD-ACT

SECTION
Reactions and Discomforts

Reaction to medication(s), continued

How long after the treatment did the discomfort/reaction begin?

- ◯ Less than 30 minutes
- ◯ 30 minutes to 4 hours
- ◯ 4 hours to 24 hours
- ◯ More than 24 hours

How long did the discomfort last?

- ◯ Less than 1 day
- ◯ 1 day - 1 week
- ◯ More than 1 week
- ◯ Ongoing

Did you have to modify your normal daily activities at home and/or work?

- ◯ Not at all
- ◯ A little
- ◯ Moderately
- ◯ Could not perform daily activities

If you could not perform daily activities, please explain:

<< Previous   Next >>

DoD-ACT

SECTION
Reactions and Discomforts

Muscle and/or joint soreness

Please describe the study treatment that you believe is connected to this discomfort/reaction:

Were you hospitalized because of this reaction/discomfort?

- ◯ No
- ◯ Yes

If Yes, please describe*:

*Description should include date of admission and date of discharge.

<< Previous Next >>

DoD-ACT

SECTION
Reactions and Discomforts

Muscle and/or joint soreness, continued

Describe the discomfort from your muscle and/or joint soreness:

How would you rate the amount of discomfort?

No Discomfort **Unbearable Discomfort**

○0 ○1 ○2 ○3 ○4 ○5 ○6 ○7 ○8 ○9 ○10

<< Previous Next >>

SECTION
Reactions and Discomforts

Muscle and/or joint soreness, continued

How long after the treatment did the discomfort begin?

- ○ Less than 30 minutes
- ○ 30 minutes to 4 hours
- ○ 4 hours to 24 hours
- ○ More than 24 hours

How long did the discomfort last?

- ○ Less than 1 day
- ○ 1 day - 1 week
- ○ More than 1 week
- ○ Ongoing

Did you have to modify your normal daily activities at home and/or work?

- ○ Not at all
- ○ A little
- ○ Moderately
- ○ Could not perform daily activities

If you could not perform daily activities, please explain:

<< Previous   Next >>

DoD-ACT

SECTION
Reactions and Discomforts

Neck Pain

Please describe the study treatment that you believe is connected to this discomfort/reaction:

Were you hospitalized because of this reaction/discomfort?

- ○ No
- ○ Yes

If Yes, please describe*:

*Description should include date of admission and date of discharge.

<< Previous   Next >>

DoD-ACT

SECTION
Reactions and Discomforts

Neck pain, continued

Describe the discomfort from your neck pain:

How would you rate the amount of discomfort?

No Discomfort … **Unbearable Discomfort**

○0 ○1 ○2 ○3 ○4 ○5 ○6 ○7 ○8 ○9 ○10

<< Previous   Next >>

DoD-ACT

SECTION
Reactions and Discomforts

Neck pain, continued

How long after the treatment did the discomfort begin?

- ◯ Less than 30 minutes
- ◯ 30 minutes to 4 hours
- ◯ 4 hours to 24 hours
- ◯ More than 24 hours

How long did the discomfort last?

- ◯ Less than 1 day
- ◯ 1 day - 1 week
- ◯ More than 1 week
- ◯ Ongoing

Did you have to modify your normal daily activities at home and/or work?

- ◯ Not at all
- ◯ A little
- ◯ Moderately
- ◯ Could not perform daily activities

If you could not perform daily activities, please explain:

<< Previous   Next >>

DoD-ACT

SECTION
Reactions and Discomforts

Headache

Please describe the study treatment that you believe is connected to this discomfort/reaction:

Were you hospitalized because of this reaction/discomfort?

- ○ No
- ○ Yes

If Yes, please describe*:

*Description should include date of admission and date of discharge.

<< Previous   Next >>

DoD-ACT

SECTION
Reactions and Discomforts

Headache, continued

Describe the discomfort from your headache:

How would you rate the amount of discomfort?

No Discomfort **Unbearable Discomfort**

○ 0 ○ 1 ○ 2 ○ 3 ○ 4 ○ 5 ○ 6 ○ 7 ○ 8 ○ 9 ○ 10

<< Previous Next >>

DoD-ACT

SECTION
Reactions and Discomforts

Headache, continued

How long after the treatment did the discomfort begin?

- ○ Less than 30 minutes
- ○ 30 minutes to 4 hours
- ○ 4 hours to 24 hours
- ○ More than 24 hours

How long did the discomfort last?

- ○ Less than 1 day
- ○ 1 day - 1 week
- ○ More than 1 week
- ○ Ongoing

Did you have to modify your normal daily activities at home and/or work?

- ○ Not at all
- ○ A little
- ○ Moderately
- ○ Could not perform daily activities

If you could not perform daily activities, please explain:

<< Previous   Next >>

DoD-ACT

SECTION
Reactions and Discomforts

Pain or tingling down the arm/hand or leg/foot

Please describe the study treatment that you believe is connected to this discomfort/reaction:

Were you hospitalized because of this reaction/discomfort?

- ○ No
- ○ Yes

If Yes, please describe*:

*Description should include date of admission and date of discharge.

<< Previous   Next >>

DoD-ACT

SECTION
Reactions and Discomforts

Pain or tingling down the arm/hand or leg/foot, continued

Describe the discomfort:

How would you rate the amount of discomfort?

No Discomfort **Unbearable Discomfort**

○0 ○1 ○2 ○3 ○4 ○5 ○6 ○7 ○8 ○9 ○10

<< Previous Next >>

DoD-ACT

SECTION
Reactions and Discomforts

Pain or tingling down the arm/hand or leg/foot, continued

How long after the treatment did the discomfort begin?

- ○ Less than 30 minutes
- ○ 30 minutes to 4 hours
- ○ 4 hours to 24 hours
- ○ More than 24 hours

How long did the discomfort last?

- ○ Less than 1 day
- ○ 1 day - 1 week
- ○ More than 1 week
- ○ Ongoing

Did you have to modify your normal daily activities at home and/or work?

- ○ Not at all
- ○ A little
- ○ Moderately
- ○ Could not perform daily activities

If you could not perform daily activities, please explain:

<< Previous   Next >>

DoD-ACT

SECTION
Reactions and Discomforts

Mid and upper back

Please describe the study treatment that you believe is connected to this discomfort/reaction:

Were you hospitalized because of this reaction/discomfort?

- ◯ No
- ◯ Yes

If Yes, please describe*:

*Description should include date of admission and date of discharge.

<< Previous   Next >>

DoD-ACT

SECTION
Reactions and Discomforts

Mid and upper back, continued

Describe the discomfort:

How would you rate the amount of discomfort?

No Discomfort **Unbearable Discomfort**

○0 ○1 ○2 ○3 ○4 ○5 ○6 ○7 ○8 ○9 ○10

<< Previous Next >>

DoD-ACT

SECTION
Reactions and Discomforts

Mid and upper back, continued

How long after the treatment did the discomfort begin?

- ○ Less than 30 minutes
- ○ 30 minutes to 4 hours
- ○ 4 hours to 24 hours
- ○ More than 24 hours

How long did the discomfort last?

- ○ Less than 1 day
- ○ 1 day - 1 week
- ○ More than 1 week
- ○ Ongoing

Did you have to modify your normal daily activities at home and/or work?

- ○ Not at all
- ○ A little
- ○ Moderately
- ○ Could not perform daily activities

If you could not perform daily activities, please explain:

<< Previous   Next >>

DoD-ACT

SECTION
Reactions and Discomforts

Dizziness

Please describe the study treatment that you believe is connected to this discomfort/reaction:

Were you hospitalized because of this reaction/discomfort?

○ No
○ Yes

If Yes, please describe*:

*Description should include date of admission and date of discharge.

<< Previous   Next >>

DoD-ACT

SECTION
Reactions and Discomforts

Dizziness, continued

Describe the discomfort:

How would you rate the amount of discomfort?

No Discomfort **Unbearable Discomfort**

○0 ○1 ○2 ○3 ○4 ○5 ○6 ○7 ○8 ○9 ○10

<< Previous Next >>

DoD-ACT

SECTION
Reactions and Discomforts

Dizziness, continued

How long after the treatment did the discomfort begin?

- ○ Less than 30 minutes
- ○ 30 minutes to 4 hours
- ○ 4 hours to 24 hours
- ○ More than 24 hours

How long did the discomfort last?

- ○ Less than 1 day
- ○ 1 day - 1 week
- ○ More than 1 week
- ○ Ongoing

Did you have to modify your normal daily activities at home and/or work?

- ○ Not at all
- ○ A little
- ○ Moderately
- ○ Could not perform daily activities

If you could not perform daily activities, please explain:

<< Previous Next >>

DoD-ACT

SECTION
Reactions and Discomforts

Broken rib

Please describe the study treatment that you believe is connected to this discomfort/reaction:

Were you hospitalized because of this reaction/discomfort?

- ○ No
- ○ Yes

If Yes, please describe*:

*Description should include date of admission and date of discharge.

<< Previous   Next >>

DoD-ACT

SECTION
Reactions and Discomforts

Broken rib, continued

Describe the fracture:

How long after the treatment did you first suspect a fracture/injury?

- ○ Less than 30 minutes
- ○ 30 minutes to 4 hours
- ○ 4 hours to 24 hours
- ○ More than 24 hours

How long was it until you sought medical treatment for the fracture?

- ○ Less than 1 day
- ○ 1 day - 1 week
- ○ More than 1 week
- ○ Ongoing

<< Previous   Next >>

DoD-ACT

SECTION
Reactions and Discomforts

Broken hip

Please describe the study treatment that you believe is connected to this discomfort/reaction:

Were you hospitalized because of this reaction/discomfort?

- ○ No
- ○ Yes

If Yes, please describe*:

*Description should include date of admission and date of discharge.

<< Previous Next >>

DoD-ACT

SECTION
Reactions and Discomforts

Broken hip, continued

Describe the fracture:

How long after the treatment did you first suspect a fracture/injury?

- ○ Less than 30 minutes
- ○ 30 minutes to 4 hours
- ○ 4 hours to 24 hours
- ○ More than 24 hours

How long was it until you sought medical treatment for the fracture?

- ○ Less than 1 day
- ○ 1 day - 1 week
- ○ More than 1 week
- ○ Ongoing

<< Previous   Next >>

DoD-ACT

SECTION
Reactions and Discomforts

Other reaction/discomfort

Please describe the study treatment that you believe is connected to this discomfort/reaction:

Were you hospitalized because of this reaction/discomfort?

- ○ No
- ○ Yes

If Yes, please describe*:

*Description should include date of admission and date of discharge.

<< Previous   Next >>

DoD-ACT

SECTION
Reactions and Discomforts

Other reaction/discomfort, continued

Describe the reaction/discomfort:

How would you rate the amount of discomfort?

No Discomfort **Unbearable Discomfort**

○0 ○1 ○2 ○3 ○4 ○5 ○6 ○7 ○8 ○9 ○10

<< Previous Next >>

DoD-ACT

SECTION
Reactions and Discomforts

Other reaction/discomfort, continued

How long after the treatment did the reaction/discomfort begin?

- ○ Less than 30 minutes
- ○ 30 minutes to 4 hours
- ○ 4 hours to 24 hours
- ○ More than 24 hours

How long did the reaction/discomfort last?

- ○ Less than 1 day
- ○ 1 day - 1 week
- ○ More than 1 week
- ○ Ongoing

How much did the reaction/discomfort affect your normal daily activities at home and/or work?

- ○ Not at all
- ○ A little
- ○ Moderately
- ○ Could not perform daily activities

If you could not perform daily activities, please explain:

<< Previous   Next >>

DoD-ACT

Thank you!

Thank you for your time!

Your next questionnaire will be available on . You will receive a reminder at that time.

Please take this time to verify your contact information is current.

DoD-ACT1

SECTION
-
Pain Rating Scales

Select the number that best describes your low back pain at its **worst** during the **past 24 hours**?

No Pain ... **Worst Possible Pain**

◯0 ◯1 ◯2 ◯3 ◯4 ◯5 ◯6 ◯7 ◯8 ◯9 ◯10

Select the number that best describes your **average** low back pain during the **past week**?

No Pain ... **Worst Possible Pain**

◯0 ◯1 ◯2 ◯3 ◯4 ◯5 ◯6 ◯7 ◯8 ◯9 ◯10

Next >>

DoD-ACT1

Roland Morris Disability Questionnaire

When your back hurts, you may find it difficult to do some of the things you normally do.

This list contains sentences that people have used to describe themselves when they have back pain. When you read them, you may find that some stand out because they describe you **today**. As you read the list, thing of yourself **today**. When you read a sentence that describes how you feel today, choose YES. If the sentence does not describe you, then choose NO.

Remember, only choose YES if you are sure that the sentence describes you **today**.

	No	Yes
I stay home most of the time because of my back.	○	○
I change position frequently to try and get my back comfortable.	○	○
I walk more slowly than usual because of my back.	○	○
Because of my back, I am not doing any jobs that I usually do around the house.	○	○
Because of my back, I use a handrail to get upstairs.	○	○
Because of my back, I lie down to rest more often.	○	○
Because of my back, I have to hold on to something to get out of an easy chair.	○	○
Because of my back, I try to get other people to do things for me.	○	○

Next >>

DoD-ACT 1

Roland Morris Disability Questionnaire

Remember, only choose YES if you are sure that the sentence describes you **today**.

	No	Yes
I get dressed more slowly than usual because of my back.	○	○
I only stand up for short periods of time because of my back.	○	○
Because of my back, I try not to bend or kneel down.	○	○
I find it difficult to get out of a chair because of my back.	○	○
My back is painful almost all of the time.	○	○
I find it difficult to turn over in bed because of my back.	○	○
My appetite is not very good because of my back.	○	○
I have trouble putting on my socks (stockings) beause of the pain in my back.	○	○

Go To Next Question >

Skip To Next Section >>

DoD-ACT

SECTION
Roland Morris Disability Questionnaire

Remember, only choose YES if you are sure that the sentence describes you **today**.

	No	Yes
I only walk short distances because of my back pain.		
I sleep less well because of my back pain.		
Because of my back pain, I get dressed with help from someone else.		
I sit down for most of the day because of my back.		
I avoid heavy jobs around the house because of my back.		
Because of my back pain, I am more irritable and bad tempered with people than usual.		
Because of my back, I go upstairs more slowly than usual.		
I stay in bed most of the time because of my back.		

<< Previous   Next >>

DoD-ACT1

Back Pain Function Scale

On the questions listed below, we are interested in knowing whether you are having **ANY DIFFICULTY** at all with the activities **because of your back problem** for which you are currently seeking attention. Please porvide an answer for each activity.

Today, do you or would you have any DIFFICULTY at all with the following activities BECAUSE OF YOUR BACK PROBLEM?

(choose one response on each line)

	Unable to perform activity	Extreme difficulty	Quite a bit of difficulty	Moderate difficulty	A little bit of difficulty	No difficulty
Any of your usual work, housework, or school activities	○	○	○	○	○	○
Your usual hobbies, recreational, or sporting activities	○	○	○	○	○	○
Performing heavy activities around your home	○	○	○	○	○	○
Bending or stooping	○	○	○	○	○	○
Putting on your shoes or socks (pantyhose)	○	○	○	○	○	○
Lifting a box of groceries from the floor	○	○	○	○	○	○

Next >>

DoD-ACT 1

Back Pain Function Scale

Today, do you or would you have any DIFFICULTY at all with the following activities BECAUSE OF YOUR BACK PROBLEM?

(choose one response on each line)

	Unable to perform activity	Extreme difficulty	Quite a bit of difficulty	Moderate difficulty	A little bit of difficulty	No difficulty
Sleeping	○	○	○	○	○	○
Standing for 1 hour	○	○	○	○	○	○
Walking a mile	○	○	○	○	○	○
Going up or down 2 flights of stairs (about 20 stairs)	○	○	○	○	○	○
Sitting for 1 hour	○	○	○	○	○	○
Driving for 1 hour	○	○	○	○	○	○

<< Previous   Next >>

DoD-ACT1

Bothersomeness

During the past week, how bothersome have each of the following symptoms been? (choose one response for each symptom)

	Not at all bothersome	Slightly bothersome	Moderately bothersome	Very bothersome	Extremely bothersome
Low back pain	○	○	○	○	○
Leg pain (sciatica)	○	○	○	○	○
Neck pain	○	○	○	○	○

During the past week, how much did pain interfere with your normal work (including both work outside the home and housework)?

- ○ Not at all
- ○ A little bit
- ○ Moderately
- ○ Quite a bit
- ○ Extremely

Next >>

DoD-ACT1

Bothersomeness

If you had to spend the rest of your life with the symptoms you have right now, how would you feel about it?

- ○ Very dissatisfied
- ○ Somewhat dissatisfied
- ○ Neither satisfied nor dissatisfied
- ○ Somewhat satisfied
- ○ Very satisfied

During the past 4 weeks, about how many days did you cut down on the things you usually do for more than half the day because of your low back pain?

[ ] # of days

During the past 4 weeks, how many days did low back pain keep you from going to work or school?

[ ] # of days

<< Previous   Next >>

In the past 7 days...

	Without any difficulty	With a little difficulty	With some difficulty	With much difficulty	Unable to do
Are you able to do chores such as vacuuming or yard work?	○	○	○	○	○
Are you able to go up and down stairs at a normal pace?	○	○	○	○	○
Are you able to go for a walk of at least 15 minutes?	○	○	○	○	○
Are you able to run errands and shop?	○	○	○	○	○

Next >>

DoD-ACT1

Health Survey - PROMIS-29

In the past 7 days...

	Never	Rarely	Sometimes	Often	Always
I felt fearful.	○	○	○	○	○
I found it hard to focus on anything other than my anxiety.	○	○	○	○	○
My worries overwhelmed me.	○	○	○	○	○
I felt uneasy.	○	○	○	○	○

Next >>

DoD-ACT1

Health Survey - PROMIS-29

In the past 7 days...

	Never	Rarely	Sometimes	Often	Always
I felt worthless.	○	○	○	○	○
I felt helpless.	○	○	○	○	○
I felt depressed.	○	○	○	○	○
I felt hopeless.	○	○	○	○	○

<< Previous   Next >>

During the past 7 days...

	Not at all	A little bit	Somewhat	Quite a bit	Very much
I feeel fatigued.	○	○	○	○	○
I have trouble starting things because I am tired.	○	○	○	○	○

In the past 7 days...

	Not at all	A little bit	Somewhat	Quite a bit	Very much
How run-down did you feel on average?	○	○	○	○	○
How fatigued were you on average?	○	○	○	○	○

<< Previous Next >>

DoD-ACT1

Health Survey - PROMIS-29

In the past 7 days...

	Very poor	Poor	Fair	Good	Very good
My sleep quality was...	○	○	○	○	○

In the past 7 days...

	Not at all	A little bit	Somewhat	Quite a bit	Very much
My sleep was refreshing.	○	○	○	○	○
I had a problem with my sleep.	○	○	○	○	○
I had difficulty falling asleep.	○	○	○	○	○

<< Previous   Next >>

DoD-ACT1

Health Survey - PROMIS-29

In the past 7 days...

	Not at all	A little bit	Somewhat	Quite a bit	Very much
I am satisfied with how much work I can do (include work at home).	○	○	○	○	○
I am satisfied with my ability to work (include work at home).	○	○	○	○	○
I am satisfied with my ability to do regular personal and household responsibilities.	○	○	○	○	○
I am satisfied with my ability to perform my daily routines.	○	○	○	○	○

<< Previous   Next >>

DoD-ACT1

Health Survey - PROMIS-29

In the past 7 days...

	Not at all	A little bit	Somewhat	Quite a bit	Very much
How much did pain interfere with your day to day activities?	○	○	○	○	○
How much did pain interfere with work around the home?	○	○	○	○	○
How much did pain interfere with your ability to participate in social activities?	○	○	○	○	○
How much did pain interfere with your household chores?	○	○	○	○	○

<< Previous   Next >>

DoD-ACT1

Health Survey - PROMIS-29

In the past 7 days...

How would you rate your pain on average?

No Pain										Worst Imaginable Pain
○ 0	○ 1	○ 2	○ 3	○ 4	○ 5	○ 6	○ 7	○ 8	○ 9	○ 10

Next >>

Since the beginning of your current episode of pain, from which of the following providers have you sought treatment for your low back pain?
(Choose all that apply)

- ☐ Primary care doctor
- ☐ Medical specialist (specify type below)
- ☐ Doctor of Osteopathy
- ☐ Doctor of Chiropractic
- ☐ Acupuncturist
- ☐ Massage therapist
- ☐ Physical therapist
- ☐ Pain clinic or pain specialist
- ☐ Counselor or mental health specialist
- ☐ N/A
- ☐ Other (specify type below)

Specify: ____________________

During the **past week**, how often have you taken **pain relieving medication** (including prescription and over-the-counter medications or supplements)?

- ○ 0 days
- ○ 1-2 days
- ○ 3-4 days
- ○ 5-6 days
- ○ 7 days

Next >>

DoD-ACT1

Health Care and Medication Use

During the **past week**, have you taken **non-narcotic analgesics**?

Examples: Acetaminophen, Tylenol, Tylenol Extra Strength, Ultram, etc.

- ◯ No
- ◯ Yes, for back pain ONLY
- ◯ Yes, for other reasons ONLY
- ◯ Yes, for back pain AND other reasons

During the **past week**, have you taken **non-steroidal anti-inflammatory drugs (NSAIDS)**?

Examples: Advil, Aleve, Aspirin, Bextra, Celebrex, Disalcid, Excedrin, Excedrin PM, Feldene, Ibuprofen, Indomethacin, Meclomen, Motrin, Maproxen/Naprosyn, Relefen, Sulindac, Trilisate, Tolectic, etc.

- ◯ No
- ◯ Yes, for back pain ONLY
- ◯ Yes, for other reasons ONLY
- ◯ Yes, for back pain AND other reasons

<< Previous   Next >>

DoD-ACT1

Health Care and Medication Use

During the **past week**, have you taken **sedatives or muscles relaxants**?

Examples: Alprazolam, Ambien, Baclofen, Diazepam, Donnatal, Flexeril, Lorazepam, Meprobamate, Methocarbamol/Robaxin, Norflex, Phenergan, Phenobarbital, Skelaxin, Soma, Temazepam, tizanidine, Tranxene, Tylenol PM, Valium, Xanax, Zanaflex, etc.

- ◯ No
- ◯ Yes, for back pain ONLY
- ◯ Yes, for other reasons ONLY
- ◯ Yes, for back pain AND other reasons

During the **past week**, have you taken **narcotic analgesics**?

Examples: ASA w/ Codeine, Darvocet, Darvon, Demerol, Dilaudid, Fentanyl, Meperidine, Morphine, Oxycodone, Percodan, Talwin, Tylenol-3, Tylenol w/ Codeine, Tylox, Vicodin, etc.

- ◯ No
- ◯ Yes, for back pain ONLY
- ◯ Yes, for other reasons ONLY
- ◯ Yes, for back pain AND other reasons

<< Previous   Next >>

During the **past week**, have you taken **anti-depressants**?

Examples: Amitriptyline, Celexa, Desipramine, Doxepin, Effexor, Imipramine, Lexapro, Nortriptyline, Paxil, Prozac/Fluoxetine, Trazodone, Wellbutrin, Zoloft, etc.

- ◯ No
- ◯ Yes, for back pain ONLY
- ◯ Yes, for other reasons ONLY
- ◯ Yes, for back pain AND other reasons

During the **past week**, have you taken **supplements**?

Examples: Chondroitin Sulfate, fish oils, flax seeds/oils, Glucosamine, willow bark, etc.

- ◯ No
- ◯ Yes, for back pain ONLY
- ◯ Yes, for other reasons ONLY
- ◯ Yes, for back pain AND other reasons

<< Previous   Next >>

DoD-ACT1

Tobacco Use

This section only appears if the pt stated they were a tobacco user and signed the consent.

Which of the following topics did your Doctor of Chiropractic talk to you about in the last six weeks .

- ☐ Tobacco-related health problems
- ☐ Setting a date to quit all tobacco use
- ☐ Tips to help you quit all tobacco use
- ☐ Telephone tobacco quit lines
- ☐ Tobacco cessation groups/classes
- ☐ Nicotine patches, nicotine gum, or nicotine lozenges
- ☐ Prescription medications (such as Chantix, Zyban, nicotine inhaler)
- ☐ Natural ways to quit tobacco (such as acupunture, hypnosis, herbal supplements)
- ☐ None of the above

Did you expect to hear advice about quitting tobacco from your Doctor of Chiropractic?

- ○ No
- ○ Yes

How helpful were the topics that you talked about with your Doctor of Chiropractic?

- ○ 1 Not at all helpful
- ○ 2
- ○ 3 Somewhat helpful
- ○ 4
- ○ 5 Very helpful

Next >>

DoD-ACT1

Tobacco Use

Did your Doctor of Chiropractic give you written materials related to tobacco use?

- ◯ No
- ◯ Yes

If Yes,

Did you read them?

- ◯ Did not read them
- ◯ Read all or parts of them

If you read them,

How helpful were the written materials?

- ◯ 1 Not at all helpful
- ◯ 2
- ◯ 3 Somewhat helpful
- ◯ 4
- ◯ 5 Very helpful

Which statement best describes your smoking **during the past 7 days**?

- ◉ I smoked regularly.
- ◯ I smoked once in a while.
- ◯ I have not smoked at all, not even a puff.

For both of these answers, go to 'During the past 7 days, how many cigarettes did you smoke on a typical day?'

Go to Question 'When did you last smoke a cigarette?'

<< Previous   Next >>

DoD-ACT1

Tobacco Use

When did you last smoke a cigarette?

Continue here when 'I have not smoked at all, not even a puff.

- Less than 1 week ago
- 1 - 4 weeks ago
- 1 - 2 months ago
- 2 - 3 months ago

How confident are you that you will remain a non-smoker?

- 1 Not at all confident
- 2
- 3 Somewhat confident
- 4
- 5 Very confident

<< Previous   Next >>

DoD-ACT

Tobacco Use

When you quit smoking, which methods or product did you use?
(check all that apply)

- ☐ Did not make a serious attempt to quit
- ☐ Went cold turkey (quit with no help)
- ☐ Made a quit plan
- ☐ Set a specific date to quit
- ☐ Nicotine patches, nicotine gum or nicotine lozenges
- ☐ Prescription medication (Zyban, Wellbutrin, Chantix)
- ☐ Telephone tobacco quit line
- ☐ Self-help guide
- ☐ Chiropractic adjustment/care
- ☐ Acupuncture/acupressure
- ☐ Massage
- ☐ Hypnosis
- ☐ Biofeedback
- ☐ Meditation/yoga/relaxation/imagery
- ☐ Herbal or botanical supplements
- ☐ Homeopathy
- ☐ Other

If Other, please specify: ______________

<< Previous   Next >>

DoD-ACT1

Tobacco Use

Continue here when 'I smoked regularly' or 'I smoked once in a while' were marked.

During the past 7 days, how many cigarettes did you smoke on a typical day?

(# of cigarettes)

How soon after you wake up do you usually smoke your first cigarette?

- Less than 5 minutes
- 5-30 minutes
- 31-60 minutes
- More than 60 minutes

Do you experience strong cravings to smoke when you have to go without a cigarette for more than an hour or two?

- Never
- Seldom
- Sometimes
- Often
- Always

<< Previous   Next >>

DoD-ACT

Tobacco Use

During the past 6 weeks, how many times have you made a serious attempt to quit smoking (gone at least 24 hours without smoking)?

- ◯ None
- ◯ 1 time
- ◯ 2 times
- ◯ 3 times
- ◯ More than 3 times

If you made an attempt to quit, which of the following methods or products did you use? (check all that apply)

- ☐ Did not make a serious attempt to quit
- ☐ Went cold turkey (quit with no help)
- ☐ Made a quit plan
- ☐ Set a specific date to quit
- ☐ Nicotine patches, nicotine gum or nicotine lozenges
- ☐ Prescription medication (Zyban, Wellbutrin, Chantix)
- ☐ Telephone tobacco quit line
- ☐ Self-help guide
- ☐ Chiropractic adjustment/care
- ☐ Acupuncture/acupressure
- ☐ Massage
- ☐ Hypnosis
- ☐ Biofeedback
- ☐ Meditation/yoga/relaxation/imagery
- ☐ Herbal or botanical supplements
- ☐ Homeopathy
- ☐ Other

If Other, please specify: ____________________

<< Previous Next >>

DoD-ACT 1

Tobacco Use

Are you seriously thinking about quiting smoking in the next 30 days?

- ◯ No
- ◯ Yes

Mark the number that shows how you feel about quitting.

- ◯ 0 No thought of quitting
- ◯ 1
- ◯ 2 Should consider quitting some day
- ◯ 3
- ◯ 4 Should quit but not quite ready
- ◯ 5
- ◯ 6 Thinking about cutting down or quitting
- ◯ 7
- ◯ 8 Have cut down and seriously considering quitting
- ◯ 9
- ◯ 10 Ready to quit now

<< Previous   Next >>

DoD-ACT1

Tobacco Use

Do you also use chewing tobacco or snuff?

- ◯ No
- ◯ Yes

If Yes,

What kind of tobacco is more important to you?

- ◯ Chewing tobacco/snuff
- ◯ Cigarettes

<< Previous   Next >>

DoD-ACT 1

Tobacco Use

Appears if question above was 'yes' or 'yes' on the chewing tobacco/snuff question on baseline.

Which statement best describes your use of chewing tobacco or snuff **during the past 7 days**?

- ◯ I used chewing tobacco/snuff regularly.
- ◯ I used chewing tobacco/snuff once in a while.
- ◯ I have not used chewing tobacco/snuff at all, not even one dip.

For both of these options, go to question 'In a typical day, how often did you use chewing tobacco/snuff, even once?'

Go to question 'When did you last use chewing tobacco or snuff?'

<< Previous | Next >>

DoD-ACT1

Tobacco Use

Continue here when 'I have not used chewing tobacco/ snuff, not even on dip.' was marked.

When did you last use chewing tobacco or snuff?

- ◯ Less than 1 week ago
- ◯ 1 - 4 weeks ago
- ◯ 1 - 2 months ago
- ◯ 2 - 3 months ago

How confident are you that you will remain a non-chewer?

- ◯ 1 Not at all confident
- ◯ 2
- ◯ 3 Somewhat confident
- ◯ 4
- ◯ 5 Very confident

<< Previous   Next >>

DoD-ACT

Tobacco Use

When you quit smoking, which methods or product did you use?
(check all that apply)

- ☐ Did not make a serious attempt to quit
- ☐ Went cold turkey (quit with no help)
- ☐ Made a quit plan
- ☐ Set a specific date to quit
- ☐ Nicotine patches, nicotine gum or nicotine lozenges
- ☐ Prescription medication (Zyban, Wellbutrin, Chantix)
- ☐ Telephone tobacco quit line
- ☐ Self-help guide
- ☐ Chiropractic adjustment/care
- ☐ Acupuncture/acupressure
- ☐ Massage
- ☐ Hypnosis
- ☐ Biofeedback
- ☐ Meditation/yoga/relaxation/imagery
- ☐ Herbal or botanical supplements
- ☐ Homeopathy
- ☐ Other

If Other, please specify: ____________

<< Previous   Next >>

DoD-ACT1

Tobacco Use

Continue here if either 'I use chewing tobacco/snuff regularly' or 'I use chewing tobacco/snuff once in a while' were marked.

In a typical week, how many days do you use chewing tobacco or snuff?

(# of days / week)

How many days does a can/tin/pouch last you?

(# of days)

How soon after you wake up do you use chewing tobacco or snuff?

- Less than 5 minutes
- 5-30 minutes
- 31-60 minutes
- More than 60 minutes

Do you experience strong cravings for a dip/chew when you go more than one hour or two without one?

- Never
- Seldom
- Sometimes
- Often
- Always

<< Previous   Next >>

DoD-ACT

Tobacco Use

During the past 6 weeks, how many times have you made a serious attempt to quit smoking (gone at least 24 hours without smoking)?

- ○ None
- ○ 1 time
- ○ 2 times
- ○ 3 times
- ○ More than 3 times

If you made an attempt to quit, which of the following methods or products did you use? (check all that apply)

- ☐ Did not make a serious attempt to quit
- ☐ Went cold turkey (quit with no help)
- ☐ Made a quit plan
- ☐ Set a specific date to quit
- ☐ Nicotine patches, nicotine gum or nicotine lozenges
- ☐ Prescription medication (Zyban, Wellbutrin, Chantix)
- ☐ Telephone tobacco quit line
- ☐ Self-help guide
- ☐ Chiropractic adjustment/care
- ☐ Acupuncture/acupressure
- ☐ Massage
- ☐ Hypnosis
- ☐ Biofeedback
- ☐ Meditation/yoga/relaxation/imagery
- ☐ Herbal or botanical supplements
- ☐ Homeopathy
- ☐ Other

If Other, please specify: ______

<< Previous   Next >>

DoD-ACT1

Tobacco Use

Are you seriously thinking about quiting chewing tobacco/snuff in the next 30 days?

- ◯ No
- ◯ Yes

Mark the number that shows how you feel about quitting.

- ◯ 0 No thought of quitting
- ◯ 1
- ◯ 2 Should consider quitting some day
- ◯ 3
- ◯ 4 Should quit but not quite ready
- ◯ 5
- ◯ 6 Thinking about cutting down or quitting
- ◯ 7
- ◯ 8 Have cut down and seriously considering quitting
- ◯ 9
- ◯ 10 Ready to quit now

[<< Previous] [Next >>]

DoD-ACT1

Satisfaction with Care

How satisfied are you with your care?

Not at all satisfied — **Extremely satisfied**

( ) 0 ( ) 1 ( ) 2 ( ) 3 ( ) 4 ( ) 5 ( ) 6 ( ) 7 ( ) 8 ( ) 9 ( ) 10

Compared to your first visit, your low back pain is:

- ( ) Completely gone
- ( ) Much better
- ( ) Moderately better
- ( ) A little better
- ( ) About the same
- ( ) A little worse
- ( ) Much worse

Next >>

Self-Reported Care

During the past 2 weeks, what type of care have you had?

- ☐ Medical care
- ☐ Rehabilitation / Physical therapy
- ☐ Chiropractic care
- ☐ None of the above

Next >>

Medical care

What type of doctor did you see:

- ☐ Primary care MD
- ☐ Orthopaedist
- ☐ Neurologist
- ☐ Other (specify below)

Specify: ______________________

How many visits did you have (total)?

______ visits

Did your doctor prescribe medication?

- ○ No
- ○ Yes

Indicate any self-care instructions given by the doctor(s)? (mark all that apply)

- ☐ Exercise
- ☐ Stretching
- ☐ Over-the-counter medication
- ☐ Restrictions on daily living
- ☐ Bed rest
- ☐ Other

<< Previous   Next >>

Chiropractic care

How many visits did you have (total)?

[ ] visits

Indicate the type of care that you received? (mark all that apply)

- [ ] Manipulation/adjustment
- [ ] Self-care instructions
- [ ] Ice
- [ ] Heat
- [ ] Electrical stimulation
- [ ] Ultrasound
- [ ] Soft tissue/massage
- [ ] Tape/bracing
- [ ] Other

<< Previous   Next >>

DoD-ACT1

Self-Reported Care

Self-care

Indicate care that you initiated on your own without the recommendation of a healthcare provider? (mark all that apply)

- ☐ Exercise
- ☐ Over-the-counter medication
- ☐ Nutritional supplements
- ☐ Diet
- ☐ Massage
- ☐ Acupuncture
- ☐ None of the above
- ☐ Other (specify below)

Specify: ____________________

<< Previous   Next >>

DoD-ACT

SECTION
Reactions and Discomforts

During the past 2 weeks, have you seen a healthcare provider for any reason besides routine care*?

- ○ No
- ○ Yes

*Any planned visit to the physician such as preventive care, routine physical exams, or maintenance exams. Please be sure to tell us of any unplanned visit to the doctor or hospital.

If yes, please describe the reason for the visit and any treatment that was provided.

Next >>

DoD-ACT

SECTION
Reactions and Discomforts

Were you hospitalized during the course of treatment?

○ No
◉ Yes

If yes, please describe*:

*Please be sure to include whether it was an ER visit only or not (and length of visit), hospital admission and discharge dates.

<< Previous   Next >>

DoD-ACT

SECTION
Reactions and Discomforts

During the past 2 weeks, have you experienced any discomfort and/or an unpleasant reaction that you think could be connected to the treatment you received in this study?

- ○ No
- ○ Yes

If yes, indicate what discomforts or reactions you experienced.
(check all that apply)

- ☐ Reaction to medication(s)
- ☐ Muscle and/or joint soreness
- ☐ Neck pain
- ☐ Headache
- ☐ Pain or tingling down the arm/hand or leg/foot
- ☐ Mid and upper back
- ☐ Dizziness
- ☐ Broken rib
- ☐ Broken hip
- ☐ Other

<< Previous   Next >>

DoD-ACT

SECTION
Reactions and Discomforts

Reaction to medication(s)

Please describe the study treatment that you believe is connected to this discomfort/reaction:

Were you hospitalized because of this reaction/discomfort?

- ○ No
- ○ Yes

If Yes, please describe*:

*Please be sure to include admission and discharge dates.

<< Previous   Next >>

DoD-ACT

SECTION
Reactions and Discomforts

Reaction to medication(s), continued

Name of medication:

Describe your reaction to the medicine:

How would you rate the amount of discomfort?

No Discomfort **Unbearable Discomfort**

○ 0 ○ 1 ○ 2 ○ 3 ○ 4 ○ 5 ○ 6 ○ 7 ○ 8 ○ 9 ○ 10

<< Previous Next >>

DoD-ACT

SECTION
Reactions and Discomforts

Reaction to medication(s), continued

How long after the treatment did the discomfort/reaction begin?

- ○ Less than 30 minutes
- ○ 30 minutes to 4 hours
- ○ 4 hours to 24 hours
- ○ More than 24 hours

How long did the discomfort last?

- ○ Less than 1 day
- ○ 1 day - 1 week
- ○ More than 1 week
- ○ Ongoing

Did you have to modify your normal daily activities at home and/or work?

- ○ Not at all
- ○ A little
- ○ Moderately
- ○ Could not perform daily activities

If you could not perform daily activities, please explain:

<< Previous   Next >>

DoD-ACT

SECTION
Reactions and Discomforts

Muscle and/or joint soreness

Please describe the study treatment that you believe is connected to this discomfort/reaction:

Were you hospitalized because of this reaction/discomfort?

- ○ No
- ○ Yes

If Yes, please describe*:

*Description should include date of admission and date of discharge.

<< Previous   Next >>

DoD-ACT

SECTION
Reactions and Discomforts

Muscle and/or joint soreness, continued

Describe the discomfort from your muscle and/or joint soreness:

How would you rate the amount of discomfort?

No Discomfort **Unbearable Discomfort**

○ 0 ○ 1 ○ 2 ○ 3 ○ 4 ○ 5 ○ 6 ○ 7 ○ 8 ○ 9 ○ 10

<< Previous Next >>

DoD-ACT

SECTION
Reactions and Discomforts

Muscle and/or joint soreness, continued

How long after the treatment did the discomfort begin?

- ○ Less than 30 minutes
- ○ 30 minutes to 4 hours
- ○ 4 hours to 24 hours
- ○ More than 24 hours

How long did the discomfort last?

- ○ Less than 1 day
- ○ 1 day - 1 week
- ○ More than 1 week
- ○ Ongoing

Did you have to modify your normal daily activities at home and/or work?

- ○ Not at all
- ○ A little
- ○ Moderately
- ○ Could not perform daily activities

If you could not perform daily activities, please explain:

<< Previous   Next >>

DoD-ACT

SECTION
Reactions and Discomforts

Neck Pain

Please describe the study treatment that you believe is connected to this discomfort/reaction:

Were you hospitalized because of this reaction/discomfort?

- ○ No
- ○ Yes

If Yes, please describe*:

*Description should include date of admission and date of discharge.

<< Previous Next >>

DoD-ACT

SECTION
Reactions and Discomforts

Neck pain, continued

Describe the discomfort from your neck pain:

How would you rate the amount of discomfort?

No Discomfort … **Unbearable Discomfort**

○0 ○1 ○2 ○3 ○4 ○5 ○6 ○7 ○8 ○9 ○10

<< Previous   Next >>

DoD-ACT

SECTION
Reactions and Discomforts

Neck pain, continued

How long after the treatment did the discomfort begin?

- ◯ Less than 30 minutes
- ◯ 30 minutes to 4 hours
- ◯ 4 hours to 24 hours
- ◯ More than 24 hours

How long did the discomfort last?

- ◯ Less than 1 day
- ◯ 1 day - 1 week
- ◯ More than 1 week
- ◯ Ongoing

Did you have to modify your normal daily activities at home and/or work?

- ◯ Not at all
- ◯ A little
- ◯ Moderately
- ◯ Could not perform daily activities

If you could not perform daily activities, please explain:

<< Previous   Next >>

DoD-ACT

SECTION
Reactions and Discomforts

Headache

Please describe the study treatment that you believe is connected to this discomfort/reaction:

Were you hospitalized because of this reaction/discomfort?

○ No
○ Yes

If Yes, please describe*:

*Description should include date of admission and date of discharge.

<< Previous Next >>

DoD-ACT

SECTION
Reactions and Discomforts

Headache, continued

Describe the discomfort from your headache:

How would you rate the amount of discomfort?

No Discomfort **Unbearable Discomfort**

○ 0 ○ 1 ○ 2 ○ 3 ○ 4 ○ 5 ○ 6 ○ 7 ○ 8 ○ 9 ○ 10

<< Previous Next >>

DoD-ACT

SECTION
Reactions and Discomforts

Headache, continued

How long after the treatment did the discomfort begin?

- ○ Less than 30 minutes
- ○ 30 minutes to 4 hours
- ○ 4 hours to 24 hours
- ○ More than 24 hours

How long did the discomfort last?

- ○ Less than 1 day
- ○ 1 day - 1 week
- ○ More than 1 week
- ○ Ongoing

Did you have to modify your normal daily activities at home and/or work?

- ○ Not at all
- ○ A little
- ○ Moderately
- ○ Could not perform daily activities

If you could not perform daily activities, please explain:

[ ]

<< Previous   Next >>

DoD-ACT

SECTION
Reactions and Discomforts

Pain or tingling down the arm/hand or leg/foot

Please describe the study treatment that you believe is connected to this discomfort/reaction:

Were you hospitalized because of this reaction/discomfort?

- ○ No
- ○ Yes

If Yes, please describe*:

*Description should include date of admission and date of discharge.

<< Previous Next >>

DoD-ACT

SECTION
Reactions and Discomforts

Pain or tingling down the arm/hand or leg/foot, continued

Describe the discomfort:

How would you rate the amount of discomfort?

No Discomfort **Unbearable Discomfort**

○0 ○1 ○2 ○3 ○4 ○5 ○6 ○7 ○8 ○9 ○10

<< Previous   Next >>

DoD-ACT

SECTION
Reactions and Discomforts

Pain or tingling down the arm/hand or leg/foot, continued

How long after the treatment did the discomfort begin?

- ○ Less than 30 minutes
- ○ 30 minutes to 4 hours
- ○ 4 hours to 24 hours
- ○ More than 24 hours

How long did the discomfort last?

- ○ Less than 1 day
- ○ 1 day - 1 week
- ○ More than 1 week
- ○ Ongoing

Did you have to modify your normal daily activities at home and/or work?

- ○ Not at all
- ○ A little
- ○ Moderately
- ○ Could not perform daily activities

If you could not perform daily activities, please explain:

<< Previous Next >>

DoD-ACT

SECTION
Reactions and Discomforts

Mid and upper back

Please describe the study treatment that you believe is connected to this discomfort/reaction:

Were you hospitalized because of this reaction/discomfort?

- ○ No
- ○ Yes

If Yes, please describe*:

*Description should include date of admission and date of discharge.

<< Previous   Next >>

DoD-ACT

SECTION
Reactions and Discomforts

Mid and upper back, continued

Describe the discomfort:

How would you rate the amount of discomfort?

No Discomfort **Unbearable Discomfort**

○0 ○1 ○2 ○3 ○4 ○5 ○6 ○7 ○8 ○9 ○10

<< Previous Next >>

DoD-ACT

SECTION
Reactions and Discomforts

Mid and upper back, continued

How long after the treatment did the discomfort begin?

- ○ Less than 30 minutes
- ○ 30 minutes to 4 hours
- ○ 4 hours to 24 hours
- ○ More than 24 hours

How long did the discomfort last?

- ○ Less than 1 day
- ○ 1 day - 1 week
- ○ More than 1 week
- ○ Ongoing

Did you have to modify your normal daily activities at home and/or work?

- ○ Not at all
- ○ A little
- ○ Moderately
- ○ Could not perform daily activities

If you could not perform daily activities, please explain:

<< Previous   Next >>

DoD-ACT

SECTION
Reactions and Discomforts

Dizziness

Please describe the study treatment that you believe is connected to this discomfort/reaction:

Were you hospitalized because of this reaction/discomfort?

○ No
○ Yes

If Yes, please describe*:

*Description should include date of admission and date of discharge.

<< Previous   Next >>

DoD-ACT

SECTION
Reactions and Discomforts

Dizziness, continued

Describe the discomfort:

How would you rate the amount of discomfort?

No Discomfort … **Unbearable Discomfort**

○0 ○1 ○2 ○3 ○4 ○5 ○6 ○7 ○8 ○9 ○10

<< Previous   Next >>

DoD-ACT

SECTION
Reactions and Discomforts

Dizziness, continued

How long after the treatment did the discomfort begin?

- ○ Less than 30 minutes
- ○ 30 minutes to 4 hours
- ○ 4 hours to 24 hours
- ○ More than 24 hours

How long did the discomfort last?

- ○ Less than 1 day
- ○ 1 day - 1 week
- ○ More than 1 week
- ○ Ongoing

Did you have to modify your normal daily activities at home and/or work?

- ○ Not at all
- ○ A little
- ○ Moderately
- ○ Could not perform daily activities

If you could not perform daily activities, please explain:

<< Previous Next >>

DoD-ACT

SECTION
Reactions and Discomforts

Broken rib

Please describe the study treatment that you believe is connected to this discomfort/reaction:

Were you hospitalized because of this reaction/discomfort?

○ No
○ Yes

If Yes, please describe*:

*Description should include date of admission and date of discharge.

<< Previous   Next >>

DoD-ACT

SECTION
Reactions and Discomforts

Broken rib, continued

Describe the fracture:

How long after the treatment did you first suspect a fracture/injury?

- ○ Less than 30 minutes
- ○ 30 minutes to 4 hours
- ○ 4 hours to 24 hours
- ○ More than 24 hours

How long was it until you sought medical treatment for the fracture?

- ○ Less than 1 day
- ○ 1 day - 1 week
- ○ More than 1 week
- ○ Ongoing

<< Previous   Next >>

DoD-ACT

SECTION
Reactions and Discomforts

Broken hip

Please describe the study treatment that you believe is connected to this discomfort/reaction:

Were you hospitalized because of this reaction/discomfort?

○ No
○ Yes

If Yes, please describe*:

*Description should include date of admission and date of discharge.

<< Previous   Next >>

DoD-ACT

SECTION
Reactions and Discomforts

Broken hip, continued

Describe the fracture:

How long after the treatment did you first suspect a fracture/injury?

- ○ Less than 30 minutes
- ○ 30 minutes to 4 hours
- ○ 4 hours to 24 hours
- ○ More than 24 hours

How long was it until you sought medical treatment for the fracture?

- ○ Less than 1 day
- ○ 1 day - 1 week
- ○ More than 1 week
- ○ Ongoing

<< Previous   Next >>

DoD-ACT

SECTION
Reactions and Discomforts

Other reaction/discomfort

Please describe the study treatment that you believe is connected to this discomfort/reaction:

Were you hospitalized because of this reaction/discomfort?

- ○ No
- ○ Yes

If Yes, please describe*:

*Description should include date of admission and date of discharge.

<< Previous   Next >>

DoD-ACT

SECTION
Reactions and Discomforts

Other reaction/discomfort, continued

Describe the reaction/discomfort:

How would you rate the amount of discomfort?

No Discomfort **Unbearable Discomfort**

○0 ○1 ○2 ○3 ○4 ○5 ○6 ○7 ○8 ○9 ○10

<< Previous Next >>

DoD-ACT

SECTION
Reactions and Discomforts

Other reaction/discomfort, continued

How long after the treatment did the reaction/discomfort begin?

- ○ Less than 30 minutes
- ○ 30 minutes to 4 hours
- ○ 4 hours to 24 hours
- ○ More than 24 hours

How long did the reaction/discomfort last?

- ○ Less than 1 day
- ○ 1 day - 1 week
- ○ More than 1 week
- ○ Ongoing

How much did the reaction/discomfort affect your normal daily activities at home and/or work?

- ○ Not at all
- ○ A little
- ○ Moderately
- ○ Could not perform daily activities

If you could not perform daily activities, please explain:

<< Previous   Next >>

DoD-ACT

Thank you!

Thank you for your time!

Your next questionnaire will be available on . You will receive a reminder at that time.

Please take this time to verify your contact information is current.

DoD-ACT1

SECTION
-
Pain Rating Scales

Select the number that best describes your low back pain at its **worst** during the **past 24 hours**?

No Pain ... **Worst Possible Pain**

◯0 ◯1 ◯2 ◯3 ◯4 ◯5 ◯6 ◯7 ◯8 ◯9 ◯10

Select the number that best describes your **average** low back pain during the **past week**?

No Pain ... **Worst Possible Pain**

◯0 ◯1 ◯2 ◯3 ◯4 ◯5 ◯6 ◯7 ◯8 ◯9 ◯10

Next >>

Roland Morris Disability Questionnaire

When your back hurts, you may find it difficult to do some of the things you normally do.

This list contains sentences that people have used to describe themselves when they have back pain. When you read them, you may find that some stand out because they describe you **today**. As you read the list, thing of yourself **today**. When you read a sentence that describes how you feel today, choose YES. If the sentence does not describe you, then choose NO.

Remember, only choose YES if you are sure that the sentence describes you **today**.

	No	Yes
I stay home most of the time because of my back.	○	○
I change position frequently to try and get my back comfortable.	○	○
I walk more slowly than usual because of my back.	○	○
Because of my back, I am not doing any jobs that I usually do around the house.	○	○
Because of my back, I use a handrail to get upstairs.	○	○
Because of my back, I lie down to rest more often.	○	○
Because of my back, I have to hold on to something to get out of an easy chair.	○	○
Because of my back, I try to get other people to do things for me.	○	○

Next >>

DoD-ACT1

Roland Morris Disability Questionnaire

Remember, only choose YES if you are sure that the sentence describes you **today**.

	No	Yes
I get dressed more slowly than usual because of my back.	○	○
I only stand up for short periods of time because of my back.	○	○
Because of my back, I try not to bend or kneel down.	○	○
I find it difficult to get out of a chair because of my back.	○	○
My back is painful almost all of the time.	○	○
I find it difficult to turn over in bed because of my back.	○	○
My appetite is not very good because of my back.	○	○
I have trouble putting on my socks (stockings) beause of the pain in my back.	○	○

Go To Next Question >

Skip To Next Section >>

SECTION
Roland Morris Disability Questionnaire

Remember, only choose YES if you are sure that the sentence describes you **today**.

	No	Yes
I only walk short distances because of my back pain.	○	○
I sleep less well because of my back pain.	○	○
Because of my back pain, I get dressed with help from someone else.	○	○
I sit down for most of the day because of my back.	○	○
I avoid heavy jobs around the house because of my back.	○	○
Because of my back pain, I am more irritable and bad tempered with people than usual.	○	○
Because of my back, I go upstairs more slowly than usual.	○	○
I stay in bed most of the time because of my back.	○	○

<< Previous   Next >>

Back Pain Function Scale

On the questions listed below, we are interested in knowing whether you are having **ANY DIFFICULTY** at all with the activities **because of your back problem** for which you are currently seeking attention. Please porvide an answer for each activity.

Today, do you or would you have any DIFFICULTY at all with the following activities BECAUSE OF YOUR BACK PROBLEM?

(choose one response on each line)

	Unable to perform activity	Extreme difficulty	Quite a bit of difficulty	Moderate difficulty	A little bit of difficulty	No difficulty
Any of your usual work, housework, or school activities	○	○	○	○	○	○
Your usual hobbies, recreational, or sporting activities	○	○	○	○	○	○
Performing heavy activities around your home	○	○	○	○	○	○
Bending or stooping	○	○	○	○	○	○
Putting on your shoes or socks (pantyhose)	○	○	○	○	○	○
Lifting a box of groceries from the floor	○	○	○	○	○	○

Next >>

DoD-ACT1

Back Pain Function Scale

Today, do you or would you have any DIFFICULTY at all with the following activities BECAUSE OF YOUR BACK PROBLEM?

(choose one response on each line)

	Unable to perform activity	Extreme difficulty	Quite a bit of difficulty	Moderate difficulty	A little bit of difficulty	No difficulty
Sleeping	○	○	○	○	○	○
Standing for 1 hour	○	○	○	○	○	○
Walking a mile	○	○	○	○	○	○
Going up or down 2 flights of stairs (about 20 stairs)	○	○	○	○	○	○
Sitting for 1 hour	○	○	○	○	○	○
Driving for 1 hour	○	○	○	○	○	○

<< Previous   Next >>

DoD-ACT1

Bothersomeness

During the past week, how bothersome have each of the following symptoms been? (choose one response for each symptom)

	Not at all bothersome	Slightly bothersome	Moderately bothersome	Very bothersome	Extremely bothersome
Low back pain	○	○	○	○	○
Leg pain (sciatica)	○	○	○	○	○
Neck pain	○	○	○	○	○

During the past week, how much did pain interfere with your normal work (including both work outside the home and housework)?

- ○ Not at all
- ○ A little bit
- ○ Moderately
- ○ Quite a bit
- ○ Extremely

Next >>

Bothersomeness

If you had to spend the rest of your life with the symptoms you have right now, how would you feel about it?

- Very dissatisfied
- Somewhat dissatisfied
- Neither satisfied nor dissatisfied
- Somewhat satisfied
- Very satisfied

During the past 4 weeks, about how many days did you cut down on the things you usually do for more than half the day because of your low back pain?

# of days

During the past 4 weeks, how many days did low back pain keep you from going to work or school?

# of days

<< Previous   Next >>

DoD-ACT1

Health Survey - PROMIS-29

In the past 7 days...

	Without any difficulty	With a little difficulty	With some difficulty	With much difficulty	Unable to do
Are you able to do chores such as vacuuming or yard work?	○	○	○	○	○
Are you able to go up and down stairs at a normal pace?	○	○	○	○	○
Are you able to go for a walk of at least 15 minutes?	○	○	○	○	○
Are you able to run errands and shop?	○	○	○	○	○

Next >>

DoD-ACT1

Health Survey - PROMIS-29

In the past 7 days...

	Never	Rarely	Sometimes	Often	Always
I felt fearful.	○	○	○	○	○
I found it hard to focus on anything other than my anxiety.	○	○	○	○	○
My worries overwhelmed me.	○	○	○	○	○
I felt uneasy.	○	○	○	○	○

Next >>

DoD-ACT1

Health Survey - PROMIS-29

In the past 7 days...

	Never	Rarely	Sometimes	Often	Always
I felt worthless.	○	○	○	○	○
I felt helpless.	○	○	○	○	○
I felt depressed.	○	○	○	○	○
I felt hopeless.	○	○	○	○	○

<< Previous   Next >>

DoD-ACT

SECTION
Health Survey - PROMIS-29

During the past 7 days...

	Not at all	A little bit	Somewhat	Quite a bit	Very much
I feel fatigued.	○	○	○	○	○
I have trouble starting things because I am tired.	○	○	○	○	○

In the past 7 days...

	Not at all	A little bit	Somewhat	Quite a bit	Very much
How run-down did you feel on average?	○	○	○	○	○
How fatigued were you on average?	○	○	○	○	○

<< Previous   Next >>

DoD-ACT1

Health Survey - PROMIS-29

In the past 7 days...

	Very poor	Poor	Fair	Good	Very good
My sleep quality was...	○	○	○	○	○

In the past 7 days...

	Not at all	A little bit	Somewhat	Quite a bit	Very much
My sleep was refreshing.	○	○	○	○	○
I had a problem with my sleep.	○	○	○	○	○
I had difficulty falling asleep.	○	○	○	○	○

<< Previous   Next >>

DoD-ACT1

Health Survey - PROMIS-29

In the past 7 days...

	Not at all	A little bit	Somewhat	Quite a bit	Very much
I am satisfied with how much work I can do (include work at home).	○	○	○	○	○
I am satisfied with my ability to work (include work at home).	○	○	○	○	○
I am satisfied with my ability to do regular personal and household responsibilities.	○	○	○	○	○
I am satisfied with my ability to perform my daily routines.	○	○	○	○	○

<< Previous   Next >>

DoD-ACT1

Health Survey - PROMIS-29

In the past 7 days...

	Not at all	A little bit	Somewhat	Quite a bit	Very much
How much did pain interfere with your day to day activities?	○	○	○	○	○
How much did pain interfere with work around the home?	○	○	○	○	○
How much did pain interfere with your ability to participate in social activities?	○	○	○	○	○
How much did pain interfere with your household chores?	○	○	○	○	○

<< Previous   Next >>

In the past 7 days...

How would you rate your pain on average?

No Pain										Worst Imaginable Pain
○0	○1	○2	○3	○4	○5	○6	○7	○8	○9	○10

Next >>

Since the beginning of your current episode of pain, from which of the following providers have you sought treatment for your low back pain?
(Choose all that apply)

- ☐ Primary care doctor
- ☐ Medical specialist (specify type below)
- ☐ Doctor of Osteopathy
- ☐ Doctor of Chiropractic
- ☐ Acupuncturist
- ☐ Massage therapist
- ☐ Physical therapist
- ☐ Pain clinic or pain specialist
- ☐ Counselor or mental health specialist
- ☐ N/A
- ☐ Other (specify type below)

Specify: ______________________

During the **past week**, how often have you taken **pain relieving medication** (including prescription and over-the-counter medications or supplements)?

- ○ 0 days
- ○ 1-2 days
- ○ 3-4 days
- ○ 5-6 days
- ○ 7 days

Next >>

DoD-ACT1

Health Care and Medication Use

During the **past week**, have you taken **non-narcotic analgesics**?

Examples: Acetaminophen, Tylenol, Tylenol Extra Strength, Ultram, etc.

- ◯ No
- ◯ Yes, for back pain ONLY
- ◯ Yes, for other reasons ONLY
- ◯ Yes, for back pain AND other reasons

During the **past week**, have you taken **non-steroidal anti-inflammatory drugs (NSAIDS)**?

Examples: Advil, Aleve, Aspirin, Bextra, Celebrex, Disalcid, Excedrin, Excedrin PM, Feldene, Ibuprofen, Indomethacin, Meclomen, Motrin, Maproxen/Naprosyn, Relefen, Sulindac, Trilisate, Tolectic, etc.

- ◯ No
- ◯ Yes, for back pain ONLY
- ◯ Yes, for other reasons ONLY
- ◯ Yes, for back pain AND other reasons

<< Previous   Next >>

During the **past week**, have you taken **sedatives or muscles relaxants**?

Examples: Alprazolam, Ambien, Baclofen, Diazepam, Donnatal, Flexeril, Lorazepam, Meprobamate, Methocarbamol/Robaxin, Norflex, Phenergan, Phenobarbital, Skelaxin, Soma, Temazepam, tizanidine, Tranxene, Tylenol PM, Valium, Xanax, Zanaflex, etc.

- ◯ No
- ◯ Yes, for back pain ONLY
- ◯ Yes, for other reasons ONLY
- ◯ Yes, for back pain AND other reasons

During the **past week**, have you taken **narcotic analgesics**?

Examples: ASA w/ Codeine, Darvocet, Darvon, Demerol, Dilaudid, Fentanyl, Meperidine, Morphine, Oxycodone, Percodan, Talwin, Tylenol-3, Tylenol w/ Codeine, Tylox, Vicodin, etc.

- ◯ No
- ◯ Yes, for back pain ONLY
- ◯ Yes, for other reasons ONLY
- ◯ Yes, for back pain AND other reasons

<< Previous   Next >>

DoD-ACT

SECTION
Health Care and Medication Use

During the **past week**, have you taken **anti-depressants**?

Examples: Amitriptyline, Celexa, Desipramine, Doxepin, Effexor, Imipramine, Lexapro, Nortriptyline, Paxil, Prozac/Fluoxetine, Trazodone, Wellbutrin, Zoloft, etc.

- No
- Yes, for back pain ONLY
- Yes, for other reasons ONLY
- Yes, for back pain AND other reasons

During the **past week**, have you taken **supplements**?

Examples: vitamins/minerals, fish oil, protein powder, Glucosamine, Chondroitin Sulfate, etc.

- No
- Yes, for back pain ONLY
- Yes, for other reasons ONLY
- Yes, for back pain AND other reasons

DoD-ACT

Tobacco Use

Which of the following topics did your study provider talk to you about in the last three months? (check all that apply)

- Tobacco-related health problems
- Setting a date to quit all tobacco use
- Tips to help you quit all tobacco use
- Telephone tobacco quit lines
- Tobacco cessation groups/classes
- Nicotine patches, nicotine gum, or nicotine lozenges
- Prescription medications (such as Chantix, Zyban, nicotine inhaler)
- Natural ways to quit tobacco (such as acupunture, hypnosis, herbal supplements)
- None of the above
- I have not seen my study provider in the last 3 months

DoD-ACT1

Tobacco Use

Did your Doctor of Chiropractic give you written materials related to tobacco use?

- ◯ No
- ◯ Yes

If Yes,

Did you read them?

- ◯ Did not read them
- ◯ Read all or parts of them

If you read them,

How helpful were the written materials?

- ◯ 1 Not at all helpful
- ◯ 2
- ◯ 3 Somewhat helpful
- ◯ 4
- ◯ 5 Very helpful

Which statement best describes your smoking **during the past 7 days**?

- ◉ I smoked regularly.
- ◯ I smoked once in a while.
- ◯ I have not smoked at all, not even a puff.

For both of these answers, go to 'During the past 7 days, how many cigarettes did you smoke on a typical day?'

Go to Question 'When did you last smoke a cigarette?'

<< Previous   Next >>

DoD-ACT1

Tobacco Use

When did you last smoke a cigarette?

Continue here when 'I have not smoked at all, not even a puff.

- ◯ Less than 1 week ago
- ◯ 1 - 4 weeks ago
- ◯ 1 - 2 months ago
- ◯ 2 - 3 months ago

How confident are you that you will remain a non-smoker?

- ◯ 1 Not at all confident
- ◯ 2
- ◯ 3 Somewhat confident
- ◯ 4
- ◯ 5 Very confident

<< Previous   Next >>

DoD-ACT

Tobacco Use

When you quit smoking, which methods or product did you use?
(check all that apply)

- ☐ Did not make a serious attempt to quit
- ☐ Went cold turkey (quit with no help)
- ☐ Made a quit plan
- ☐ Set a specific date to quit
- ☐ Nicotine patches, nicotine gum or nicotine lozenges
- ☐ Prescription medication (Zyban, Wellbutrin, Chantix)
- ☐ Telephone tobacco quit line
- ☐ Self-help guide
- ☐ Chiropractic adjustment/care
- ☐ Acupuncture/acupressure
- ☐ Massage
- ☐ Hypnosis
- ☐ Biofeedback
- ☐ Meditation/yoga/relaxation/imagery
- ☐ Herbal or botanical supplements
- ☐ Homeopathy
- ☐ Other

If Other, please specify: ______

<< Previous   Next >>

Continue here when 'I smoked regularly' or 'I smoked once in a while' were marked.

During the past 7 days, how many cigarettes did you smoke on a typical day?

(# of cigarettes)

How soon after you wake up do you usually smoke your first cigarette?

- Less than 5 minutes
- 5-30 minutes
- 31-60 minutes
- More than 60 minutes

Do you experience strong cravings to smoke when you have to go without a cigarette for more than an hour or two?

- Never
- Seldom
- Sometimes
- Often
- Always

<< Previous Next >>

DoD-ACT

Tobacco Use

During the past 6 weeks, how many times have you made a serious attempt to quit smoking (gone at least 24 hours without smoking)?

- ○ None
- ○ 1 time
- ○ 2 times
- ○ 3 times
- ○ More than 3 times

If you made an attempt to quit, which of the following methods or products did you use? (check all that apply)

- ☐ Did not make a serious attempt to quit
- ☐ Went cold turkey (quit with no help)
- ☐ Made a quit plan
- ☐ Set a specific date to quit
- ☐ Nicotine patches, nicotine gum or nicotine lozenges
- ☐ Prescription medication (Zyban, Wellbutrin, Chantix)
- ☐ Telephone tobacco quit line
- ☐ Self-help guide
- ☐ Chiropractic adjustment/care
- ☐ Acupuncture/acupressure
- ☐ Massage
- ☐ Hypnosis
- ☐ Biofeedback
- ☐ Meditation/yoga/relaxation/imagery
- ☐ Herbal or botanical supplements
- ☐ Homeopathy
- ☐ Other

If Other, please specify: ____________________

<< Previous   Next >>

Are you seriously thinking about quiting smoking in the next 30 days?

- ◯ No
- ◯ Yes

Mark the number that shows how you feel about quitting.

- ◯ 0 No thought of quitting
- ◯ 1
- ◯ 2 Should consider quitting some day
- ◯ 3
- ◯ 4 Should quit but not quite ready
- ◯ 5
- ◯ 6 Thinking about cutting down or quitting
- ◯ 7
- ◯ 8 Have cut down and seriously considering quitting
- ◯ 9
- ◯ 10 Ready to quit now

<< Previous   Next >>

Do you also use chewing tobacco or snuff?

- ◯ No
- ◯ Yes

If Yes,

What kind of tobacco is more important to you?

- ◯ Chewing tobacco/snuff
- ◯ Cigarettes

<< Previous   Next >>

DoD-ACT1

Tobacco Use

Appears if question above was 'yes' or 'yes' on the chewing tobacco/snuff question on baseline.

Which statement best describes your use of chewing tobacco or snuff **during the past 7 days**?

- ◯ I used chewing tobacco/snuff regularly.
- ◯ I used chewing tobacco/snuff once in a while.
- ◯ I have not used chewing tobacco/snuff at all, not even one dip.

For both of these options, go to question 'In a typical day, how often did you use chewing tobacco/snuff, even once?'

Go to question 'When did you last use chewing tobacco or snuff?'

<< Previous | Next >>

DoD-ACT1

Tobacco Use

Continue here when 'I have not used chewing tobacco/ snuff, not even on dip.' was marked.

When did you last use chewing tobacco or snuff?

- ◯ Less than 1 week ago
- ◯ 1 - 4 weeks ago
- ◯ 1 - 2 months ago
- ◯ 2 - 3 months ago

How confident are you that you will remain a non-chewer?

- ◯ 1 Not at all confident
- ◯ 2
- ◯ 3 Somewhat confident
- ◯ 4
- ◯ 5 Very confident

<< Previous   Next >>

DoD-ACT

Tobacco Use

When you quit smoking, which methods or product did you use?
(check all that apply)

- ☐ Did not make a serious attempt to quit
- ☐ Went cold turkey (quit with no help)
- ☐ Made a quit plan
- ☐ Set a specific date to quit
- ☐ Nicotine patches, nicotine gum or nicotine lozenges
- ☐ Prescription medication (Zyban, Wellbutrin, Chantix)
- ☐ Telephone tobacco quit line
- ☐ Self-help guide
- ☐ Chiropractic adjustment/care
- ☐ Acupuncture/acupressure
- ☐ Massage
- ☐ Hypnosis
- ☐ Biofeedback
- ☐ Meditation/yoga/relaxation/imagery
- ☐ Herbal or botanical supplements
- ☐ Homeopathy
- ☐ Other

If Other, please specify: __________

<< Previous Next >>

DoD-ACT1

Tobacco Use

Continue here if either 'I use chewing tobacco/snuff regularly' or 'I use chewing tobacco/snuff once in a while' were marked.

In a typical week, how many days do you use chewing tobacco or snuff?

[ ] (# of days / week)

How many days does a can/tin/pouch last you?

[ ] (# of days)

How soon after you wake up do you use chewing tobacco or snuff?

- ○ Less than 5 minutes
- ○ 5-30 minutes
- ○ 31-60 minutes
- ○ More than 60 minutes

Do you experience strong cravings for a dip/chew when you go more than one hour or two without one?

- ○ Never
- ○ Seldom
- ○ Sometimes
- ○ Often
- ○ Always

<< Previous   Next >>

DoD-ACT

Tobacco Use

During the past 6 weeks, how many times have you made a serious attempt to quit smoking (gone at least 24 hours without smoking)?

- ○ None
- ○ 1 time
- ○ 2 times
- ○ 3 times
- ○ More than 3 times

If you made an attempt to quit, which of the following methods or products did you use? (check all that apply)

- ☐ Did not make a serious attempt to quit
- ☐ Went cold turkey (quit with no help)
- ☐ Made a quit plan
- ☐ Set a specific date to quit
- ☐ Nicotine patches, nicotine gum or nicotine lozenges
- ☐ Prescription medication (Zyban, Wellbutrin, Chantix)
- ☐ Telephone tobacco quit line
- ☐ Self-help guide
- ☐ Chiropractic adjustment/care
- ☐ Acupuncture/acupressure
- ☐ Massage
- ☐ Hypnosis
- ☐ Biofeedback
- ☐ Meditation/yoga/relaxation/imagery
- ☐ Herbal or botanical supplements
- ☐ Homeopathy
- ☐ Other

If Other, please specify: ____________________

<< Previous   Next >>

DoD-ACT1

Tobacco Use

Are you seriously thinking about quiting chewing tobacco/snuff in the next 30 days?

- ◯ No
- ◯ Yes

Mark the number that shows how you feel about quitting.

- ◯ 0 No thought of quitting
- ◯ 1
- ◯ 2 Should consider quitting some day
- ◯ 3
- ◯ 4 Should quit but not quite ready
- ◯ 5
- ◯ 6 Thinking about cutting down or quitting
- ◯ 7
- ◯ 8 Have cut down and seriously considering quitting
- ◯ 9
- ◯ 10 Ready to quit now

<< Previous   Next >>

DoD-ACT

Thank you!

Congratulations!

You have completed your study participation. We greatly appreciate your volunteering for this study and the military. You have made a difference!

If you have any final questions, please contact your local Project Manager.

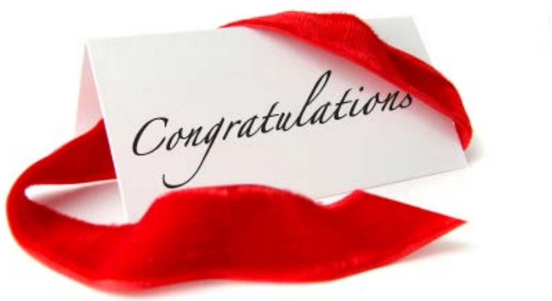

If you are done, you may close this window.

Appendix B: Tobacco Course Handouts

Presentation: Helping your Patients Quit- Guide

Activity 1

Activity 2

Activity 3

DC Quick Reference Guide

Provider Instruction for Quit Plan

The Personal Quit Plan

Quit Plan- Follow-Up

Patient Materials

Presentation: Helping your Patients Quit- Guide

1

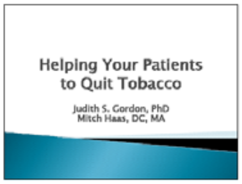

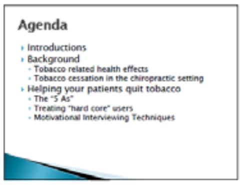

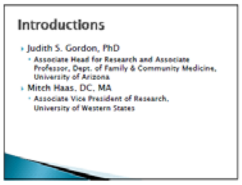

2

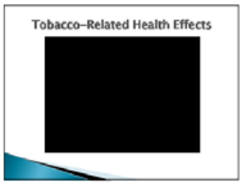

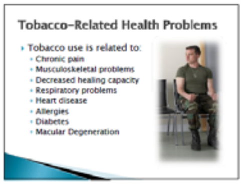

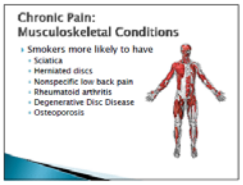

3

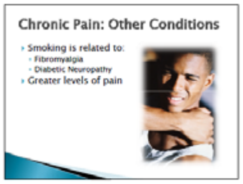

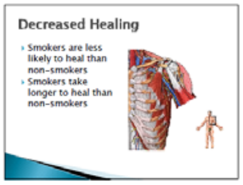

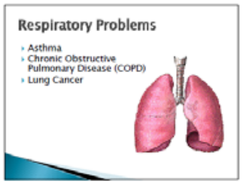

4

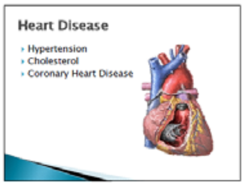

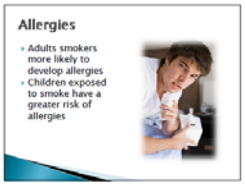

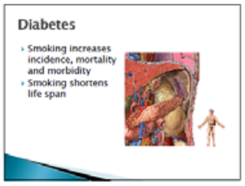

5

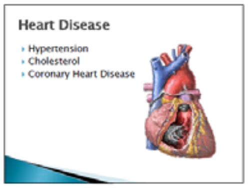

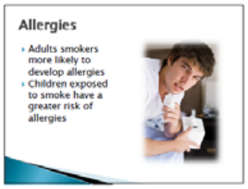

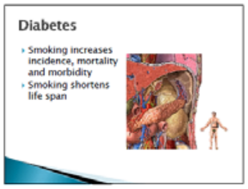

6

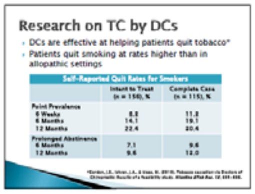

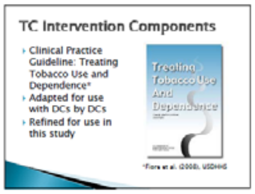

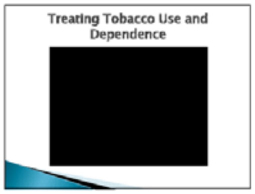

7

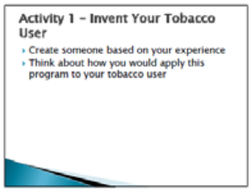

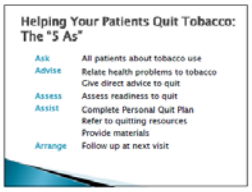

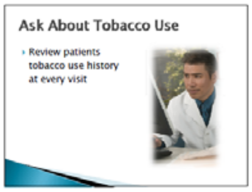

8

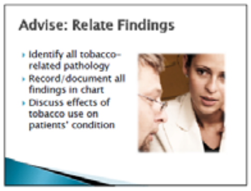

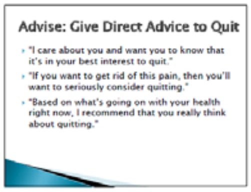

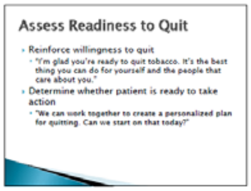

9

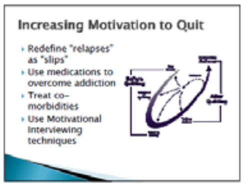

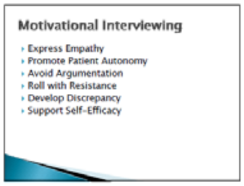

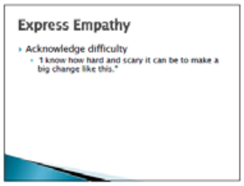

10

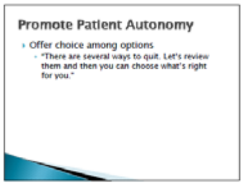

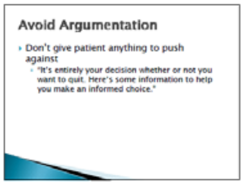

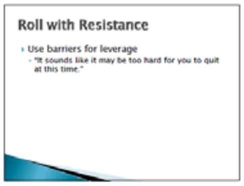

11

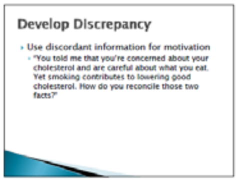

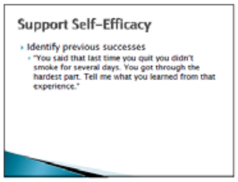

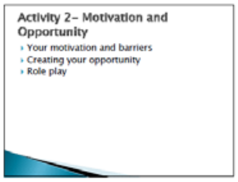

12

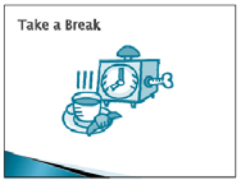

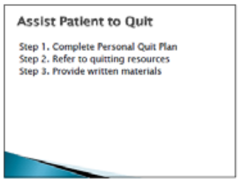

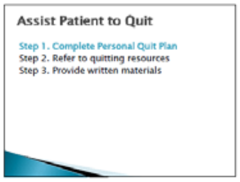

13

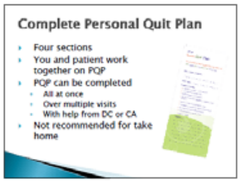

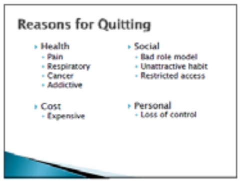

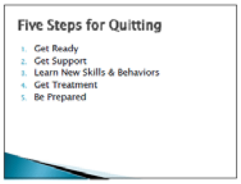

14

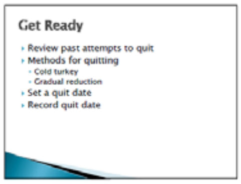

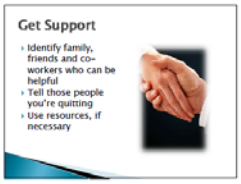

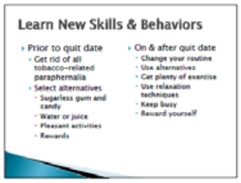

15

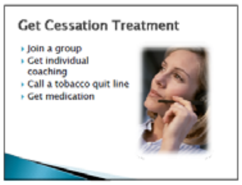

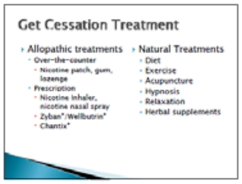

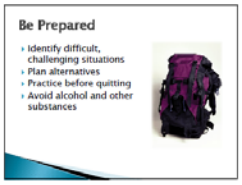

16

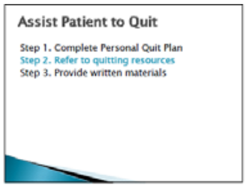

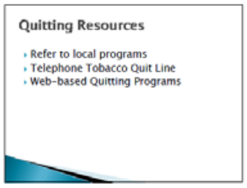

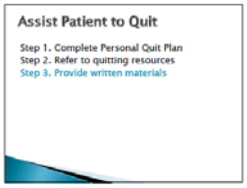

17 18 19

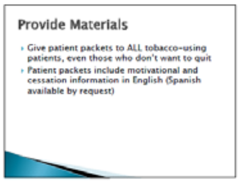
Provide Materials
Give patient packets to ALL tobacco-using patients, even those who don't want to quit
Patient packets include motivational and cessation information in English (Spanish available by request)

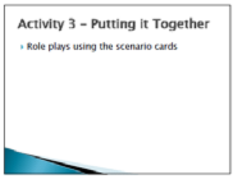
Activity 3 – Putting it Together
Role plays using the scenario cards

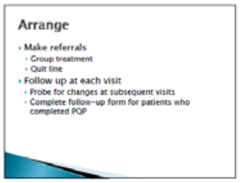
Arrange
Make referrals
Group treatment
Quit line
Follow up at each visit
Probe for changes at subsequent visits
Complete follow-up form for patients who completed PQP

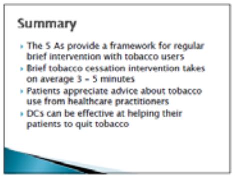
Summary
The 5 As provide a framework for regular brief intervention with tobacco users
Brief tobacco cessation intervention takes on average 3 – 5 minutes
Patients appreciate advice about tobacco use from healthcare practitioners
DCs can be effective at helping their patients to quit tobacco

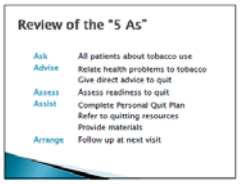
Review of the "5 As"
Ask All patients about tobacco use
Advise Relate health problems to tobacco
Give direct advice to quit
Assess Assess readiness to quit
Assist Complete Personal Quit Plan
Refer to quitting resources
Provide materials
Arrange Follow up at next visit

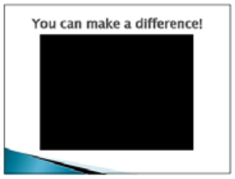
You can make a difference!

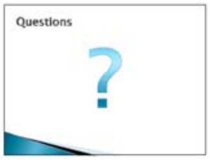
Questions
?

Activity 1 - Invent your tobacco user

You will use this character during your activities today. This is a character you will play as you practice the helping conversation steps. Therefore, it should be someone you feel comfortable representing.

A few tips:

1. Keep it friendly. You will interact with the characters of others.
2. Consider basing your character on someone you know!
3. Remember, 70% of tobacco users are at least thinking of quitting.

My tobacco user:

First Name: ____

Motivators for quitting:

1. ____
2. ____
3. ____

Barriers to quitting:

1. ____
2. ____
3. ____

Readiness to quit:

☐Not ready

☐Thinking of quitting

☐Preparing to quit

☐Making an attempt

Actions you are thinking of taking:

____

____

____

Activity 2 – Motivation and Opportunity

Your motivation and barriers to addressing tobacco use

What are your top 3 reasons to help your patients stop using tobacco?

____

____

____

What are the top 3 barriers you see to helping patients on a regular basis?

____

____

____

What can *you* do to overcome these barriers or work around them?

____

____

____

Creating your opportunity

When do you see yourself bringing up the issue of tobacco use (i.e. at first visit, only after a couple of visits, etc)?

____

____

____

Opening lines

Write down some sample "opening lines" that you could use to start the conversation.

____

____

____

Role-play using invented tobacco user

What did you say or how were you able get any motivators and/or barriers the patient/client had in regards to quitting?

____

____

____

What were the motivators?

____

____

____

What were the barriers?

____

____

____

What did you say or how were you able to help motivate the patient/client to quit?

____

____

____

How did the patient/client respond to your help?

____

____

____

Activity 3 - Role-play using scenario cards

What did you say or how were you able get any motivators and/or barriers the patient/client had in regards to quitting?

____

____

____

What were the motivators?

____

____

____

What were the barriers?

____

____

____

What did you say or how were you able to help motivate the patient/client to quit?

____

____

____

How did the patient/client respond to your help?

____

____

____

What tools did you use or refer to in your conversation (e.g., Personal Quit Plan, referral to quitting resources)?

____

____

References

Fiore MC, Bailey WC, Cohen SJ, et al. *Treating Tobacco Use and Dependence. Clinical Practice Guideline Update.* Rockville, MD: U.S. Department of Health and Human Services. Public Health Service. 2008.

The Cochrane Database of Systematic Reviews 2006
Copyright © 2006 The Cochrane Collaboration. Published by John Wiley and Sons, Ltd.

HELPING YOUR PATIENTS

QUIT TOBACCO

A Guide for Doctors of Chiropractic

Adapted from:
The W.I.S.H. Program
Judith S. Gordon, PhD, PI
Funded by the National Institute on Drug Abuse
Grant #R21-DA021349

Dosing and Administration of Prescription Medication for Tobacco Cessation

Chantix

**Chantix (Varenicline)**	Chantix (varenicline) is a prescription medicine to help adults 18 and over stop smoking. Chantix helps reduce the urge to smoke. Chantix is a non-nicotine pill, that targets nicotine receptors in the brain, attaches to them, and blocks nicotine from reaching them.
**Dosages/Cost**	0.5 mg pill<br>1 mg pill<br>12 weeks duration<br>$120 per 30 day supply w/o insurance
**Dosing**	Week 1<br>Day 1 – 3: 0.5 mg. tablet per day<br>Day 4 – 7: 0.5 mg. tablet twice per day<br>Weeks 2 – 12<br>1 mg. table twice per day
**Duration**	3 months
**Instructions**	Start taking Chantix 7 – 14 days before the Quit Date. The patient can keep smoking during this time. Stop smoking on the Quit Date.<br>Take Chantix after eating and with a full glass (8 ounces) of water.
**Side Effects**	Nausea, sleep problems (trouble sleeping, changes in dreaming), constipation, gas, vomiting.
**Contraindications**	Some people have had changes in behavior, hostility, agitation, depressed mood, suicidal thoughts or actions. Persons with a history of depression of other mental health problems.

Dosing and Administration of Over-the-Counter Medications for Tobacco Cessation

Nicotine Gum

**Nicotine Gum (polacrilex)**	Maximum nicotine levels achieved within 20-30 minutes of chewing.
**Dosages/Cost**	Nicorette - 2 and 4 mg pieces<br>2 mg - $47 / 110 pieces (weekly cost~$24)<br>4 mg - $53 / 110 pieces (weekly cost~$27)<br>Generic nicotine polacrilex (various)<br>2 mg - $30 / 110 pieces (weekly cost~$15)<br>4 mg - $40 / 110 pieces (weekly cost~$20)
**Dosing**	$\geq$ 20 cigs per day, use one 4 mg piece every hour,<br>< 20 cigs per day, use one 2 mg piece every hour.
**Duration**	2-3 months
**Instructions**	Chew until spicy flavor begins, then flatten and "park" between cheek and gum for maximum absorption. Remove after 1/2 hour. Acidic beverages decrease absorption.
**Side Effects**	Jaw fatigue, hiccups, belching, nausea.

Tobacco Cessation Protocol

1. **Ask** all patients about tobacco use (see Sample Assessment below).
2. Relate tobacco use to health issues and **Advise** patients to quit (see page 2).
3. **Assess** patients' readiness to quit (see page 2).
4. If patient is ready to quit, **Assist** them by:
   a. Setting a quit date (see page 2)
   b. Providing written materials
   c. Discussing medications (see pages 6-8)
   d. Providing referrals to tobacco cessation resources
5. **Arrange** for referrals to tobacco cessation resources, and discuss tobacco use at every visit.

Advising Patients to Quit

Example 1: *"I think you should consider quitting smoking now. As your chiropractor, I need you to know that quitting smoking is one of the most important things you can do to protect your health. And quitting may help your back injury to heal more quickly."*

Example 2: *"You've been having on-going problems with back pain. You and I both want your health to improve, so I must advise you that quitting smoking is a crucial part of your treatment."*

Assessing Patients' Readiness to Quit

Example 1: *"Have you thought about quitting in the next few weeks?" If yes: "Are you ready to make a Personal Quit Plan today?"*

Example 2: *"Have you ever tried to quit before?" If yes: "Would you be willing to work with me to make a Personal Quit Plan to quit again?"*

If the patient is not ready to quit, consider using motivational interviewing techniques at each visit to increase the patient's readiness to quit over time.

Setting a Quit Date

Example: *"It's great that you're ready to quit. Pick a date in the next few weeks to be your 'quit date". I'll give you some materials to take home and we can discuss other ways I can help you quit."*

Dosing and Administration of Over-the-Counter Medications for Tobacco Cessation

Nicotine Patch

**Transdermal nicotine patch**	Continuous delivery of nicotine provides constant blood levels. Requires 2-3 days to achieve maximal serum levels.
**Dosages/Cost**	Nicoderm CQ 21, 14, 7 mg/ 24 hr<br>All: $50 / 14 patches (weekly cost~$25)<br>Nicotrol 15mg/16 hr $50 / 14 patches (weekly cost~$25)<br>Other Generic Nicotine Transdermal Patches<br>21, 14, 7 mg - $40 / 28 patches (weekly cost~$20)
**Dosing**	>10 cigs per day, start with highest dose of given brand.<br>5 - 10 cigs per day, use mid-range dose.
**Duration**	6 - 8 weeks. No increase in cessation with longer duration.<br>Suggest:<br>• Weeks 1-4: highest dose of given brand<br>• Weeks 4-6: next lowest dose of brand<br>• Weeks 6-8: lowest dose<br>Taper recommended for psychological reasons, but does not increase efficacy.
**Instructions**	No smoking while on patch, rotate to new hairless skin site each day, remove before bed if insomnia.<br>May consider supplement with 2 mg gum first 48 hrs while plasma levels building.
**Side Effects**	Skin reactions including pruritus, edema, rash; sleep disturbance.<br>Rotate to new site if skin irritation. If irritation persists, switch to gum or lozenge.<br>If sleep disturbance occurs, remove patch before bed and reapply on waking.

Dosing and Administration of Over-the-Counter Medications for Tobacco Cessation

Nicotine Lozenge

**Nicotine Lozenge**	Full dose of nicotine is released gradually by placing a lozenge in the mouth and sucking on it until it dissolves completely.
**Dosages/Cost**	Commit Lozenges<br>2, 4 mg. $40 / 72-count packs (weekly cost~$40)
**Dosing**	9 lozenges/daily during initial 6 weeks of therapy.<br>4 mg if first cigarette within 30 min of awakening; 2 mg if more than 30 min after awakening.<br>1 lozenge every 1-2 hrs for 6 wks, then every 4-8 hrs for last 3 wks.
**Duration**	12 weeks
**Instructions**	Place the lozenge in mouth and allow to dissolve slowly over 20-30 mins. Do not chew, bite, or swallow lozenge. Avoid eating or drinking acidic beverages (i.e., orange juice, coffee) 15 min prior to, during, or after using a lozenge.
**Side Effects**	Headache, diarrhea, flatulence, heartburn, hiccups, nausea, coughing, sore throat, and upper respiratory infection (occurring in > 5% of patients).

Motivation Tips
5 Steps of Motivational Interviewing

Using a few simple techniques can help you to deal with patients who are resistant to change. The following techniques are based on "Motivational Interviewing" developed for use with resistant patients, including tobacco users and other substance abusers.

1. **Express empathy:** Show your patients that you understand how difficult quitting can be.
   "It sounds like you've tried to quit before and the stress was too much to deal with. Quitting may seem impossible sometimes."
2. **Develop discrepancy**: Ask patients about the pros and cons of their tobacco use. Point out inconsistencies.
   "You said that you want you to be rid of the pain in your back, but you don't want to quit smoking. If you want to get better, we may need to do whatever it takes."
3. **Avoid argumentation**: Be flexible with your patient and avoid statements that will encourage disagreement.
   "I care about you, and this issue is so important that I'm willing to discuss your smoking even knowing that you may get angry with me."
4. **Roll with resistance**: Accept those patients who are not ready to quit; let them know it's their decision.
   "And it may very well be that when we're through, you'll decide that it's worth it to keep on smoking as you have been. It may be too difficult to make a change. That will be up to you."
5. **Support self-efficacy:** Help patients set realistic goals. Identify their strengths and skills to quit, and encourage those behaviors.

Most tobacco users attempt to quit "cold turkey" (abrupt cessation of nicotine without tapering, support or use of medication), but only 5% are successful each year. The cessation methods listed below have been shown by research to be effective. Smokers who use

**Cessation Method**	**How it Works**
Advice from health care providers	Brief, simple, advice that includes information, relapse prevention and problem-solving skills.
Telephone counseling	Support, information, and counseling provided via telephone.
Individual and group counseling	Support, information, and counseling provided face-to-face.
Group cessation programs	Group provides support to quit.
Social support	Support from a health care provider, spouse, other relative or friend
Aversion therapy	Pairs smoking with negative effects – rapid smoking requires smokers to take a puff every few seconds until nauseous.
All forms of Nicotine Replacement Therapy	NRT aims to reduce withdrawal symptoms by replacing nicotine in the blood.
Bupropion SR (Zyban®, Wellbutrin®)	Blocks neural re-uptake of dopamine &/or norepinephrinre
Varenicline (Chantix®)	Varenicline (as the tartrate salt) is a partial agonist selective for α4β2 nicotinic acetylcholine receptor subtypes.

these methods are significantly more likely to quit successfully.

The cessation methods listed below may be helpful in assisting some tobacco users to quit, however, insufficient studies (randomized controlled trials) exist to establish their effectiveness.

Cessation Method	How it Works
Exercise	Increasing physical activity minimizes weight gain.
Hypnotherapy	Alters the state of consciousness and aims to weaken the desire to smoke, strengthen the will to quit or help subject to concentrate on quit program.
Acupuncture	Stimulates particular points on the body with the aim of reducing nicotine withdrawal symptoms.
Cold laser therapy	Cold laser beams stimulate the body's acupoints. Aims to release endorphins to simulate the effects of nicotine.
Silver acetate (gum, lozenge & spray)	Produces an unpleasant metallic taste when combined with smoking (aversion therapy).
Herb - Lobelia (Indian tobacco)	Lobeline has effects on the nervous system similar to nicotine. Aims to reduce withdrawal symptoms
Herbs for anxiety (oat straw, skullcap, valerian, lemon balm and vervain.	For treatment of anxiety – aims to lessen desire to smoke.

Provider Instructions for Using the Personal Quit Plan

The Personal Quit Plan provides a framework for chiropractors to personalize a brief 3-5 minute interaction with the patient. This personalized plan can then be given to the patient to take home.

Page 1 of the Personal Quit Plan offers motivational information about the benefits of quitting. Help your patients to identify their own reasons for quitting.

Page 2 offers five steps containing the five key steps for quitting tobacco. Working with the patient, you can easily identify strategies for avoiding and dealing with cessation barriers, as well as learning new skills and behaviors.

Pages 2 – 3 can be used to discuss treatment plans and ways to prevent relapse. It works best when you and your patient complete the plan together.

The Personal Quit Plan also provides space for a plan that may include a follow-up visit and/or referral information, as well as additional resources.

Page 4 can be used to track your patients' progress over time, and help you modify the Quit Plan at subsequent visits.

Adapted from the U.S. Department of Health and Human Services Public Health Service, and the W.I.S.H. Program, Judith S. Gordon, PhD, PI. Funded by the National Institute on Drug Abuse; Grant #R21-DA021349

The Personal Quit Plan

You Can Quit Smoking or Chewing Tobacco!

With Support and Advice from Your Chiropractor

A Personalized Quit Plan for: ______________________________________
Today's Date: ______________________

Reasons for Quitting

I Have Many Good Reasons for Quitting:

- ☐ I will reduce my inflammation and pain.
- ☐ I will shorten my recovery time.
- ☐ I will live longer and live healthier.
- ☐ The people I live with, especially my children, will be healthier.
- ☐ I will have more energy and breathe easier.
- ☐ I will lower my risk of heart attack, stroke, or cancer.
- ☐ My sense of taste and smell will be better.
- ☐ I will feel more in control of my life.
- ☐ I will save money.

Other reasons:

____
____
____
____

How much money I will save:

$_____ (pack or tin price) x ____ (number of packs/tins used per week) = $_____ saved per week

☐ What else could I do with this money:

____
____
____

Five Keys for Quitting

1. Get Ready.

- ☐ I will set a quit date and stick to it—not even a single puff or dip!
- ☐ I will think about past quit attempts. What worked and what did not?

My Quit Date is:

____

2. Get Support and Encouragement.

- ☐ I will tell my family, friends, and coworkers I am quitting.
- ☐ I will talk to my chiropractor &/or other health care providers.
- ☐ I will get group, individual, or telephone counseling.

Who Else Can Help Me Quit:

____
____

3. Learn New Skills and Behaviors.

☐ I will get rid of ALL cigarettes, ashtrays or chewing tobacco in my home, car, or workplace.
☐ When I first try to quit, I will change my routine.
☐ I will stay in nonsmoking areas.
☐ I will breathe in deeply when I feel the urge to smoke or chew. These urges usually last only 3 to 5 minutes. I will try to wait it out, or distract myself e.g. wash my hands, take a shower.
☐ I will plan something enjoyable to do every day.
☐ I will reduce my stress e.g. relax by taking deep breaths, go for a walk.
☐ I will drink a lot of water and other fluids.
☐ I will keep myself busy.
☐ I will reward myself often. Use my "cigarette or chew money" to buy myself a treat.

Other Skills and Behaviors I Can Use:

____
____

4. Get Tobacco Cessation Treatment and Use It Correctly.

- Several effective treatments are now available over-the-counter:
  ☐ **Nicotine gum** ☐ **Nicotine patch** ☐ **Nicotine lozenge**
- Other effective treatments are available by prescription.
- I also recommend the following treatments to help with withdrawal symptoms:

____
____

My Treatment Plan:

Treatment: ____
Instructions: ____
____

5. **Be Prepared for Slips or Difficult Situations.**

If you've tried to quit before, don't give up. Be prepared for challenges, especially in the first few weeks.

☐ I will avoid alcohol.
☐ I will be careful around other smokers or chewers.
☐ I will improve my mood in ways other than smoking or chewing e.g. reward myself, try new activities.
☐ I will eat a healthy diet and stay active.

Other Ways I Will Prepare:

____

Resources: ____
____

Referral: ____

______________________________ ____________

Chiropractor **Date**

The Personal Quit Plan Follow-Up

Patient's Name: ____ Today's Date: ____________

Current tobacco status:

- ❑ Quit on: ________________
- ❑ Returned to Using Tobacco. Quit for: ___________ hours/days
- ❑ Cut down to: _____________ pack(s)/tin(s) per day
- ❑ No change

Quitting Methods Used:

____

____

What Methods Worked Well:

____

____

Successes:

____

____

Challenges:

____

____

Lessons Learned:

____

____

Recommendations:

____

____

Comments:

____

____

____

Patient Materials

Self-Help Cessation Guides

Clearing the Air, National Cancer Institute. Available at:
https://cissecure.nci.nih.gov/ncipubs/detail.aspx?prodid=P133

Spit Tobacco: A Guide for Quitting. National Cancer Institute. Available at:
https://cissecure.nci.nih.gov/ncipubs/detail.aspx?prodid=P121

Motivational Fact Sheets

Harms of Smoking and Health Benefits of Quitting. National Cancer Institute. Available at:
https://cissecure.nci.nih.gov/ncipubs/detail.aspx?prodid=F032

Smokeless Tobacco and Cancer. National Cancer Institute. Available at:
https://cissecure.nci.nih.gov/ncipubs/detail.aspx?prodid=F029

Secondhand Smoke and Cancer. National Cancer Institute. Available at:
https://cissecure.nci.nih.gov/ncipubs/detail.aspx?prodid=F031

Appendix C: Tobacco Cessation DC questionnaire

1. How much training have you personally received in helping your patients quit using tobacco?
   - ❑1 None
   - ❑2 Less than 1 hour
   - ❑3 1-3 hours
   - ❑4 More than 3 hours

---

2. Does your office have printed materials (pamphlets, brochures, guide books) for patients about quitting tobacco?
   - ❑1 No
   - ❑2 Yes

For the next few questions, please answer how often you currently perform various procedures on your patients.

	Never	Rarely	Sometimes	Often	Always
3. Ask about their tobacco use	❑1	❑2	❑3	❑4	❑5
4. Document tobacco use in chart	❑1	❑2	❑3	❑4	❑5
**If they use tobacco, do you:**					
5. Discuss health hazards of tobacco use	❑1	❑2	❑3	❑4	❑5
6. Advise them to quit using tobacco	❑1	❑2	❑3	❑4	❑5
7. Help them to set a specific date to quit	❑1	❑2	❑3	❑4	❑5
8. Create a personal quit plan	❑1	❑2	❑3	❑4	❑5
9. Give written motivational materials	❑1	❑2	❑3	❑4	❑5
10. Give written self-help guide	❑1	❑2	❑3	❑4	❑5
11. Document cessation activities in chart	❑1	❑2	❑3	❑4	❑5
12. Discuss nicotine products (patches, gum, lozenges)	❑1	❑2	❑3	❑4	❑5
13. Discuss prescription medication (Zyban, Wellbutrin, Chantix)	❑1	❑2	❑3	❑4	❑5
14. Schedule a follow-up visit specifically for tobacco use	❑1	❑2	❑3	❑4	❑5
15. Refer to a telephone tobacco quit line	❑1	❑2	❑3	❑4	❑5
16. Refer to individual tobacco cessation counseling	❑1	❑2	❑3	❑4	❑5
17. Refer to group tobacco cessation counseling	❑1	❑2	❑3	❑4	❑5
18. Follow-up after visit by phone or mail	❑1	❑2	❑3	❑4	❑5

Indicate how much you disagree or agree to each of the following statements:

	Strongly Disagree	Somewhat Disagree	Neither Agree nor Disagree	Somewhat Agree	Strongly Agree
19. It is appropriate for Doctors of Chiropractic (DCs) to assess and document their patients' tobacco use.	❑1	❑2	❑3	❑4	❑5
20. DCs should advise patients who smoke cigarettes to quit.	❑1	❑2	❑3	❑4	❑5
21. DCs should assist their patients who smoke to quit.	❑1	❑2	❑3	❑4	❑5
22. I can be effective in helping my patients to quit smoking.	❑1	❑2	❑3	❑4	❑5
23. DCs should advise patients who use smokeless tobacco (chewing tobacco, snuff or snus) to quit.	❑1	❑2	❑3	❑4	❑5
24. DCs should assist their patients who use smokeless tobacco to quit.	❑1	❑2	❑3	❑4	❑5
25. I can be effective in helping my patients to quit using smokeless tobacco.	❑1	❑2	❑3	❑4	❑5
26. I am interested in learning new ways to help patients quit using all forms of tobacco.	❑1	❑2	❑3	❑4	❑5

Please rate the following barriers to incorporating tobacco cessation activities into your practice.

	Not a Barrier				A Large Barrier
27. Patient resistance/complaints	❑1	❑2	❑3	❑4	❑5
28. Amount of time required	❑1	❑2	❑3	❑4	❑5
29. Lack of reimbursement mechanisms	❑1	❑2	❑3	❑4	❑5
30. Staff resistance	❑1	❑2	❑3	❑4	❑5
31. Concerns about effectiveness	❑1	❑2	❑3	❑4	❑5
32. Lack of training in tobacco cessation	❑1	❑2	❑3	❑4	❑5
33. Lack of patient materials	❑1	❑2	❑3	❑4	❑5
34. Lack of referral resources	❑1	❑2	❑3	❑4	❑5
35. Other (specify): ______________________________	❑1	❑2	❑3	❑4	❑5

Which of the following currently describes your tobacco use most currently?
(Please mark one answer under each heading)

36. Smoking

- ❑1 Regular smoker
- ❑2 Occasional smoker
- ❑3 Former smoker
- ❑4 Experimented with smoking
- ❑5 Never smoked

37. Smokeless Tobacco (ST)

- ❑1 Regular ST user
- ❑2 Occasional ST user
- ❑3 Former ST user
- ❑4 Experimented with ST
- ❑5 Never used ST

Appendix D: Assessment Reminder and Follow-Up Protocol

Purpose and Rationale

Data collected are patient-centered outcomes that have been conveniently designed for the participant to complete on any device that has internet. We have created a protocol for the site PM to monitor and assist participants by reminding them when their study assessments are due. Participants are asked to provide email address and phone contact information at the time of consent for this purpose.

Methods for Participant Contact

Email

The web system is designed to automatically send the participant an email on the first day that the assessment is available for completion and on the due date if the assessment has not been completed. The respective site PM are copied on all of these emails. Additional emails may be sent by the site PM as needed to remind participant that the respective assessment is due.

Text Message/Phone Contact

The site PM may also utilize text messaging and/or phone contact as needed to remind participant that assessment is available. The site PM will track methods used for each participant at each assessment.

ACT1 Questionnaire Reminder Scripts

E-mail (-3/-7 days before date due)

Thank you for your continued participation in the ACT Low Back Pain study. As a reminder, we ask that you fill out assessments at week 2, 4, 6, and month 3. It is time for your {week/month} XX assessment. Please complete this assessment by XX/XX/XXXX at https://www.backtoaction.org If you have any questions, please contact me at XX@palmer.edu (site PM's contact information).

Thank you, Site PM https://www.backtoaction.org
Reminder, your login is your email address.

E-mail (Due Date- Day 0)

It has been exactly XX weeks since your enrollment into the ACT study. This means that your {week/month} XX assessment needs to be completed. Please complete this assessment by XX/XX/XXXX at https://www.backtoaction.org. If you have any questions, please contact me at XX@palmer.edu (site PM's contact information).

Thank you, Site PM https://www.backtoaction.org
Reminder, your login is your email address.

Personal Call/Text/Voicemail Message from Site PM (as needed)

Thank you for your continued participation in the ACT study.

As a reminder, your ACT assessment for {week/month} xx needs to be completed by, XX/XX/XXXX. Please go to https://www.backtoaction.org at your earliest convenience to complete. Reminder, your login is your email address.

Thank you, Site PM

Protocol for Collecting Missed Outcomes at Week 6/Month 3:

For outcomes skipped at Week 6 and Month 3, the respective site-PM will attempt to contact the participant by phone to collect the primary outcome measures: Numerical Pain Rating Scales, Roland Morris Disability Scale, and Reaction and Discomforts (see Appendix A). Initial contacts may be made to the participant using text, email or voice message; however, data collected has to be interview style over the telephone. All attempted contacts to the participant will be tracked. This data will be kept on a secure spreadsheet or on the project web-based system, which only site PMs, lead PMs, and CTCC Data Managers have access.

Appendix E: Paper-Based Data Collection Forms

DoD-ACT1
**Participant Name (print):** ______________________________
**Administered by (print):** ____________________
**Signature:** ______________________________
**Clinic:** ______________________________
**Administered on:** __ __ / __ __ / 2 0 __ __ (month / day / year)
**DE UserID:** __ __ __ __ **Date** __ __ **/** __ __ **/ 201** __

Exam

1. Is the low back pain caused by a visceral source or systemic condition (e.g., renal / GI disease, endometriosis, MS, malignancy)? ❑1 No ❑2 Yes

---

2. Is there a condition warranting delay in low back pain treatment? ❑1 No ❑2 Yes

---

3. Is there a history of spinal fracture (past 8 weeks) or spinal surgery (past 12 weeks)? ❑1 No ❑2 Yes

---

4. Do you suspect spinal / paraspinal infection, tumor(s) inflmammatory spondyloarothrapy (i.e. rheumatoid arthritis) or severe osteoporosis? ❑1 No ❑2 Yes

---

5. Is there altered mental capacity compromising health status assessment? ❑1 No ❑2 Yes

---

6. Is further testing / evaluation needed to rule out pathology? ❑1 No ❑2 Yes

---

7. Does the participant have a PTSD classification? ❑1 No ❑2 Yes

---

8. Notes:

Treatment Recommendations: (Mark all that apply)

❑1 Prescription Medications:
- ❑ Muscle relaxants ❑ Narcotic ❑Antidepressants ❑ Anesthetic/Steriods ❑ NSAIDs

❑2 Referral To:
- ❑ Physical Therapy ❑ Chiropractic ❑ Neurology ❑ Orthopedic

❑3 Self-Care Recommendations:
- ❑ OTC medications ❑ Exercises ❑ Behavior Modification ❑ Other

❑4 Other, specify:

ICD Codes (Mark all that apply)

	719.45	Sacroiliac arthralgia / SI joint arthralgia
	721.3	Lumbosacral spondylosis without myelopathy (facet arthrosis) (facet hypertrophy)
	722.93	Disc disorder of lumbar region, other, unspecified, NOS
	722.2	Discogenic Pain
	722.73	Intervertebral disc disorder with myelopathy lumbar
	724.2	Lumbalgia
	724.3	Sciatica
	724.4	Lumbosacral neuritis or radiculitis
	724.8	Lumbar Facet Syndrome (Other symptoms refer to back)
	729.1	Myalgia / myofascial pain
	737.2	Lordosis (acquired) (postural) (hyperlordosis)
	739.3	Nonallopathic lesions of lumbar region NOS
	739.4	Nonallopathic lesions of sacral region NOS
	846.1	Sacroiliac (ligament) sprain
	847.2	Lumbar sprain / strain
	847.3	Sprain of sacrum
	847.4	Sprain of coccyx

ENM / CPT Codes (Mark all that apply)

	99201	Problem Focused New Patient Eval.
	99202	Expanded New Patient Eval.
	99203	Detailed New Patient Eval.
	99204	Comprehensive New Patient Eval.
	99211	Est. Patient Eval. – Minimal
	99212	Est. Patient Eval. – Problem Focused
	99213	Est. Patient Eval. – Expanded
	99214	Ext. Patient Eval. - Detailed
	20550	Drain/inject, ligament/cyst
	20610	Drain/inject joint/bursa
	96372	Intramuscular Injection (Dr. supervised)
	97010	Hot/Cold Packs
	97014	Electrical Muscle Stimulation
	97035	Ultrasound
	97039	Mechanical Massage
	97110	Therapeutic Exercise
	97112	Neuromuscular Re-Ed
	97124	Massage
	97116	Gait training therapy
	97139	Physical (unlisted) medicine procedure
	97140	Manual Therapy Technique (specify)________________
	97140	Trigger Point
	97530	Therapeutic Activities
	97535	Self management training
	97750	Physical performance test

DOD-ACT1

Participant Name (print): ______________________

Administered by (print): ______________________

Signature: ______________________

Administered on: __ __ / __ __ / 2 0 __ __
month day year

DE UserID: __ __ __ __ **Date** __ __ **/** __ __ **/ 201** __

DoD-ACT1

Treatment Form

Spinal Adjustment Details

Level	Technique	Notes:

Modality Details

Area	Specific Therapy	Notes:

Discharged / Released from care: ☐ Yes ☐ No

Tobacco Cessation Details

Mark all that apply:

- ☐1 Not in tobacco cessation study
- ☐2 Did not consult on tobacco cessation today, due to: ______________________
- ☐3 Discussed health hazards of tobacco use
- ☐4 Advised to quit using tobacco
- ☐5 Set a specific date to quit
- ☐6 Created a personal quit plan
- ☐7 Gave written motivational materials
- ☐8 Gave written self-help guide
- ☐9 Discussed nicotine products
- ☐10 Discussed prescription medications
- ☐11 Referred to a telephone quit line
- ☐12 Referred to individual tobacco cessation counseling
- ☐13 Plan to follow-up after visit by phone, text or email
- ☐14 Followed up with patient as part of continuing care / Encouraged patient to stick with quit plan.

ICD Codes *Mark all that apply:*

	719.45	Sacroiliac arthralgia / SI joint arthralgia
	721.3	Lumbosacral spondylosis without myelopathy (facet arthrosis) (facet hypertrophy)
	722.93	Disc disorder of lumbar region, other, unspecified, NOS
	722.2	Discogenic Pain
	722.5	Degeneration of thoracic / lumbar intervertebral disc
	722.73	Intervertebral disc disorder with myelopathy lumbar
	724.2	Lumbalgia
	724.3	Sciatica
	724.4	Lumbosacral neuritis or radiculitis
	724.8	Lumbar Facet Syndrome (Other symptoms refer to back)
	729.1	Myalgia / myofascial pain
	737.2	Lordosis (acquired) (postural) (hyperlordosis)
	739.3	Nonallopathic lesions of lumbar region NOS
	739.4	Nonallopathic lesions of sacral region NOS
	846.1	Sacroiliac (ligament) sprain
	847.2	Lumbar sprain / strain
	847.3	Sprain of sacrum
	847.4	Sprain of coccyx

CPT Codes *Mark all that apply:*

	99201	Problem Focused New Patient Eval.
	99202	Expanded New Patient Eval.
	99203	Detailed New Patient Eval.
	99204	Comprehensive New Patient Eval.
	99211	Est. Patient Eval. – Minimal
	99212	Est. Patient Eval. – Problem Focused
	99213	Est. Patient Eval. – Expanded
	99214	Ext. Patient Eval. - Detailed
	98940	1-2 levels
	98941	3-4 levels
	98942	5 levels
	98943	Extremity
	97012	Traction
	97010	Hot/Cold Packs
	97014	Electrical Muscle Stimulation
	97035	Ultrasound
	97039	Mechanical Massage
	97039	Unlisted Modality__________
	97110	Therapeutic Exercise
	97112	Neuromuscular Re-Ed
	97124	Massage
	97140	Manual Therapy Technique
	97140	Trigger Point
	97530	Therapeutic Activities
	99070A9300	Therabands
	99070A6265	Tape/Immobility Device
	99070A9300	Inflatable Ball
	99070E0230	Ice Packs
	-25	Modifier

Appendix F: Participant Certification

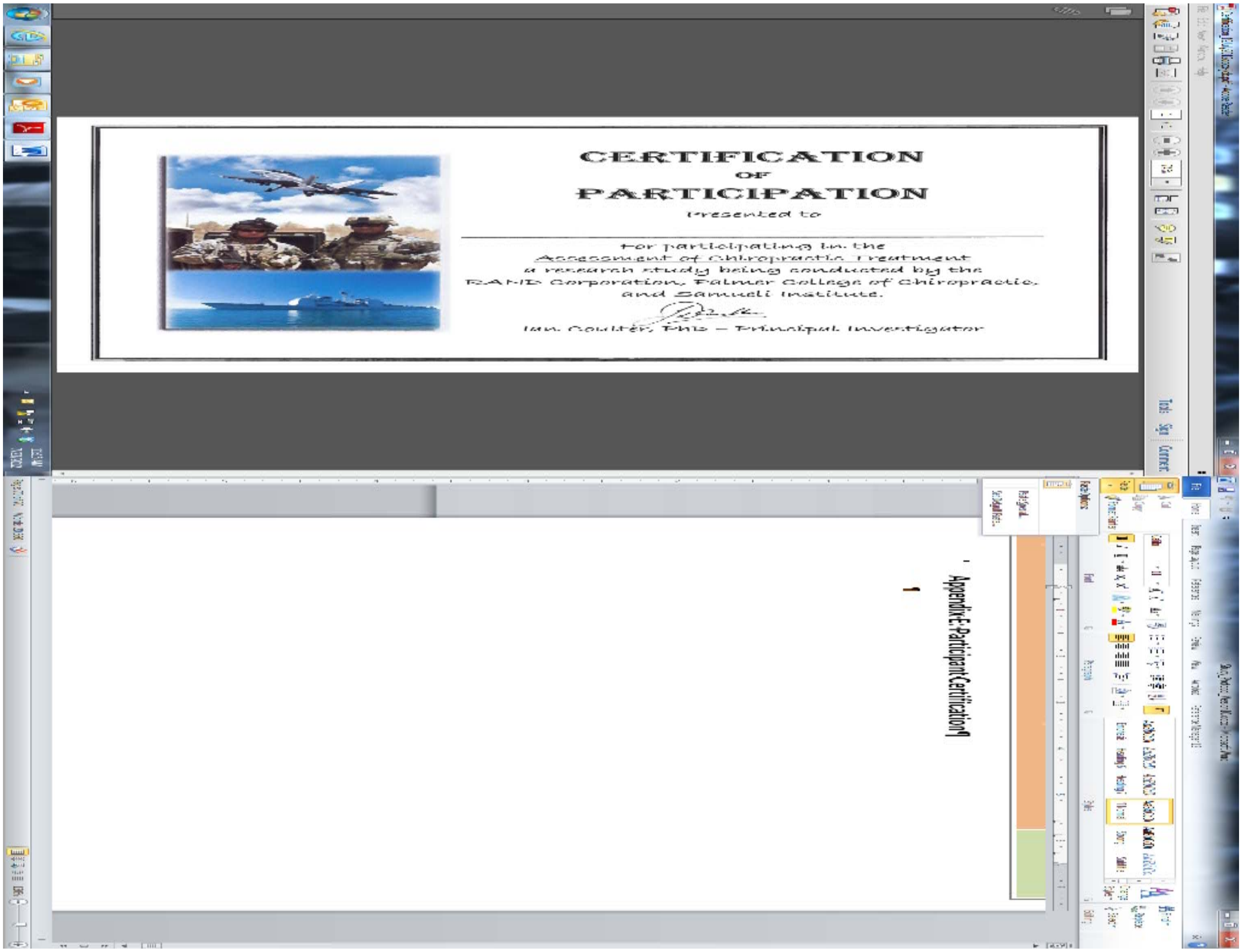

Study Protocol: Data Analysis Plan

Date:	July 2014
Version:	2
Project Title:	Assessment of Chiropractic Treatment for Low Back Pain and Smoking Cessation in Military Active Duty Personnel
Grant Title:	Defense Health Program (DHP) Chiropractic Clinical Trial Award (W81XWH-11-2-0107)
Co-Principal Investigators:	Ian D Coulter, PhD<br>RAND Corp/Samueli Chair in Integrative Medicine<br><br>Christine Goertz, DC, PhD<br>Palmer College of Chiropractic/Vice Chancellor for Research and Health Policy<br><br>Joan Walter, JD, PA<br>Samueli Institute/Vice President, Military Medical Research Programs

CONFIDENTIALITY STATEMENT

The information contained in this document, especially unpublished data, is the property of Palmer College of Chiropractic and is therefore provided to you in confidence as an investigator, potential investigator, or consultant, for review by you, your staff, and an applicable Institutional Review Board/Independent Ethics Committee. It is understood that this information will not be disclosed to others without written authorization from a Co-Principal Investigator listed above.

Revisions:

7/24/2014	Original statistician left the project. The new project biostatistician revised the data analysis plan and submitted it to the Data & Safety Monitoring Committee. No revisions were recommended.

Data Analysis

The analysis team will conduct the data analyses using SAS System for Windows (Release 9.2). They will collaborate with the investigators in presenting and interpreting the results. Descriptive statistics of participant baseline characteristics will be presented for each treatment group to assess their comparability as well as the generalizability of the sample. Descriptive statistics of the primary and secondary outcome variables will be presented for each treatment group at baseline, weeks 2, 4, 6 and month 3.

The primary outcome analysis will focus on the changes from baseline to week 6 since this is the length of the chiropractic care group. Similar analyses will be conducted including the 2 week, 4 week and 3 month waves. Outcome measures will be compared between the chiropractic and medical care only groups at baseline to check for imbalances in the randomization.

A difference-of-differences approach will be used to compare changes over time in the chiropractic arm to changes over time in the conventional medical care only arm. This will be implemented as a regression model rather than by literally modeling change scores. Each study participant will contribute an observation for each wave of data collection. The regression models can be ordinary, logistic, ordered logistic or Poisson depending on the distribution of the outcome measure. As an example we present the logistic version of the model that might be used for a simple satisfied vs. not satisfied survey response.

Model 1. Pre-Post Comparison of Satisfaction between Chiropractic and Medical Care

$$\ln\left(\frac{p_{ijt}}{1-p_{ijt}}\right)=\beta_0+\beta_1\tau_1+\vec{\beta}_2\vec{x}_{ijt}$$

Where p_{ijt} is the probability participant *i* responds as satisfied in treatment arm *j* in time period *t*. *t* takes on 2 values, 0 or 1, for the baseline and 6 week study periods in the basic model. The τ_1 takes on the value 1 in the chiropractic treatment arm at week 6 and takes on the value zero in the medical care arm. τ_1 takes on the value of zero in both treatment arms in the baseline wave. β_1 is an estimate of the chiropractic treatment effect. This model can be easily modified to reflect multiple post time periods (e.g., weeks 2, 4, and month 3). Participant level random effects can be included to control for clustering within participant over time. X can include other patient level covariates to control for imbalance in randomization and differential attrition. It may also be fit for a single outcome type or simultaneously fit across several outcomes to accommodate the correlations between outcomes within participant. Adjusted mean differences and 95% confidence intervals between conventional medical care alone and conventional medical care plus CMT at week 6 will be based on the final models. An intention-to-treat analysis will be used.

We will analyze the data using an intention-to-treat approach in which participants will be analyzed according to their original treatment allocation. All observed data will be used in the analyses. Data analyses will be performed using SAS/STAT (Release 9.3) (SAS Institute Inc., Cary, NC).

The primary outcome variables are numerical pain rating scale (NRS) for low back pain and the Roland-Morris disability questionnaire. These variables will be modeled with linear mixed effects regression over baseline and weeks 2, 4, 6, and 12. We will include terms in the model for time (as a categorical variable), site, group, site-by-group, time-by-group and site-by-time-by-group interactions, and the variables in the minimization algorithm. We will choose the covariance matrix by comparing the maximized log-likelihoods and the Bayesian Information Criteria for several covariance pattern models against the unstructured covariance. Diagnostics of the conditional predicted values and conditional residuals will allow us to assess the assumption of normality and fit for the model.

The main results will be based on the final models for the 2 primary outcome variables at the primary endpoint of 6 weeks. If the site-by-time-by-group interaction is significant at the 0.05 level, results will be reported by site. Adjusted between group means and 95% confidence intervals will be reported for all endpoints using the estimates for the time-by-group interactions. This will allow us to compare the results with those in the pilot study (primary endpoint 4 weeks with a 2 week interim assessment) and to investigate the longer term outcomes.

Secondary analyses will be consistent with the recent NIH Task Force recommendations for a minimum dataset for chronic low back pain. In particular, responder analyses will be conducted for a range of improvement levels at week 6. General estimating equations with a working covariance matrix will be used to estimate the differences in proportions between groups, adjusting for site, site-by-group interaction and the minimization variables.

We will use two approaches to sensitivity analyses to examine the possible effects of missing data on the results obtained from using all observed data. Prior to the conducting the sensitivity analyses, we will identify baseline variables that are predictive of missing outcomes with logistic regression models. Our first approach will be under the assumption that data are missing at random. We will use the Markov Chain Monte Carlo method in SAS Proc MI to impute missing values for each of the primary outcome variables based on the final mixed model covariates, the observed outcome variable at baseline and weeks 2, 3, 6 and 12, and the baseline variables predictive of missing data. We will analyze the resulting datasets for each of 20 imputations with the linear mixed effects models that are fit with all observed data and use SAS Proc MIAnalyze to combine the results. The second approach will be under the assumption that data are missing not at random. Here we will follow the pattern mixture approach described by Carpenter and Kenward (2007). We will first impute missing values as described above for the missing at random approach and then for each participant in each treatment group for each imputation, we will decrease the imputed observation by different amounts representing different patterns of responses. We will then analyze the resulting datasets for each pattern and combine the estimates as described above. If the sensitivity analyses shows us that conclusions differ between the results based on the observed data and that based on full datasets under different missingness assumptions, we will report multiple sets of results.
