# Supplementary material for: Effect of Usual Medical Care Plus Chiropractic Care vs Usual Medical Care Alone on Pain and Disability Among US Service Members With Low Back Pain: A Comparative Effectiveness Clinical Trial
Source: JAMA Netw Open. 2018 May 18;1(1):e180105. doi: 10.1001/jamanetworkopen.2018.0105 (PMC6324527; doi:10.1001/jamanetworkopen.2018.0105)
Supplement: Supplement 2. — eTable 1. Primary and Secondary Outcomes eTable 2. Additional Therapeutic Procedures Delivered by Doctors of Chiropractic for Participants in the Usual Medical Care With Chiropractic Care Group eTable 3. Types of Usual Medical Care eReferences [file jamanetwopen-1-e180105-s002.pdf]

Supplementary Online Content

Goertz CM, Long CR, Vining RD, Pohlman KA, Walter J, Coulter I. Effect of usual medical care plus chiropractic care vs usual medical care alone on pain and disability among US service members with low back pain: a comparative effectiveness clinical trial. *JAMA Netw Open*. 2018;1(1):e180105.
doi:10.1001/jamanetworkopen.2018.0105

eTable 1. Primary and Secondary Outcomes

eTable 2. Additional Therapeutic Procedures Delivered by Doctors of Chiropractic for Participants in the Usual Medical Care With Chiropractic Care Group

eTable 3. Types of Usual Medical Care

eReferences

This supplementary material has been provided by the authors to give readers additional information about their work.

© 2018 Goertz CM et al. *JAMA Network Open.*

eTable 1. Primary and Secondary Outcomes

Outcome Measure	Description	Scaling	Reliability/Validity	MCID
Numerical Rating Scale (NRS)<br>1) Average low back pain (LBP) during the past week (primary outcome)<br>2) Worst low back pain in the past 24 hours	Participants rate using numerical rating scale	0-10, ordinal, 11-box scale (0=no LBP; 10= worst possible LBP).	Von Korff 2000:[1]<br>"The validity of NRSs has been well documented. NRSs demonstrate positive and significant correlations with other measures of pain intensity. They have also demonstrated sensitivity to treatments that are expected to affect pain intensity."	Chou 2007, 2017:[2,3]<br>Small/Slight: 0.5–1.0 points<br>Moderate: >1–2 points<br>Large/Substantial: >2 points<br><br>van der Roer 2006:[4]<br>2.5-4.5 for chronic low back pain patients<br><br>Salaffi 2004:[5]<br>Much better: 2.0<br>Slightly better 1.0<br><br>Lauridsen 2006:[6]<br>1.4 points
Roland Morris Disability Questionnaire	24-item survey used to assess disability-related changes in patients with low back pain	0-24 (Higher score indicated greater disability)	Stratford 2000:[7]<br>Test/retest Reliability: 0.81<br><br>Stratford 2000:[7]<br>Validity (correlation with prognostic rating of change): 0.56<br><br>Riddle 1998:[8]<br>"The area under the Receiver Operating Characteristic curve for the entire Roland Morris Questionnaire scale was 0.68, while the curve areas for smaller Roland Morris Questionnaire intervals varied from 0.80 to 0.97."	Chou 2007, 2017:[2,3]<br>Small/Slight: 1–2 points<br>Moderate: >1–2 points<br>Large/Substantial: >2 points<br><br>Cecchi 2010:[9]<br>2 points between-group<br><br>Cherkin 2011:[10]<br>2.0 between-group on modified RMDQ<br><br>Patrick 1995:[11]<br>2-3 points on modified RMDQ<br><br>Lauridsen 2006:[6]<br>1.7 points

© 2018 Goertz CM et al. *JAMA Network Open.*

Outcome Measure	Description	Scaling	Reliability/Validity	MCID
Low Back Pain Bothersomeness	Participants rate the bothersomeness of low back pain symptoms in the past week	1 to 5 (1=not at all bothersome and 5=extremely bothersome)	Dunn 2005:[12]<br>"Defining "bothersome" LBP as a combination of "extremely bothersome" and "very much bothersome" is the most appropriate.vThis definition of bothersomeness gives a sensitivity of 80% (95% confidence interval [CI] 75% to 84%) and specificity of 61% (95% CI 57% to 65%)."	Cherkin 2011:[10]<br>1.5 between group on 0-10 scale is considered clinically meaningful
Perceived Global Improvement	Participants are asked to rate their perceived low back pain improvement	0-6 (0=completely gone to 6=much worse)	This measure is often an anchor for other outcomes.<br><br>Kamper 2009:[13]<br>"Researchers have also measured patient ratings of the importance of a certain change concurrently with the magnitude of that change. Reported correlations for these measures are high (r=0.7225 and r=0.9026), and this finding also supports the face validity of a global rating of change indicating that gradation along the scale represents a change that is meaningful to the patient. Fischer and colleagues investigated the related concept of clinical relevance and reported strong correlations with patient satisfaction measures (Spearman correlation coefficients 0.56 to 0.77); these figures being significantly higher than those for serial measures. As far as we are aware, only one study has assessed test-retest reliability. Costa et al. reported high ICC values—0.90 (95% CI 0.84 to 0.93)—indicating good reproducibility in a cohort of subjects with chronic low back pain."	We were not able to find a MCID reference for this measure.

© 2018 Goertz CM et al. *JAMA Network Open.*

Outcome Measure	Description	Scaling	Reliability/Validity	MCID
Satisfaction	Participants rate using a numerical rating scale	0-10, ordinal, 11-box scale (0=not at all satisfied; 10=extremely satisfied).	See NRS description above.	We were not able to find a MCID reference for this measure.
Medications	Participants asked how often they took pain relieving medication (both prescription and over-the-counter) during the past week	(0, 1-2, 3-4, 5-6 or 7 days)	N/A	N/A

© 2018 Goertz CM et al. *JAMA Network Open.*

eTable 2. Additional Therapeutic Procedures Delivered by Doctors of Chiropractic for Participants in the Usual Medical Care With Chiropractic Care Group*

	Walter Reed[a]	Pensacola[b]	San Diego[c]
	(n=120)	(n=118)	(n=112)
Hot or cold packs, n (%)	96 (80.0)	68 (57.6)	0
Mechanical traction, n (%)	47 (39.2)	34 (28.8)	0
Electrical Muscle Stimulation, n (%)	94 (78.3)	71 (60.2)	0
Ultrasound, n (%)	1 (0.8)	27 (22.9)	0
Infrared therapy, n (%)	4 (3.3)	0	0
Laser therapy, n (%)	2 (1.7)	2 (1.7)	1 (0.9)
Therapeutic exercise for strength & flexibility, n (%)	19 (15.8)	48 (40.7)	106 (94.6)
Therapeutic exercise for function, n (%)	12 (10.0)	73 (61.9)	0
Other manual therapy, n (%)	50 (41.7)	1 (0.8)	29 (25.9)
Self-care/home management training	20 (16.7)	0	0

*Includes participants that had at least 1 chiropractic visit
[a] Walter Reed National Military Medical Center (Bethesda, Maryland)
[b] Naval Hospital Pensacola (Pensacola, Florida)
[c] Naval Medical Center San Diego (San Diego, California)

© 2018 Goertz CM et al. *JAMA Network Open.*

eTable 3. Types of Usual Medical Care*

	Walter Reed[a]		Pensacola[b]		San Diego[c]	
	**UMC**[d] (n=119)	**UMC+ Chiropractic Care** (n=114)	**UMC** (n=123)	**UMC+ Chiropractic Care** (n=125)	**UMC** (n=31)	**UMC+ Chiropractic Care** (n=27)
Physical therapy referral, n (%)	47 (39.5)	34 (29.8)	23 (18.7)	14 (11.2)	15 (48.4)	13 (48.1)
Pain clinic referral, n (%)	6 (5.0)	4 (3.5)	0	2 (1.6)	1 (3.2)	3 (11.1)
Physical therapy and pain management clinic referrals, n (%)	11 (9.2)	14 (12.3)	0	0	1 (3.2)	2 (7.4)
Prescription for spinal pain medications[e]	67 (56.3)	65 (57.0)	112 (91.1)	111 (88.8)	17 (51.5)	11 (40.7)

*Includes participants that had at least 1 UMC visit
[a] Walter Reed National Military Medical Center (Bethesda, Maryland)
[b] Naval Hospital Pensacola (Pensacola, Florida)
[c] Naval Medical Center San Diego (San Diego, California)
[d] Usual Medical Care
[e] Includes new or changed medications

© 2018 Goertz CM et al. *JAMA Network Open.*

eReferences

1. Von KM, Jensen MP, Karoly P. Assessing global pain severity by self-report in clinical and health services research. *Spine (Phila Pa 1976).* 2000;25:3140-3151.
2. Chou R, Deyo R, Friedly J, Skelly A, Hashimoto R, Weimer M, et al. Nonpharmacologic Therapies for Low Back Pain: A Systematic Review for an American College of Physicians Clinical Practice Guideline. *Ann Intern Med.* 2017;166:493-505.
3. Chou R, Qaseem A, Snow V, Casey D, Cross JT, Jr., Shekelle P, et al. Diagnosis and treatment of low back pain: a joint clinical practice guideline from the American College of Physicians and the American Pain Society. *Ann Intern Med.* 2007;147:478-491.
4. van der Roer N, Ostelo RWJG, Bekkering GE, van Tulder MW, de Vet HCW. Minimal Clinically Important Change for Pain Intensity, Functional Status, and General Health Status in Patients with Nonspecific Low Back Pain. *Spine.* 2006;31:578-582.
5. Salaffi F, Stancati A, Silvestri CA, Ciapetti A, Grassi W. Minimal clinically important changes in chronic musculoskeletal pain intensity measured on a numerical rating scale. *Eur J Pain.* 2004;8:283-291.
6. Lauridsen HH, Hartvigsen J, Manniche C, Korsholm L, Grunnet-Nilsson N. Responsiveness and minimal clinically important difference for pain and disability instruments in low back pain patients. *BMC Musculoskelet Disord.* 2006;7:82.
7. Stratford PW, Binkley JM, Riddle DL. Development and initial validation of the back pain functional scale. *Spine.* 2000;25:2095-2102.
8. Riddle DL, Stratford PW, Binkley JM. Sensitivity to change of the Roland-Morris Back Pain Questionnaire: part 2. *Phys Ther.* 1998;78:1197-1207.
9. Cecchi F, Molino-Lova R, Chiti M, Pasquini G, Paperini A, Conti AA, et al. Spinal manipulation compared with back school and with individually delivered physiotherapy for the treatment of chronic low back pain: a randomized trial with one-year follow-up. *Clin Rehabil.* 2010;24:26-36.
10. Cherkin DC, Sherman KJ, Kahn J, Wellman R, Cook AJ, Johnson E, et al. A comparison of the effects of 2 types of massage and usual care on chronic low back pain: a randomized, controlled trial. *Ann Intern Med.* 2011;155:1-9.
11. Patrick DL, Deyo RA, Atlas SJ, Singer DE, Chapin A, Keller RB. Assessing health-related quality of life in patients with sciatica. *Spine.* 1995;20:1899-1908.

© 2018 Goertz CM et al. *JAMA Network Open.*

12. Dunn KM, Croft PR. Classification of low back pain in primary care: using "bothersomeness" to identify the most severe cases. *Spine (Phila Pa 1976).* 2005;30:1887-1892.

13. Kamper SJ, Ostelo RW, Knol DL, Maher CG, de Vet HC, Hancock MJ. Global Perceived Effect scales provided reliable assessments of health transition in people with musculoskeletal disorders, but ratings are strongly influenced by current status. *Journal of Clinical Epidemiology.* 2010;63:760-766.

© 2018 Goertz CM et al. *JAMA Network Open.*
